# Exploring changes over time and characteristics associated with data retrieval across individual participant data meta-analyses: systematic review

**DOI:** 10.1136/bmj.j1390

**Published:** 2017-04-05

**Authors:** Sarah J Nevitt, Anthony G Marson, Becky Davie, Sally Reynolds, Lisa Williams, Catrin Tudur Smith

**Affiliations:** 1 Department of Biostatistics, University of Liverpool, Liverpool L69 3GL, UK; 2 Department of Molecular and Clinical Pharmacology, University of Liverpool, Liverpool, UK

## Abstract

**Objective:**

To investigate whether the success rate of retrieving individual participant data (IPD) for use in IPD meta-analyses has increased over time, and to explore the characteristics associated with IPD retrieval.

**Design:**

Systematic review of published IPD meta-analyses, supplemented by a reflection of the Cochrane Epilepsy Group’s 20 years’ experience of requesting IPD.

**Data sources:**

Medline, CENTRAL, Scopus, Web of Science, CINAHL Plus, and PsycINFO.

**Eligibility criteria for study selection:**

IPD meta-analyses of studies of all designs and all clinical areas published in English.

**Results:**

760 IPD meta-analyses which identified studies by systematic methods that had been published between 1987 and 2015 were included. Only 188 (25%) of these IPD meta-analyses retrieved 100% of the eligible IPD for analysis, with 324 (43%) of these IPD meta-analyses retrieving 80% or more of relevant IPD. There is insufficient evidence to suggest that IPD retrieval rates have improved over time. IPD meta-analyses that included only randomised trials, had an authorship policy, included fewer eligible participants, and were conducted outside of the Cochrane Database of Systematic Reviews were associated with a high or complete IPD retrieval rate. There was no association between the source of funding of the IPD meta-analyses and IPD retrieval rate. The IPD retrieval rate of the Cochrane Epilepsy Group has declined from 83% (up to 2005) to 65% (between 2012 and 2015) and the reported reasons for lack of data availability have changed in recent years.

**Conclusions:**

IPD meta-analyses are considered to be the “gold standard” for the synthesis of data from clinical research studies; however, only 25% of published IPD meta-analyses have had access to all IPD.

## Introduction

Systematic reviews are considered to provide the greatest amount of evidence to support decision making in medicine.^1^ Individual participant data (IPD) meta-analysis is widely regarded as the “gold standard” approach to the synthesis of clinical trial data, with many documented advantages over traditional aggregate data meta-analyses.^2^ ^3^ ^4^ ^5^ ^6^ ^7^ ^8^ Recent years have shown a noticeable increase in the number of published IPD meta-analyses.^9^ ^10^ ^11^ An average of 49 were published each year between 2005 and 2009,^10^ and recent estimates suggest an increase of around four published IPD meta-analyses per year.^11^ IPD meta-analyses directly influence the design and conduct of clinical trials as well as clinical practice guidelines.^12^ ^13^

Greater resources are required to collect IPD.^5^ ^6^ ^7^ IPD meta-analysis is subject to the risk of selection bias and “availability bias” as only studies for which IPD are made available can be included, and these studies may not be representative of the whole evidence base.^14^ ^15^ IPD meta-analyses may be delayed or abandoned owing to unclear data requesting procedures or barriers to accessing IPD.^16^ ^17^ ^18^ ^19^ Review articles have shown that around a quarter of IPD meta-analyses published up to 2001,^20^ up to 2005,^14^ and even as recently as 2012^11^ obtained IPD for less than 80% of eligible participants. These reviews also reveal poor reporting, particularly the amount of IPD included, with between 10% and 20% of IPD meta-analyses not clearly stating how many studies and participants were eligible, were included in data requests, and were included in the analysis.^9^ ^11^ ^14^ ^20^ In the most recent of these reviews, only 23% of IPD meta-analyses reported the reasons for the unavailability of IPD.^9^

The culture of sharing clinical trial data has changed in recent years. In surveys conducted in 2011,^21^ ^22^ authors of published trials reported an increased willingness to share data compared with an empirical study conducted in 2009.^18^ IPD sharing may be improved by the publication of data transparency strategies and policies by the Institute of Medicine^23^ and European Medicines Agency,^24^ a proposed policy by the International Committee of Medical Journal Editors,^25^ and initiatives across the wider research community as a whole.^22^ ^26^ ^27^ ^28^ Indeed, the launch of data sharing initiatives such as Clinical Study Data Request (CSDR),^29^ a platform allowing researchers to request IPD from nearly 3000 clinical trials from 13 industry sponsors, should make access to IPD easier and faster. However, researchers have reported mixed experiences of using data sharing portals such as CSDR, suggesting that the increased safeguards may have an unintended negative impact on the conduct of IPD meta-analyses.^30^ ^31^ ^32^ ^33^ We examined whether the shift in attitudes and awareness and the increased number of options available for accessing IPD has had a positive impact on IPD meta-analyses. We systematically reviewed all published IPD meta-analyses to investigate whether the success rate of retrieving IPD for the purpose of IPD meta-analyses has increased over time, and explored the characteristics associated with IPD retrieval. We also supplemented these quantitative data by reflecting on the 20 years’ experience of our research group in requesting IPD to undertake IPD meta-analyses in the specialty of epilepsy.

### Systematic review of IPD meta-analyses

#### Methods

We searched Medline, CENTRAL, Scopus, Web of Science, CINAHL Plus, and PsycINFO up to August 2015 using systematic search strategies adapted from the review by Riley et al (web appendix 1).^10^ ^14^ We also consulted the reference lists from the reviews of IPD meta-analyses by Riley et al^10^ ^14^ (provided by the author) and Huang et al^11^ (available as an online appendix).

One author (SJN) screened the title, abstract, and full text of articles identified in electronic searches according to the inclusion and exclusion criteria. The principle reason for exclusion was recorded for relevant articles. Any uncertainties were discussed with CTS and resolved. For accuracy, two authors (BD and SR) also screened a random sample of between 50 and 100 identified articles for eligibility; agreement between the independent screening (SJN and BD or SR) was good and any discrepancies were discussed and resolved.

#### Inclusion and exclusion criteria

IPD meta-analyses of studies of all types (eg, randomised, observational, diagnostic) and all clinical areas that had been published in English were eligible for inclusion. We included articles if IPD was requested from the original study investigators, if IPD was already available to review authors, or if review authors were able to extract IPD from published articles.

Methodological articles, conference abstracts, review protocols, non-clinical reviews (eg, engineering articles) were excluded. Articles including the analysis of IPD from one study as a supplement to an aggregate data meta-analysis or articles in which the primary analysis was not a synthesis (eg, prognostic model validation studies, cost effectiveness analysis) were excluded. Where duplicate publications relating to the same IPD meta-analyses were identified (eg, identical publication across multiple journals), we retained the most recently published article.

#### Data extraction

Information was extracted from eligible IPD meta-analyses using a piloted data extraction form (web appendix 2). The following information was extracted: year of publication, authorship policy, source of funding, clinical area, type of study, type of analysis, number of eligible studies providing IPD or aggregate data, reasons for IPD not being provided, and details of any sensitivity analyses performed to account for missing IPD.

Where published articles presented multiple IPD meta-analyses addressing different research questions with different eligible cohorts for IPD meta-analyses, we extracted information for each IPD meta-analysis. If multiple analyses were presented for the same IPD meta-analyses (eg, analysis of several outcomes), we extracted information on the maximum amount of IPD provided, even if all IPD provided were not used in IPD meta-analyses.

Information was extracted in duplicate from all eligible IPD meta-analyses. One author (SJN) extracted information from all of the eligible IPD meta-analyses and three authors (BD, SR, LW) independently extracted information from a subset of around 40% of the eligible IPD meta-analyses. Agreement between authors was good and any discrepancies were discussed and resolved.

#### Data analysis and presentation of results

Multivariable logistic regression was performed in Stata (version 14) to examine associations between the characteristics of IPD meta-analyses (see box 1) and a high IPD retrieval rate (≥80% compared with < 80% or unknown proportion of IPD provided) or complete IPD retrieval rate (100% compared with <100% or unknown proportion of IPD provided). (See web appendix 3 for further statistical details and several sensitivity analyses exploring the assumptions made in these analyses.)

Box 1 **Characteristics of individual participant data (IPD) meta-analysis**

**Cochrane IPD meta-analyses**

IPD meta-analyses performed as a Cochrane review compared with non-Cochrane IPD meta-analyses

**Number of eligible participants**

For inclusion in IPD meta-analyses (log transformed due to skewed data)

**Authorship policy**

IPD meta-analyses with an authorship policy (individual authorship for those providing IPD, or collaborative group) compared with no authorship policy

**Inclusion of randomised studies only**

In IPD meta-analyses compared with IPD meta-analyses including non-randomised studies, diagnostic test accuracy studies, or a combination of randomised and non-randomised studies

**Commercial source of funding**

IPD meta-analyses with a commercial source of funding (pharmaceutical or manufacturer) compared with non-commercial sources of funding only, no funding, or no information about funding provided

**Age of publication**

Calculated as years before 2016 (log transformed due to skewed data)

Results of multivariable regression are presented as odds ratios and 95% confidence intervals. Other numerical results are presented as medians and ranges or numbers and percentages as appropriate.

#### Patient involvement

No patients were involved in setting the research question or the outcome measures, nor were they involved in the design and implementation of the study. No patients were asked to advise on interpretation or writing up of results. There are no plans to disseminate the results of the research to study participants or the relevant patient community

## Results

### Characteristics of IPD meta-analyses

**Fig 1:**
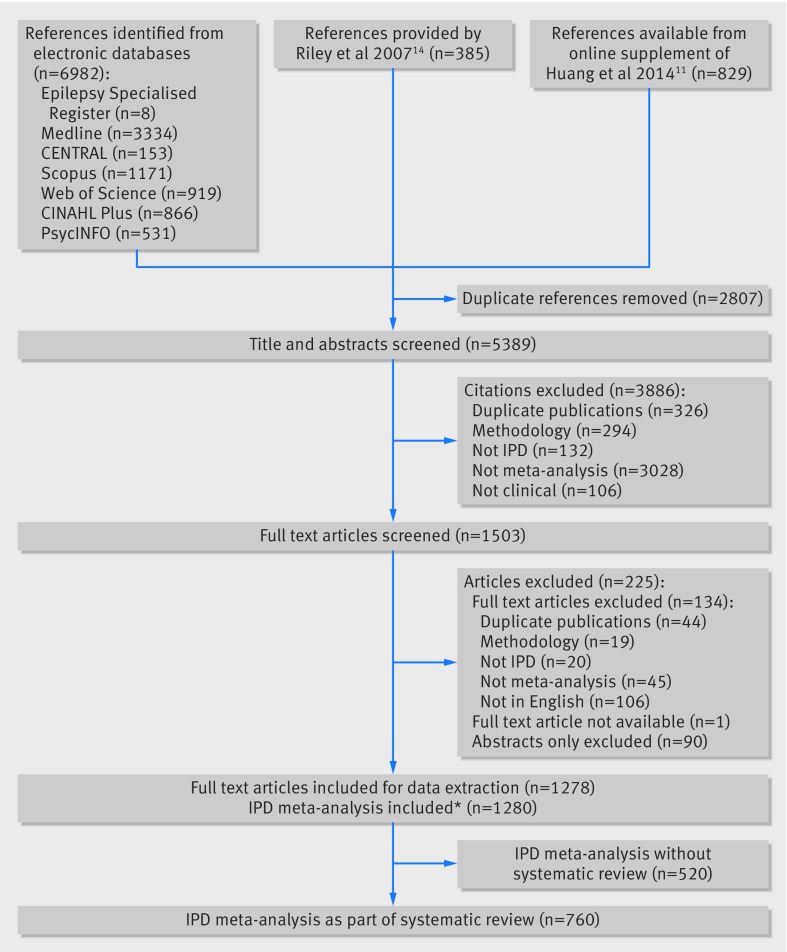
Study flow diagram of identification of eligible individual participant data (IPD) meta-analyses. *Two full text articles each reported two IPD meta-analyses

We identified 1278 eligible articles describing 1280 IPD meta-analyses published to August 2015 (see fig 1 and web appendix 4). IPD retrieval was not relevant for 520 of the IPD meta-analyses and therefore no further results for this subgroup of reviews are reported. These analyses were mostly conducted with the IPD already available to analysts, by collaboration with a group of researchers who had access to the IPD, or by other non-systematic methods of identifying studies for inclusion.

For the remaining 760 IPD meta-analyses, the number of eligible studies was reported in 746 (98%) IPD meta-analyses, with a median of 14 (range 2-923) eligible studies. The number of eligible participants within an IPD meta-analysis was reported in 510 (67%) IPD meta-analyses, with a median of 2369 (range 16-33 369) participants.

### IPD retrieval rate

Out of 760 IPD meta-analyses, IPD were provided from 100% of eligible studies in only 189 (25%) and from 100% of participants in only 188 (25%) (table 1); one IPD meta-analysis using IPD from 100% of studies received an incomplete dataset for one study. IPD from at least 80% of eligible studies was retrieved in 375 (49%) IPD meta-analyses and from 80% of participants in 324 (43%) IPD meta-analyses. IPD was retrieved for less than 50% of studies in 136 (18%) IPD meta-analyses and for less than 50% of participants in 71 (9%) IPD meta-analyses. For 257 IPD meta-analyses, where the number of eligible participants or the number of participants excluded from IPD analysis due to lack of IPD, or both, was not reported, the proportion of IPD retrieved could not be calculated.

**Table 1:** Characteristics of all individual participant data (IPD) meta-analyses according to proportion of IPD provided. Values are numbers (percentages) unless stated otherwise

IPD meta-analysis characteristic*	Total No of IPD meta-analyses	Proportion of IPD retrieved for IPD meta-analysis			
		100%	≥80%	<80%	Unknown†
Clinical area of IPD meta-analysis:					
Breast cancer	40	8 (20)	22 (55)	7 (17)	11 (28)
Cancer (other)	53	14 (26)	27 (51)	14 (26)	12 (23)
Cardiology	105	30 (29)	53 (51)	17 (16)	35 (33)
Central nervous system, neurology, and brain injury	50	13 (26)	20 (40)	14 (28)	16 (32)
Cervical cancer and ovarian cancer	16	1 (6)	7 (44)	1 (6)	8 (50)
Diabetes and endocrinology	30	8 (27)	13 (43)	3 (10)	14 (47)
Gastroenterology, colorectal cancer, and gastric cancer	49	11 (22)	17 (35)	23 (47)	9 (18)
Gynaecology, pregnancy, and neonatology	35	13 (37)	18 (51)	9 (26)	8 (23)
Haematology, leukaemia, and blood cancer	43	11 (26)	20 (47)	4 (9)	19 (44)
Head and neck cancer	16	4 (25)	8 (50)	5 (31)	3 (19)
Hepatitis and liver disease	19	7 (37)	8 (42)	3 (16)	8 (42)
HIV	17	6 (35)	8 (47)	2 (12)	7 (41)
Infection and infectious diseases	31	6 (19)	9 (29)	12 (39)	10 (32)
Injuries and wounds	21	2 (10)	4 (19)	13 (62)	4 (19)
Lung cancer	32	9 (28)	15 (47)	3 (9)	14 (44)
Mental and psychiatric disorders	32	7 (22)	12 (38)	7 (21)	13 (41)
Musculoskeletal and pain	34	9 (26)	11 (32)	5 (15)	18 (53)
Other	26	5 (19)	9 (35)	12 (46)	5 (19)
Otolaryngology, ophthalmology, and periodontology	22	3 (14)	5 (23)	6 (27)	11 (50)
Renal and urology	17	3 (18)	6 (35)	5 (30)	6 (35)
Respiratory and pulmonary	21	7 (33)	11 (52)	3 (15)	7 (33)
Stroke, thrombosis, and hypertension	51	12 (24)	21 (41)	11 (22)	19 (37)
Diagnostic test accuracy	34	5 (15)	9 (26)	8 (24)	17 (50)
Both randomised and non-randomised	68	8 (12)	12 (18)	32 (47)	24 (35)
Type of included studies:					
Diagnostic test accuracy	34	5 (15)	9 (26)	8 (24)	17 (50)
Drug or device	348	102 (29)	183 (53)	73 (21)	92 (26)
Epidemiological	185	38 (21)	58 (31)	44 (24)	83 (45)
Non-drug (interventional)	193	43 (22)	74 (38)	54 (28)	65 (34)
No of eligible studies:					
2-5	102	72 (71)	83 (81)	10 (10)	9 (9)
6-10	174	67 (39)	98 (56)	34 (20)	42 (34)
11-15	120	16 (13)	47 (39)	27 (23)	46 (38)
16-20	87	12 (14)	29 (33)	27 (31)	31 (36)
21-30	101	6 (6)	30 (30)	28 (28)	43 (42)
31-40	50	3 (6)	11 (22)	19 (38)	20 (40)
41-50	29	2 (7)	5 (17)	9 (31)	15 (52)
>50	83	10 (12)	19 (23)	24 (29)	40 (48)
Not stated	14	0 (0)	2 (14)	1 (7)	11 (79)
No of eligible participants:					
≤100	18	14 (78)	16 (94)	1 (6)	0 (0)
101-200	20	13 (65)	16 (80)	4 (20)	0 (0)
201-500	45	21 (47)	25 (56)	19 (42)	1 (2)
501-1000	67	35 (52)	45 (67)	22 (33)	0 (0)
1001-5000	198	70 (35)	134 (68)	61 (31)	3 (1)
5001-10 000	62	13 (21)	37 (60)	23 (37)	2 (3)
>10 000	100	22 (22)	53 (53)	46 (46)	1 (1)
Not stated	250	0 (0)	0 (0)	0 (0)	250 (100)
Type of IPD meta-analysis:					
Cochrane review	64	10 (16)	25 (39)	27 (42)	12 (19)
Non-Cochrane review	696	178 (26)	299 (43)	152 (22)	245 (35)
Authorship policy:					
Individual authorship	243	84 (35)	116 (48)	39 (16)	88 (36)
Collaborative group	264	40 (15)	119 (45)	43 (16)	102 (39)
None	253	64 (25)	89 (35)	97 (39)	67 (26)
Design of included studies:					
Randomised	405	117 (29)	222 (55)	83 (20)	100 (25)
Non-randomised	253	58 (23)	81 (32)	56 (22)	116 (46)
Source of funding:					
Non commercial	383	70 (18)	155 (40)	94 (25)	134 (35)
Commercial	72	26 (36)	37 (51)	14 (20)	21 (29)
Mixed	35	8 (23)	20 (57)	7 (20)	8 (23)
No funding	77	25 (32)	34 (44)	14 (18)	29 (38)
Not stated	193	59 (31)	78 (40)	50 (26)	65 (34)
Total	760	188 (25)	324 (43)	179 (24)	257 (34)

**Fig 2:**
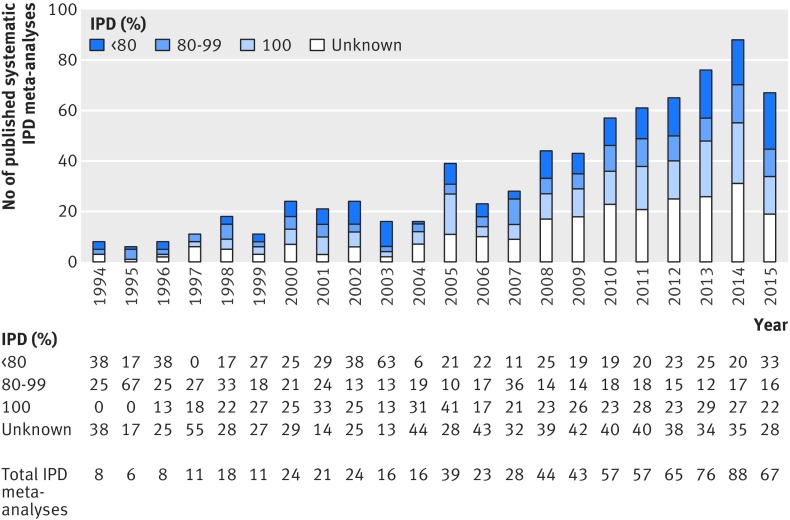
Number of distinct systematic individual participant data (IPD) meta-analyses published to August 2015 and proportion of IPD provided. See table 1 for proportion of IPD meta-analyses providing 100%, 80-99%,and less than 80% of IPD and the proportion of IPD not reported. Six IPD meta-analyses were published from 1987 to 1993; one was provided with less than 80% of IPD, three with 80-99% of IPD, and for two the proportion of IPD provided was not reported

* See figure 2 for proportion of IPD provided in IPD meta-analysis by year.

† IPD meta-analysis where proportion of IPD provided was unknown, where number of eligible participants or number of participants excluded from IPD analysis, or both, owing to lack of IPD was not reported.

Figure 2 shows the number of IPD meta-analyses published by year and the proportion of IPD retrieved.

Table 1 shows the characteristics of the 760 IPD meta-analyses overall and according to IPD retrieval rate (see also web appendix figure). Among the 503 IPD meta-analyses with a known IPD retrieval rate, the odds of retrieving all IPD was statistically significantly higher in non-Cochrane IPD meta-analyses, IPD meta-analyses with a lower number of eligible participants, and IPD meta-analyses with an authorship policy (table 2). Additional analysis considering individual authorship or collaborative group authorship policies showed that the odds of retrieving 80% or more IPD were significantly higher for both types of policy, but that it was only the IPD meta-analyses offering individual authorship that were significantly associated with 100% retrieval of IPD (see web appendix 3).

**Table 2:** Multivariable logistic regression models: characteristics associated with retrieving 100% of individual participant data (IPD) or receiving at least 80% of IPD in 503 IPD meta-analyses

IPD meta-analysis characteristic*	100% of IPD retrieved *v* <100% of IPD			≥80% of IPD retrieved *v* <80% of IPD	
	Odds ratio (95% CI)	P value		Odds ratio (95% CI)	P value
Cochrane IPD meta-analysis	0.40 (0.19 to 0.86)	0.02		0.42 (0.22 to 0.84)	0.01
No of eligible participants†	0.85 (0.80 to 0.90)	<0.001		0.89 (0.84 to 0.94)	<0.001
Authorship policy‡	1.67 (1.07 to 2.59)	0.02		3.37 (2.18 to 5.19)	<0.001
Inclusion of randomised studies only	1.42 (0.92 to 2.18)	0.12		2.74 (1.76 to 4.26)	<0.001
Commercial source of funding§	1.29 (0.76 to 2.19)	0.34		1.04 (0.57 to 1.91)	0.89
Age of publication†	1.08 (0.89 to 1.32)	0.44		1.15 (0.94 to 1.42)	0.18

* See appendix 3 for further statistical details, definitions of characteristics, and results of sensitivity analyses.

† Log transformation applied owing to skewed distribution of data.

‡ IPD meta-analysis with an authorship policy (individual authorship for those providing IPD or collaborative group) compared with no authorship policy.

§ IPD meta-analysis with commercial source of funding (pharmaceutical or manufacturer) compared with non-commercial sources of funding only, no funding, or no information on funding provided.

The odds of retrieving a high proportion (≥80%) of IPD were also significantly higher in IPD meta-analyses of randomised trials only. There was no association between the IPD retrieval rate and source of funding, or the date of publication of IPD meta-analyses (table 2).

### Unavailability of IPD and impact on analysis

Out of the 571 IPD meta-analyses that failed to retrieve IPD from 100% of eligible studies, 201 (35%) had supplemented IPD with aggregate data extracted from study publications. The additional aggregate data had been included from a median of 5 (range 1-541) studies and a median of 683 (range 9-1 180 505) participants.

At least one study had been excluded from the IPD meta-analysis owing to lack of IPD or aggregate data in 419 (55%) IPD meta-analyses. Across these, a median of 4 (range 1-342) studies and a median of 478 (range 8-1 792 339) participants were excluded from IPD meta-analyses, but 241 (32%) IPD meta-analyses failed to state how many participants were excluded from analysis.

Up to six reasons were reported for unavailability of IPD (table 3) for each meta-analysis; unspecific reasons, such as data not available, were reported in 341 (60%) out of 571 IPD meta-analyses. The most common specific reasons for not obtaining IPD were that investigators could not be contacted, investigators had declined to share data, or data had been lost or destroyed. In 24 IPD meta-analyses it was reported that data were not requested for all studies; mainly owing to the size or quality of these studies.

**Table 3:** Reasons reported for unavailability of individual participant data (IPD) in 571 IPD meta-analyses without 100% of IPD (study level) retrieved

Reasons reported for not retrieving 100% of eligible IPD	No (%) of IPD meta-analysis*
Data not available†	341 (60)
Investigators could not be contacted	104 (18)
Investigators declined to share data	74 (13)
Data lost or destroyed	65 (11)
Data could not be extracted‡	55 (10)
Trial was still ongoing	42 (7)
Data quality issues	29 (5)
Failed to provide data in time for IPD meta-analyses	26 (5)
Data not requested	24 (4)
Ethical or ownership restrictions	15 (3)
Reason unclear	11 (2)

* 189 IPD meta-analyses with 100% of IPD provided not included in table. IPD meta-analyses reported up to six reasons for unavailability of IPD. Therefore total number of reasons is greater than 571 and total percentages sum to >100.

† Reason “data not available” corresponds to a statement in the review that IPD were not available for a proportion of studies, without any specific reason given.

‡ Reason applicable only in a small number of IPD meta-analyses where IPD were extracted from publications rather than requested.

In 143 (25%) of the 571 IPD meta-analyses there was no acknowledgment of potential bias resulting from missing IPD. In 199 (34%) of the IPD meta-analyses additional analyses using aggregate data had been performed and in a further 66 (11%) a narrative description of the studies without IPD or a narrative comparison with an aggregate data meta-analysis had been provided (table 4). The remaining 183 (31%) IPD meta-analyses make reference to the missing data; some acknowledging that this may result in bias, without any further investigation of the implication on the conclusions of the review.

**Table 4:** Approach reported to account for missing individual patient data (IPD) in 571 IPD meta-analyses without 100% of IPD (study level) retrieved. Values are numbers (percentages) unless stated otherwise

Approach reported to account for missing IPD	No of IPD meta-analyses*
None stated	143 (25)
Additional analyses performed using aggregate data:	199 (34)
Separate meta-analyses are conducted including IPD only and IPD plus available aggregate data	81 (14)
Aggregate data included in primary analysis	61 (11)
Sensitivity analysis with aggregate data performed	57 (10)
Narrative description of studies without IPD or narrative comparison with aggregate data meta-analysis had been provided:	66 (11)
Results from studies without IPD summarised narratively	48 (8)
Narrative comparison with aggregate data meta-analysis	18 (3)
Stated that missing IPD are a limitation of the meta-analysis or that availability bias might be present, or both	76 (13)
Stated that missing IPD are unlikely to change results	56 (10)
Stated that majority of data are included in analysis	47 (8)
Intend to include data in an update	14 (2)

* 189 IPD meta-analyses with 100% of IPD provided not included in table. IPD meta-analyses described up to three approaches to account for missing IPD. Therefore total number of approaches is greater than 571 and total percentages sum to >100.

### Changes in data sharing over time in epilepsy

The Cochrane Epilepsy Group has been requesting IPD from authors of trials of antiepileptic drug monotherapy since the mid-1990s. Eight reviews for IPD meta-analyses of pair wise antiepileptic drug comparisons have been published since 2000.^34^ ^35^ ^36^ ^37^ ^38^ ^39^ ^40^ ^41^

It is believed that with effective antiepileptic drug treatment, at least 70% of people with active epilepsy have the potential to become seizure-free and go into long term remission shortly after starting treatment with one antiepileptic drug. More than 50 drugs are available worldwide for the treatment of epileptic seizures. The correct choice of first line drug for those with newly diagnosed epilepsy is of great importance, and evidence for the relative effectiveness and tolerability of antiepileptic drugs appropriate to given seizure types should be considered.^42^

IPD is particularly desirable for meta-analysis of trials on antiepileptic drugs to allow complete reanalysis of important time to event outcomes such as time to withdrawal of randomised treatment owing to poor seizure control or adverse effects, the recommended primary effectiveness outcome of drug monotherapy trials,^43^ and to allow investigation of interaction between treatment and epilepsy type, as well as other potential prognostic factors of interest.^44^ The group has also published an IPD network meta-analysis including participants randomised to one of eight antiepileptic drugs in the earlier phase of reviews.^45^ This network meta-analysis is now currently being expanded as a full Cochrane review of 10 antiepileptic drugs.^42^

Web appendix 5 shows IPD retrieval rates and reasons given to us for the unavailability of IPD (where applicable) categorised by the year in which requests for IPD were initiated and according to the type of study sponsorship (industry, government, and academic studies). Academic studies were defined as those conducted within a university or hospital setting without clear industry or government sponsorship or involvement.

### Early data requesting and data sharing experiences

For the reviews and network meta-analyses published up to 2007,^34^ ^45^ ^46^ ^47^ ^48^ ^49^ ^50^ ^51^ we requested IPD for a total of 5887 participants from 29 randomised trials and we successfully received IPD for 4703 (80%) participants from 18 (62%) of these eligible trials. In addition, we had IPD available from our own SANAD trial,^52^ ^53^ the largest trial in epilepsy at the time, in which 2437 participants were randomised. More than 90% of IPD requested from industry and government sponsored studies were successfully received (data provided for 3695 out of 4084 participants from 12 (86%) out of 14 studies). Only 56% of IPD requests from academic studies were successfully received (data provided for 1008 out of 1803 participants from 6 (40%) out of 15 studies) (web appendix 5).

We failed to retrieve IPD from 11 (38%) eligible trials recruiting 1184 participants. For most of these trials, data had been lost or was no longer available due to the elapsed time (web appendix 5).

Many of the data requests were initiated at a time (in 1990s) when IPD meta-analyses designs were relatively novel and when email was not commonly used. Requests to trial investigators were made by letter, fax, telephone, and in person. Some datasets supplied had never been computerised. Because of the informal nature of many of these requests, no data sharing agreements were exchanged and little documentation was retained about the response time to data requests. Therefore, we are unable to make formal numerical comparisons between early and recent data requests; all comparisons are anecdotal.

### Recent data requesting and data sharing experiences

**Fig 3:**
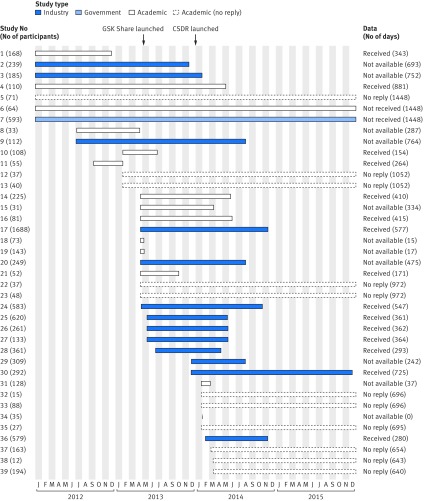
Duration and outcome of data requests for 39 randomised controlled trials of antiepileptic drugs. CSDR=ClinicalStudyDataRequest.com

Since our original network meta-analysis, additional antiepileptic drugs have been used in clinical practice and additional clinical trials have been conducted which has prompted the need to update our original network meta-analysis. We carried out a new search for clinical trials and this identified 39 further eligible trials to be included with the previously received IPD (total of 68 trials).^42^ Requests for IPD for the additional eligible studies began in January 2012 and the database was closed at the end of 2015 to begin analysis (fig 3). In total, IPD for 8261 participants from 39 additional trials were requested. Four of the requests for industry studies were made through ClinicalStudyDataRequest.com (CSDR) (known as GSK Share between May 2013 and January 2014). All other requests were made directly to the relevant sponsor.

For each trial meeting our inclusion criteria, we sent a data request to the first or corresponding author of the trial, or both, or to the trial sponsor, as appropriate. Requests were sent by as many methods as possible (email, post, fax). In the event of no response, we sent a follow-up communication to the author or sponsor previously contacted. If we still received no response, we attempted to contact another trial author or sponsor where possible.

At the close of the database at the end of December 2015, IPD had been received for 5335 (65%) participants from 15 (38%) clinical trials (web appendix 5). For these trials, the median time from initial request to receiving a response was similar for the 24 academic studies (343 days (range 15-861 days)) and the 14 industrial studies (363 days (range 17**-**725 days)) The time taken to receive IPD for one trial (study 27) using CSDR was 364 days. We note that the request was first submitted in June 2013 when the platform was newly initiated and processes were still under development. Current response times from CSDR may differ.

We failed to retrieve IPD from 24 trials conducted between 1989 and 2012. We were provided with a reason in 11 trials that had recruited 1537 participants; the median time from initial request to negative response from these 11 studies was 287 days (range 0-764 days).

Reasons for negative response were: (a) restrictions specific to a country over anonymisation of data (one request submitted to CSDR for an industry study conducted in 2005), (b) cost of retrieving and preparing data was prohibitive owing to the age of the study (two requests submitted to CSDR for industry studies conducted in 2002 and 2007), (c) data could not be made available, no more specific details were provided (three requests directly to industry for studies conducted between 1997 and 2007), (d) concerns about ethical approval for sharing data (one academic author, study conducted 2011), (e) the data we requested had not been recorded (one academic author, study conducted 2005), and (f) data were lost (three academic authors of studies conducted between 1992 and 2012; one of which provided additional unpublished summary data).

For the remaining 13 trials, two (one government sponsored and one academic) had indicated an initial positive response to our IPD requests, but data were not provided by the close of database, whereas 11 studies (nine academic and two industry sponsored) gave no response. The 13 data requests were closed at a median of 972 days (range 640-1448 days) after initial request (fig 3, web appendix 5).

Therefore at the close of the database, the total number of IPD provided for network meta-analysis was 10 038 (71%) of 14 148 eligible participants from 33 (49%) of 68 eligible studies (the initial 29 studies supplemented with the 39 studies described in this article).

## Discussion

Recent years have shown an increase in development of statistical methods for the synthesis of individual participant data (IPD)^54^ as well as a rapid increase in the uptake of methods, with the number of systematic and non-systematic IPD meta-analyses published each year increasing to an average of 105 yearly between 2009 and 2015 compared with 49 yearly between 2005 and 2009.^10^ However, these rapid increases do not seem to be mirrored by improved IPD retrieval rates, which may be partly owing to the increasing uptake of IPD meta-analyses across a wide range of clinical areas and settings where it might be difficult to obtain IPD.

The first in the series of Cochrane Epilepsy Group IPD meta-analyses was published in 2000 when such an approach was relatively new and methods were limited.^34^ This meta-analysis included IPD from 63% of total studies and 83% of total participants, a good retrieval rate in the wider context of all IPD meta-analyses. The success rate has declined from more than 80% (up to 2005) to 65% (between 2012 and 2015), which is a concern. The findings of our systematic review showed that all or a high proportion of IPD from Cochrane reviews were less likely to be retrieved than for non-Cochrane reviews. This might be explained by the inclusion of thorough search methods within Cochrane reviews, as well as advances in systematic searching of larger electronic databases generally, leading to the identification of larger numbers of studies, including more grey literature studies where IPD may be difficult to retrieve with the resources available to review authors, such as Cochrane review authors who usually undertake systematic reviews on a voluntary basis.

Also of concern are changes in the reported reasons for data not being available. Our results show that loss of datasets is a problem for academic trials and has been for many years, highlighting a need for better methods of data collection and archiving. In our more recent requests, the prohibitive costs have prevented the sharing of pharmaceutical data. Additional costs and resources associated with IPD meta-analyses are generally considered to be incurred by the meta-analysts^5^ ^6^ ^7^; however, in this new era of commercial data sharing platforms^29^ and requirement for high level anonymisation of data, costs to data providers are likely to have increased and should be considered when planning an IPD meta-analysis. Collaboration, financial or otherwise, between meta-analysts and data providers may assist in sharing costs and resources, potentially maximising retrieval rates of IPD.

The findings of our systematic review also showed that IPD meta-analyses with an authorship policy, ideally concerning individual authorship, were associated with a high or complete IPD retrieval rate. This is an important finding as the implementation of an authorship policy as an incentive to participate in an IPD meta-analysis, as a feature of a well designed project, is a factor which is in control of the IPD meta-analysis team; even where other characteristics such as study design and number of eligible participants for IPD meta-analyses are constrained by the research question.

Despite our highlighted concerns, recent changes in methods of data sharing have resulted in several benefits to our analyses. Our most common reason for not being able to retrieve data for academic trials was because we failed to make contact with data providers. In our experience, facilities within industry data sharing platforms allowed a clear and transparent pathway of communication between data requestors and providers. The continued benefit of such facilities will require increasing uptake of such platforms from both data users and data providers, from industry, government, and academia.

In addition to improvements to good clinical practice guidelines, developed jointly with regulations such as the European Union Clinical Trials Directive,^55^ a greater focus on data privacy and additional preparation required to share a dataset has resulted in cleaner datasets provided to us in recent requests compared with previous requests. While under the new framework of data sharing platforms, additional time and resources must allow for constructing a research proposal, independent scientific review, signing of data sharing agreements, and anonymisation of data. Recent datasets provided to us have required much less data cleaning before analysis than in previous years, which has led to a reduction in the time required to perform an IPD meta-analysis.

### Strengths and weaknesses of this study

To the best of our knowledge at the time of writing, our systematic review includes the largest cohort of published IPD meta-analyses to date. We aimed to systematically identify all published IPD meta-analyses regardless of use of a systematic design to identify studies, resulting in a large cohort of nearly 1300 IPD meta-analyses. Our inclusion criteria were wide and reasons for exclusion were documented for all references identified in electronic searches. We were unable to include 90 abstracts as they could not be matched to full text articles, despite our best efforts. Because of the size of the cohort of this study, double reference screening and data extraction were performed on only a subset of the articles. Agreement was good and all discrepancies were minor and easily resolved; therefore we believe that any errors during screening and extraction would be minimal and unlikely to influence the overall findings of the study.

We were unable to systematically investigate the IPD retrieval methods employed within the IPD meta-analyses; for example, number of attempts to contact investigators to request data, owing to the lack of published detail on such processes. Data collection methods are likely to impact on the proportion of IPD provided for analysis, and the clearer reporting of approaches to IPD collection, such as the approach of our research group that we have outlined, may prove valuable to those planning new IPD meta-analyses.

Within our primary analysis, we performed multivariable logistic regression analysis on 100% of IPD retrieved and 80% or more of IPD retrieved. We note the limitation of dichotomisation; however, we believe that any loss of information is reduced by the size of the cohort included in analysis, and we have performed a range of sensitivity analyses to investigate all assumptions we have made in our primary analysis, showing consistency and robustness in our results (see web appendix 3 for rationale and numerical results of all sensitivity analyses performed).

We emphasise when interpreting the timelines of our requests between 2012 and 2015, that data sharing policies and platforms were under development, and that all of the industrial sponsors we contacted directly at the time of request have since committed to CSDR or an equivalent data sharing platform such as YODA (Yale University Open Data Access).^55^

### Relation to other studies and implications

Our results show that a quarter of IPD meta-analyses published since 1987 retrieved all IPD for analysis and only half retrieved at least 80% of relevant IPD. This latter finding is higher than previous results, which reported that around 25% of IPD meta-analyses had included less than 80% of IPD.^9^ ^11^ ^14^ ^20^ However, previous work has been based on smaller cohorts of IPD meta-analyses, mostly focused on IPD meta-analyses of randomised controlled trials only, and been conducted over shorter time frames.

In line with previous work,^9^ ^11^ ^14^ ^20^ our results show that important inadequacies in the conduct and reporting of IPD meta-analyses remain. Non-systematic methods, mostly based on the known availability of IPD, had been used to select eligible studies for inclusion in 41% of the initial cohort of IPD meta-analyses that we identified. It was outside the scope of this study to further examine the design of these analyses; however, we recommend that non-systematic pooling of IPD is conducted in the framework of a prospective meta-analysis and that the conclusion of such analyses must be made, taking into account the inevitable selection bias.^56^

Our results also highlight the importance of clear reporting of the study and participant numbers contributing to different stages of the IPD meta-analysis, with an adequate investigation of the reasons for lack of data and discussion of the potential for data availability bias. The total number of eligible participants and the total number of participants’ data requested was unclear in 34% of published IPD meta-analyses; in 58% of the IPD meta-analyses that failed to retrieve 100% of eligible IPD, no specific reasons were provided for the unavailability of data, making interpretation of IPD meta-analyses results and conclusions in the presence of potential availability bias difficult; and in a quarter of IPD meta-analyses unable to retrieve 100% of IPD, there was a complete lack of discussion or acknowledgment of availability bias. A systematic investigation of the impact of availability bias on the conclusions of IPD meta-analyses was outside the scope of this review and is likely specific to the clinical context in question. Furthermore, our own experiences of requesting IPD show that this issue is not restricted to the reporting of IPD meta-analyses but that it also exists at the study request level; IPD from three out of 35 studies were not available to us, with no further reason stated (web appendix 5). Despite this, further efforts are recommended by researchers conducting an IPD meta-analysis to thoroughly investigate and report the impact of data availability.^15^

We hope that the uptake of PRISMA (Preferred Reporting Items for Systematic Reviews and Meta-Analyses) guidelines for the conduct and reporting of IPD meta-analyses,^57^ in addition to guidance on the use of IPD meta-analysis to synthesise the results of randomised controlled trials,^58^ will lead to improved conduct and reporting in IPD meta-analyses, particularly regarding transparent reporting of the number of eligible studies and participants, how much data were requested and obtained, with clear reasons for non-availability of IPD, preferably with a flow diagram, and data collection methods. Discussion of the limitations and impact on conclusions of missing IPD is essential.

### Conclusions

Individual participant data (IPD) meta-analyses are resource demanding, time consuming, and methodologically challenging, but when conducted well,^58^ ideally following a registered protocol^59^ and adhering to the PRISMA-IPD guidance,^57^ can provide more detailed and potentially more reliable results than a meta-analysis of aggregate data. Meta-analysts must consider the appropriateness of an IPD analysis and document the potential biases introduced by missing such data. Only one in four published IPD meta-analyses have had access to all IPD; we hope that this proportion will increase in future years with the growing awareness of data sharing and transparency in the pharmaceutical industry and beyond.^22^ ^23^ ^24^ ^26^ ^27^ ^28^ However, the research community must ensure that procedures to access IPD do not become over-burdensome, over-costly, and prohibitive, and that common sense and responsible risk proportionate approaches should be used.^23^ ^27^

**What is already known on this topic**

Individual participant data (IPD) meta-analyses are widely regarded as the “gold standard” approach to the synthesis of data from clinical research studies but is susceptible to bias if only a proportion of IPD are available for analysis and the IPD are not representative of the patient population
IPD meta-analyses are often poorly reported in terms of proportion of IPD retrieved and reasons for non-availability of IPD
Recent years have seen a shift in attitudes and awareness towards data sharing

**What this study adds**

This systematic review of 760 IPD meta-analyses published between 1987 and 2015 showed that a quarter retrieved 100% of the eligible IPD and half retrieved less than 80% of the eligible IPD
Despite the substantial drive towards improving access to clinical research data, the IPD retrieval rate across 760 published IPD meta-analyses has not improved over time
Higher IPD retrieval rates were associated with IPD meta-analyses that only included randomised trials, had fewer eligible participants, used an authorship policy, and were conducted outside of a Cochrane review

## Web Extra

Extra material supplied by the author

**Appendix 1** **:** Details of search strategies

**Appendix 2** **:** Data extraction form

**Appendix 3** **:** Statistical analysis

**Appendix 4** **:** Reference details of 1278 eligible articles describing 1280 IPD meta-analyses

**Appendix 5** **:** Outcome of IPD requests

**Appendix figure:** Characteristics of systematic IPD meta-analyses and proportion of IPD provided

## Notes

Contributors: SJN, AGM, and CTS conceived and designed the research question. SJN, BD, SR, and LW extracted data. SJN analysed data. SJN and CTS interpreted the results. SJN wrote the manuscript under the supervision of AGM and CTS. All authors had full access to all of the data (including statistical reports and tables) in the study and can take responsibility for the integrity of the data and the accuracy of the data analysis. SJN is the guarantor.

Ethical approval: Not required.

Data sharing: Data underlying tables and figures are available on request from the corresponding author.

Transparency: The lead author (SJN) affirms that the manuscript is an honest, accurate, and transparent account of the study being reported; that no important aspects of the study have been omitted; and that any discrepancies from the study as planned (and, if relevant, registered) have been explained.
