# Supplementary material for: Exploring changes over time and characteristics associated with data retrieval across individual participant data meta-analyses: systematic review
Source: BMJ. 2017 Apr 5;357:j1390. doi: 10.1136/bmj.j1390 (PMC5733815; doi:10.1136/bmj.j1390)
Supplement: Supplementary file 1 — Appendix 1 : Details of search strategies [file nevs036543.ww1.pdf]

Appendix 1: Search Strategies for identification of individual participant data meta-analyses

All databases were searched from June 2005 (end date of the Riley *et a*l 2007[1] search) up to June 2014 initially and all systematic searches were updated in August 2015.

1 Epilepsy Specialized Register (CRS) *(search dates: 12/06/2014 and 17/08/2015)*

#1 ("individual patient*" ADJ6 (data OR report* OR outcome* OR level*)):TI,AB
#2 ("individual participant data" OR ipd):TI,AB
#3 ("individual subject*" ADJ6 (data OR report* OR outcome* OR level*)):TI,AB
#4 ("raw patient*" ADJ6 (data OR report* OR outcome* OR level*)):TI,AB
#5 ("raw subject*" ADJ6 (data OR report* OR outcome* OR level*)):TI,AB
#6 (idiopathic OR "immediate pigment darkening" OR "intermittent peritoneal dialysis" OR "invasive pneumococcal disease" OR "indirect photometric detection" OR "interaural phase disparity"):TI,AB
#7 #1 OR #2 OR #3 OR #4 OR #5
#8 (#7 NOT #6) AND >2003:YR

2 Cochrane Central Register of Controlled Trials (CENTRAL) via CRSO *(search dates: 12/06/2014 and 17/08/2015)*

#1 ("individual patient*" ADJ6 (data OR report* OR outcome* OR level*)):TI,AB
#2 ("individual participant data" OR ipd):TI,AB
#3 ("individual subject*" ADJ6 (data OR report* OR outcome* OR level*)):TI,AB
#4 ("raw patient*" ADJ6 (data OR report* OR outcome* OR level*)):TI,AB
#5 ("raw subject*" ADJ6 (data OR report* OR outcome* OR level*)):TI,AB
#6 (idiopathic OR "immediate pigment darkening" OR "intermittent peritoneal dialysis" OR "invasive pneumococcal disease" OR "indirect photometric detection" OR "interaural phase disparity"):TI,AB
#7 #1 OR #2 OR #3 OR #4 OR #5
#8 #7 NOT #6
#9 2004 TO 2014:YR NOT INMEDLINE
#10 #8 AND #9

3 MEDLINE (Ovid) *(search dates: 10/06/2014 and 17/08/2015)*

1. (individual patient$ adj6 data).ti,ab.
2. (individual patient$ adj6 report$).ti,ab.
3. (individual patient$ adj6 outcome$).ti,ab.
4. (individual patient$ adj6 level$).ti,ab.
5. individual participant data.ti,ab.

6. ipd.ti,ab.
7. (individual subject$ adj6 data).ti,ab.
8. (individual subject$ adj6 report$).ti,ab.
9. (individual subject$ adj6 outcome$).ti,ab.
10. (individual subject$ adj6 level$).ti,ab.
11. (raw patient$ adj6 data).ti,ab.
12. (raw patient$ adj6 report$).ti,ab.
13. (raw patient$ adj6 outcome$).ti,ab.
14. (raw patient$ adj6 level$).ti,ab.
15. (raw subject$ adj6 data).ti,ab.
16. (raw subject$ adj6 report$).ti,ab.
17. (raw subject$ adj6 outcome$).ti,ab.
18. (raw subject$ adj6 level$).ti,ab.
19. idiopathic.ti,ab.
20. immediate pigment darkening.ti,ab.
21. intermittent peritoneal dialysis.ti,ab.
22. invasive pneumococcal disease.ti,ab.
23. indirect photometric detection.ti,ab.
24. interaural phase disparity.ti,ab.
25. or/1-18
26. or/19-24
27. 25 not 26
28. limit 27 to ed=20050601-20140610

4. SCOPUS *(search dates: 10/06/2014 and 18/08/2015)*

(((((TITLE-ABS-KEY("individual patient*" PRE/6 data)) OR (TITLE-ABS-KEY("individual patient*" PRE/6 report*)) OR (TITLE-ABS-KEY("individual patient*" PRE/6 outcome*)) OR (TITLE-ABS-KEY("individual patient*" PRE/6 level*)) OR (TITLE-ABS-KEY("individual participant data")) OR (TITLE-ABS-KEY(ipd)) OR (TITLE-ABS-KEY("individual subject*" PRE/6 data)) OR (TITLE-ABS-KEY("individual subject*" PRE/6 report*)) OR (TITLE-ABS-KEY("individual subject*" PRE/6 outcome*))) OR ((TITLE-ABS-KEY("individual subject*" PRE/6 level*)) OR (TITLE-ABS-KEY("raw patient*" PRE/6 data)) OR (TITLE-ABS-KEY("raw patient*" PRE/6 report*)) OR (TITLE-ABS-KEY("raw patient*" PRE/6 outcome*)) OR (TITLE-ABS-KEY("raw patient*" PRE/6 level*)) OR (TITLE-ABS-KEY("raw subject*" PRE/6 data))) OR ((TITLE-ABS-KEY("raw subject*" PRE/6 report*)) OR (TITLE-ABS-KEY("raw subject*" PRE/6 outcome*)) OR (TITLE-ABS-KEY("raw subject*" PRE/6 level*)))) AND NOT ((TITLE-ABS-KEY(idiopathic)) OR (TITLE-ABS-KEY("immediate pigment darkening")) OR (TITLE-ABS-KEY("intermittent peritoneal dialysis")) OR (TITLE-ABS-KEY("invasive pneumococcal disease")) OR (TITLE-ABS-KEY("indirect photometric detection")) OR (TITLE-ABS-KEY("interaural phase disparity")))) AND PUBYEAR > 2004) AND NOT (INDEX(medline)) AND (LIMIT-TO(SUBJAREA,"MEDI") OR LIMIT-TO(SUBJAREA,"BIOC") OR LIMIT-TO(SUBJAREA,"NEUR") OR LIMIT-TO(SUBJAREA,"PHAR") OR LIMIT-TO(SUBJAREA,"IMMU") OR LIMIT-TO(SUBJAREA,"NURS") OR LIMIT-TO(SUBJAREA,"HEAL") OR LIMIT-TO(SUBJAREA,"PSYC") OR LIMIT-TO(SUBJAREA,"DENT"))

5. CINAHL Plus and PsycINFO (EBSCOhost) *(search dates: 10/06/2014 and 18/08/2015)*

S9	S7 NOT S6<br>Published: 20040601-
S8	S7 NOT S6
S7	S1 OR S2 OR S3 OR S4 OR S5
S6	TX (idiopathic OR "immediate pigment darkening") OR TX "intermittent peritoneal dialysis" OR TX "invasive pneumococcal disease" OR TX "indirect photometric detection" OR TX "interaural phase disparity"
S5	"raw subject*" W6 (data OR report* OR outcome* OR level*)
S4	"raw patient*" W6 (data OR report* OR outcome* OR level*)
S3	"individual subject*" W6 (data OR report* OR outcome* OR level*)
S2	"individual participant data" OR TX ipd
S1	"individual patient*" W6 (data OR report* OR outcome* OR level*)

6. Web of Science: Core Collection 1900- and BIOSIS Previews (Biological Abstracts) *(search dates: 10/06/2014 and 18/08/2015)*

#12	#5 OR #4 OR #3 OR #2 OR #1<br>Refined by: **PUBLICATION YEARS:** (2013 OR 2004 OR 2012 OR 2011 OR 2010 OR 2009 OR 2008 OR 2007 OR 2005 OR 2006 OR 2014) AND **Databases:** (WOS OR BIOSIS) AND **[excluding]:Databases:** (MEDLINE) AND **RESEARCH DOMAINS:** (SCIENCE TECHNOLOGY) AND **RESEARCH AREAS:** (BEHAVIORAL SCIENCES OR OTORHINOLARYNGOLOGY OR PHARMACOLOGY PHARMACY OR PSYCHIATRY OR TRANSPLANTATION OR ONCOLOGY OR RHEUMATOLOGY OR ALLERGY OR CARDIOVASCULAR SYSTEM CARDIOLOGY OR ANESTHESIOLOGY OR PSYCHOLOGY OR DERMATOLOGY OR GENERAL INTERNAL MEDICINE OR REHABILITATION OR HEMATOLOGY OR ORTHOPEDICS OR REPRODUCTIVE BIOLOGY OR IMMUNOLOGY OR CRITICAL CARE MEDICINE OR NEUROSCIENCES NEUROLOGY OR OPHTHALMOLOGY OR NUTRITION DIETETICS OR TROPICAL MEDICINE OR PUBLIC ENVIRONMENTAL OCCUPATIONAL HEALTH OR GASTROENTEROLOGY HEPATOLOGY OR DENTISTRY ORAL SURGERY MEDICINE OR OBSTETRICS GYNECOLOGY OR GERIATRICS GERONTOLOGY OR PATHOLOGY OR RADIOLOGY NUCLEAR MEDICINE MEDICAL IMAGING OR PARASITOLOGY OR SUBSTANCE ABUSE OR RESEARCH EXPERIMENTAL MEDICINE OR SURGERY OR RESPIRATORY SYSTEM OR DEVELOPMENTAL BIOLOGY OR HEALTH CARE SCIENCES SERVICES OR PHYSIOLOGY OR UROLOGY NEPHROLOGY OR VIROLOGY OR ENDOCRINOLOGY METABOLISM OR PEDIATRICS OR INFECTIOUS DISEASES OR NURSING OR MICROBIOLOGY OR TOXICOLOGY) AND **[excluding]:DOCUMENT TYPES:** (PATENT OR EDITORIAL)<br>*DocType=All document types; Language=All languages;*
#11	#5 OR #4 OR #3 OR #2 OR #1<br>Refined by: **PUBLICATION YEARS:** (2013 OR 2004 OR 2012 OR 2011 OR 2010 OR 2009 OR 2008 OR 2007 OR 2005 OR 2006 OR 2014) AND **Databases:** (WOS OR BIOSIS) AND **[excluding]:Databases:** (MEDLINE) AND **RESEARCH DOMAINS:** (SCIENCE TECHNOLOGY) AND **RESEARCH AREAS:** (BEHAVIORAL SCIENCES OR OTORHINOLARYNGOLOGY OR PHARMACOLOGY PHARMACY OR PSYCHIATRY OR TRANSPLANTATION OR ONCOLOGY OR RHEUMATOLOGY OR ALLERGY OR CARDIOVASCULAR SYSTEM CARDIOLOGY OR ANESTHESIOLOGY OR PSYCHOLOGY OR DERMATOLOGY OR GENERAL INTERNAL MEDICINE OR REHABILITATION OR HEMATOLOGY OR ORTHOPEDICS OR REPRODUCTIVE BIOLOGY OR IMMUNOLOGY OR CRITICAL CARE MEDICINE OR NEUROSCIENCES NEUROLOGY OR OPHTHALMOLOGY OR NUTRITION DIETETICS OR TROPICAL MEDICINE OR PUBLIC ENVIRONMENTAL OCCUPATIONAL HEALTH OR GASTROENTEROLOGY HEPATOLOGY OR DENTISTRY ORAL SURGERY MEDICINE OR OBSTETRICS

	GYNECOLOGY OR GERIATRICS GERONTOLOGY OR PATHOLOGY OR RADIOLOGY NUCLEAR MEDICINE MEDICAL IMAGING OR PARASITOLOGY OR SUBSTANCE ABUSE OR RESEARCH EXPERIMENTAL MEDICINE OR SURGERY OR RESPIRATORY SYSTEM OR DEVELOPMENTAL BIOLOGY OR HEALTH CARE SCIENCES SERVICES OR PHYSIOLOGY OR UROLOGY NEPHROLOGY OR VIROLOGY OR ENDOCRINOLOGY METABOLISM OR PEDIATRICS OR INFECTIOUS DISEASES OR NURSING OR MICROBIOLOGY OR TOXICOLOGY)<br>*DocType=All document types; Language=All languages;*
#10	#5 OR #4 OR #3 OR #2 OR #1<br>Refined by: **PUBLICATION YEARS:** (2013 OR 2004 OR 2012 OR 2011 OR 2010 OR 2009 OR 2008 OR 2007 OR 2005 OR 2006 OR 2014) AND **Databases:** (WOS OR BIOSIS) AND **[excluding]:Databases:** (MEDLINE) AND **RESEARCH DOMAINS:** (SCIENCE TECHNOLOGY)<br>*DocType=All document types; Language=All languages;*
#9	#5 OR #4 OR #3 OR #2 OR #1<br>Refined by: **PUBLICATION YEARS:** (2013 OR 2004 OR 2012 OR 2011 OR 2010 OR 2009 OR 2008 OR 2007 OR 2005 OR 2006 OR 2014) AND **Databases:** (WOS OR BIOSIS) AND **[excluding]:Databases:** (MEDLINE)<br>*DocType=All document types; Language=All languages;*
#8	#5 OR #4 OR #3 OR #2 OR #1<br>Refined by: **PUBLICATION YEARS:** (2013 OR 2004 OR 2012 OR 2011 OR 2010 OR 2009 OR 2008 OR 2007 OR 2005 OR 2006 OR 2014) AND **Databases:** (WOS OR BIOSIS)<br>*DocType=All document types; Language=All languages;*
#7	#5 OR #4 OR #3 OR #2 OR #1<br>Refined by: **PUBLICATION YEARS:** (2013 OR 2004 OR 2012 OR 2011 OR 2010 OR 2009 OR 2008 OR 2007 OR 2005 OR 2006 OR 2014)<br>*DocType=All document types; Language=All languages;*
#6	#5 OR #4 OR #3 OR #2 OR #1<br>*DocType=All document types; Language=All languages;*
#5	**TOPIC:** ("raw subject*" NEAR/6 (data OR report* OR outcome* OR level*)) *NOT* **TOPIC:** (idiopathic OR "immediate pigment darkening" OR "intermittent peritoneal dialysis" OR "invasive pneumococcal disease" OR "indirect photometric detection" OR "interaural phase disparity")<br>*DocType=All document types; Language=All languages;*
#4	**TOPIC:** ("raw patient*" NEAR/6 (data OR report* OR outcome* OR level*)) *NOT* **TOPIC:** (idiopathic OR "immediate pigment darkening" OR "intermittent peritoneal dialysis" OR "invasive pneumococcal disease" OR "indirect photometric detection" OR "interaural phase disparity")<br>*DocType=All document types; Language=All languages;*
#3	**TOPIC:** ("individual subject*" NEAR/6 (data OR report* OR outcome* OR level*)) *NOT* **TOPIC:** (idiopathic OR "immediate pigment darkening" OR "intermittent peritoneal dialysis" OR "invasive pneumococcal disease" OR "indirect photometric detection" OR "interaural phase disparity")<br>*DocType=All document types; Language=All languages;*
#2	**TOPIC:** ("individual participant data" OR ipd) *NOT* **TOPIC:** (idiopathic OR "immediate pigment darkening" OR "intermittent peritoneal dialysis" OR "invasive pneumococcal disease" OR "indirect photometric detection" OR "interaural phase disparity")<br>*DocType=All document types; Language=All languages;*
#1	**TOPIC:** ("individual patient*" NEAR/6 (data OR report* OR outcome* OR level*)) *NOT* **TOPIC:** (idiopathic OR "immediate pigment darkening" OR "intermittent peritoneal dialysis" OR "invasive pneumococcal disease" OR "indirect photometric detection" OR "interaural phase disparity")<br>*DocType=All document types; Language=All languages;*

1. Riley RD, Simmonds MC, Look MP. Evidence synthesis combining individual patient data and aggregate data: a systematic review identified current practice and possible methods. J Clin Epidemiol 2007;**60**(5):431-9.
