# Supplementary material for: Exploring changes over time and characteristics associated with data retrieval across individual participant data meta-analyses: systematic review
Source: BMJ. 2017 Apr 5;357:j1390. doi: 10.1136/bmj.j1390 (PMC5733815; doi:10.1136/bmj.j1390)
Supplement: Supplementary file 2 — Appendix 2 : Data extraction form [file nevs036543.ww2.pdf]

Individual Participant Data Meta-Analyses: Data Extraction Form

Date of Extraction:

Name of data extractor:

Meta-analysis First Author:

Meta-analysis Year:

Meta-analysis Title:

Journal or Source:

Authorship policy (individual authorship, collaborative group, none):

Source of funding:

Clinical area (lung cancer, breast cancer, epilepsy, diabetes etc.):

Design of studies included (Randomised / Non-randomised/ both / Other (diagnostic test accuracy etc.):

Type of studies included (Drug / Device / Observational / Other (diagnostic test accuracy etc.):

Type of pooled analysis: Systematic search performed or existing database of studies pooled/ collaboration?

Number of studies eligible for meta-analysis:

Number of participants in all eligible studies:

Year range of eligible studies:

Number of studies providing IPD:

Number of participants IPD is provided for:

Number of studies providing aggregate data (AD):

Number of participants AD is provided for:

Number of studies excluded due to no IPD or AD available:

Number of patients excluded due to no IPD or AD available:

Year range of studies IPD is not available for:

Any reported reasons that IPD was not provided (data no longer available, authors unwilling to collaborate)?

Were any adjustments/ sensitivity analyses performed to account for missing IPD? Or do meta-analysis authors note the limitation of missing IPD?

Additional notes:

Footnotes:

1. Reasons for IPD not being provided and sensitivity analyses recorded as free text and later classified into broad categories.
2. Source of funding recorded as free text and later classified as Commercial, Non-Commercial, Mixed (Commercial and Non-Commercial), No funding, Not stated.
3. Clinical area was also recorded as free text and later classified in broad categories based on the clinical areas covered by the review groups of the Cochrane Collaboration
