# Supplementary material for: Exploring changes over time and characteristics associated with data retrieval across individual participant data meta-analyses: systematic review
Source: BMJ. 2017 Apr 5;357:j1390. doi: 10.1136/bmj.j1390 (PMC5733815; doi:10.1136/bmj.j1390)
Supplement: Supplementary file 3 — Appendix 3 : Statistical analysis [file nevs036543.ww3.pdf]

Appendix 3: Statistical Analysis

1. Characteristics associated with a high success rate of IPD retrieval

Proportion of IPD retrieved (dependent variable of interest) was highly skewed, despite attempts at transformation, as few IPDMA retrieved a very small proportion of data. It was therefore deemed most appropriate to dichotomise this variable to:

a) Complete IPD retrieval rate (100% compared to less than 100% or unknown proportion of IPD provided)

b) High IPD retrieval rate (at least 80% compared to less than 80% or unknown proportion of IPD provided)

Dichotomisation was chosen to allow comparison with retrieval rates in previous reviews.[1-4] A sensitivity analysis using fractional logistic regression was also performed with proportion of IPD retrieved (dependent variable) expressed as a fraction between 0 and 1;[5] results are presented below.

Multivariable logistic regression was performed to examine associations between individual participant data meta-analyses (IPDMA) characteristics and a high or complete retrieval rate of IPD.

A total of 503 IPDMA were included in this analysis for which we could calculate the proportion of IPD retrieved (i.e. the number of participant eligible for analysis and the number of participants data was provided for was reported).

The following variables were included in the model and results for all variables included in the model are presented regardless of statistical significance; no model selection techniques were used:

- Age of publication (calculated as years before 2016, log transformed due to skew)
- Number of participants eligible for inclusion in IPDMA (log transformed due to skew)
- Inclusion of randomised studies only in IPDMA compared to IPDMA including non-randomised studies, diagnostic test accuracy studies or a combination of randomised and non-randomised studies
- IPDMA performed as a Cochrane Review compared to non-Cochrane IPDMA
- IPDMA with an authorship policy (individual authorship or collaborative group) compared to no authorship policy
- IPDMA with a commercial source of funding (pharmaceutical or manufacturer) compared to non-commercial sources of funding only, no funding or no information regarding funding provided.

Results of the multivariable logistic regression (primary analysis) are presented in Table 2.

Sensitivity analyses and additional analyses

1. Primary analysis was a multivariable analysis, therefore all odds ratios presented in Table 2 are adjusted odds ratios. Univariate analysis was performed for each variable of interest and unadjusted odds ratios are presented below:

IPD MA	100% of IPD retrieved compared to	At least 80% of IPD retrieved

Characteristic	less than 100% of IPD			compared to less than 80% of IPD		
	Odds Ratio	95% Confidence Interval	P value	Odds Ratio	95% Confidence Interval	P value
Age of publication*	0.918	0.748 to 1.126	0.411	1.102	0.897 to 1.355	0.355
Number of eligible participants*	0.701	0.625 to 0.787	<0.001	0.872	0.789 to 0.965	0.008
Includes randomised studies only	1.113	0.768 to 1.613	0.571	2.517	1.729 to 3.666	<0.001
Cochrane IPD-MA	0.365	0.179 to 0.746	0.006	0.471	0.264 to 0.839	0.011
Authorship Policy	1.225	0.840 to 1.786	0.292	3.123	2.132 to 4.577	<0.001
Commercial source of funding	1.360	0.834 to 2.218	0.218	1.606	0.938 to 2.750	0.084

* Log transformation applied due to skewed distribution of data

Results of this analysis are numerically similar to those of multivariable (adjusted) analysis presented in Table 2; the only difference in conclusions is that unadjusted analysis shows no association between complete IPD retrieval rate and an authorship policy (association between authorship policy and high retrieval rate is maintained in adjusted and unadjusted analysis).

2. A sensitivity analysis was performed to further examine association of authorship policy on IPD retrieval. Multivariable logistic regression was repeated with authorship policy redefined as no authorship policy (reference), individual authorship or collaborative group.

IPD MA Characteristic	100% of IPD retrieved compared to less than 100% of IPD			At least 80% of IPD retrieved compared to less than 80% of IPD		
	Odds Ratio	95% Confidence Interval	P value	Odds Ratio	95% Confidence Interval	P value
Age of publication*	1.137	0.923 to 1.402	0.228	1.155	0.939 to 1.420	0.173
Number of eligible participants*	0.843	0.791 to 0.898	<0.001	0.889	0.838 to 0.943	<0.001
Includes randomised studies only	1.491	0.957 to 2.322	0.078	2.748	1.761 to 4.288	<0.001
Cochrane IPD-MA	0.450	0.208 to 0.973	0.042	0.432	0.221 to 0.844	0.014
No Authorship Policy	Ref.	Ref.	Ref.	Ref.	Ref.	Ref.
Individual authorship	2.795	1.729 to 4.519	<0.001	3.583	2.173 to 5.908	<0.001
Collaborative Group	0.761	0.442 to 1.309	0.324	3.130	1.867 to 5.249	<0.001
Commercial source of funding	1.623	0.933 to 2.824	0.086	1.061	0.576 to 1.953	0.849

* Log transformation applied due to skewed distribution of data

Results of this analysis show that odds of retrieving at least 80% of IPD were significantly increased when either individual authorship or collaborative authorship policies were used but odds of retrieving at 100% of IPD were significantly increased only when an individual authorship policy was used. Other numerical results were similar to those in Table 2 and results unchanged.

3. Type of study (drug or device (interventional), non-drug (interventional), diagnostic test accuracy or epidemiological study) was not included in the model due to correlation between

this variable and type of study (interventional studies were significantly more likely to be randomised, chi-squared p<0.001) and source of funding (drug or device studies were significantly more likely to be commercially funded, chi- squared p<0.001).

Sensitivity analysis was conducted adding an additional variable to this model of IPDMA of drug or device studies compared to non-drug or device interventions, diagnostic test accuracy studies or epidemiological studies added to the model.

IPD MA Characteristic	100% of IPD retrieved compared to less than 100% of IPD			At least 80% of IPD retrieved compared to less than 80% of IPD		
	Odds Ratio	95% Confidence Interval	P value	Odds Ratio	95% Confidence Interval	P value
Age of publication*	1.068	0.874 to 1.306	0.520	1.138	0.924 to 1.402	0.224
Number of eligible participants*	0.845	0.794 to 0.898	<0.001	0.881	0.830 to 0.936	<0.001
Includes randomised studies only	1.220	0.756 to 1.971	0.415	2.253	1.372 to 3.670	0.001
Drug or device	1.374	0.887 to 2.130	0.155	1.492	0.936 to 2.379	0.093
Cochrane IPD-MA	0.403	0.189 to 0.862	0.019	0.429	0.219 to 0.841	0.014
Authorship Policy	1.710	1.101 to 2.658	0.017	3.491	2.252 to 5.413	<0.001
Commercial source of funding	1.207	0.707 to 2.059	0.491	0.948	0.511 to 1.757	0.865

* Log transformation applied due to skewed distribution of data

Results showed that this characteristic was not statistically significant, other numerical results were similar to those in Table 2 and conclusions were unchanged.

4. A sensitivity analysis was conducted excluding 128 IPD-MAs with no information regarding funding.

IPD MA Characteristic	100% of IPD retrieved compared to less than 100% of IPD			At least 80% of IPD retrieved compared to less than 80% of IPD		
	Odds Ratio	95% Confidence Interval	P value	Odds Ratio	95% Confidence Interval	P value
Age of publication*	1.029	0.804 to 1.318	0.818	1.094	0.848 to 1.411	0.490
Number of eligible participants*	0.847	0.789 to 0.909	<0.001	0.891	0.833 to 0.952	0.001
Includes randomised studies only	1.318	0.783 to 2.217	0.298	3.013	1.779 to 5.103	<0.001
Cochrane IPD-MA	0.419	0.180 to 0.977	0.044	0.392	0.188 to 0.818	0.013
Authorship Policy	1.726	1.014 to 2.936	0.044	3.583	2.154 to 5.961	<0.001
Commercial source of funding	1.544	0.885 to 2.694	0.126	1.003	0.529 to 1.902	0.992

* Log transformation applied due to skewed distribution of data

Numerical results were similar to those in Table 2 and conclusions were unchanged.

5. A sensitivity analysis was conducted including all 760 IPDMA, assuming the following scenarios for the 257 IPDMA for which the proportion of IPD retrieved could not be calculated:

a. Less than 80% of IPD was retrieved
b. 80% or more IPD was retrieved
c. 100% of IPD was retrieved

The multivariable regression was run under each scenario including the above variables except for number of eligible participants (not available for the 257 additional IPDMA).

a) Assuming less than 80% of IPD was retrieved for 257 IPDMA where proportion of IPD retrieved could not be calculated

**IPD MA Characteristic**	**100% of IPD retrieved compared to less than 100% of IPD**			**At least 80% of IPD retrieved compared to less than 80% of IPD**		
	**Odds Ratio**	**95% Confidence Interval**	**P value**	**Odds Ratio**	**95% Confidence Interval**	**P value**
Age of publication*	0.641	0.554 to 0.739	<0.001	0.713	0.624 to 0.816	<0.001
Includes randomised studies only	1.667	1.160 to 2.393	0.006	2.906	2.076 to 4.067	<0.001
Cochrane IPD-MA	0.334	0.161 to 0.701	0.004	0.489	0.278 to 0.861	0.013
Authorship Policy	0.505	0.372 to 0.685	<0.001	0.842	0.635 to 1.115	0.230
Commercial source of funding	1.102	0.687 to 1.767	0.687	0.994	0.642 to 1.541	0.980

* Log transformation applied due to skewed distribution of data

b) Assuming 80% or more of IPD was retrieved for 257 IPDMA where proportion of IPD retrieved could not be calculated

**IPD MA Characteristic**	**100% of IPD retrieved compared to less than 100% of IPD**			**At least 80% of IPD retrieved compared to less than 80% of IPD**		
	**Odds Ratio**	**95% Confidence Interval**	**P value**	**Odds Ratio**	**95% Confidence Interval**	**P value**
Age of publication*	0.641	0.554 to 0.739	<0.001	1.206	1.046 to 1.392	0.010
Includes randomised studies only	1.667	1.160 to 2.393	0.006	1.479	0.999 to 2.190	0.051
Cochrane IPD-MA	0.334	0.161 to 0.701	0.004	0.428	0.233 to 0.784	0.006
Authorship Policy	0.505	0.372 to 0.685	<0.001	3.222	2.340 to 4.439	<0.001
Commercial source of funding	1.102	0.687 to 1.767	0.687	1.002	0.577 to 1.743	0.993

* Log transformation applied due to skewed distribution of data

c) Assuming 100% of IPD was retrieved for 257 IPDMA where proportion of IPD retrieved could not be calculated

**IPD MA Characteristic**	**100% of IPD retrieved compared to less than 100% of IPD**			**At least 80% of IPD retrieved compared to less than 80% of IPD**		
	**Odds Ratio**	**95% Confidence Interval**	**P value**	**Odds Ratio**	**95% Confidence Interval**	**P value**
Age of publication*	1.109	0.975 to 1.392	0.114	1.206	1.046 to 1.392	0.010
Includes randomised studies only	0.682	0.492 to 0.947	0.022	1.479	0.999 to 2.190	0.051

Cochrane IPD-MA	0.477	0.269 to 0.846	0.011	0.428	0.233 to 0.784	0.006
Authorship Policy	1.762	1.331 to 2.332	<0.001	3.222	2.340 to 4.439	<0.001
Commercial source of funding	1.092	0.704 to 1.693	0.993	1.002	0.577 to 1.743	0.993

* Log transformation applied due to skewed distribution of data

Results of these sensitivity analyses are varied compared to those reported in Table 2 – for example scenario a) and scenario b) contradict Table 2 and suggest that the odds of complete or high IPD retrieval rate are significantly higher in IPDMA without an authorship policy. These sensitivity analyses highlight the importance of a reporting the proportion of IPD retrieved in IPDMA.

An additional analysis was also performed to examine characteristics of the 257 IPDMA where proportion of IPD retrieved could not be calculated compared to the 503 IPDMA were proportion of IPDMA could be calculated

**IPD MA Characteristic****	**Proportion of IPD retrieved unknown compared to proportion of IPD retrieved known**		
	**Odds Ratio**	**95% Confidence Interval**	**P value**
Age of publication (log scale)*	0.869	0.761 to 0.992	0.039
Includes randomised studies only	0.361	0.256 to 0.508	<0.001
Cochrane IPD-MA	0.658	0.332 to 1.303	0.231
Authorship Policy	1.397	1.043 to 1.869	0.025
Commercial source of funding	0.849	0.523 to 1.379	0.509

* Log transformation applied due to skewed distribution of data

**Number of eligible participants not available for 257 IPDMA, not included in analysis

Results of this additional analysis indicate that the odds of the proportion of IPD retrieved being reported are significantly higher in more recently published IPDMA, IPDMA including IPDMA only and IPDMA without an authorship. There was no association between publication as a Cochrane IPDMA and the source of funding on the reporting of the proportion of IPD-MA

6. Fractional logistic regression was also performed with proportion of IPD retrieved as the dependent variable

**IPDMA Characteristic**	**Odds Ratio**	**95% Confidence Interval****	**P value**
Age of publication*	1.339	1.152 to 1.555	<0.001
Number of eligible participants*	0.998	0.946 to 1.032	0.591
Includes randomised studies only	2.432	1.775 to 3.333	<0.001
Cochrane IPD-MA	0.446	0.288 to 0.691	<0.001
Authorship Policy	2.511	1.835 to 3.436	<0.001
Commercial source of funding	0.871	0.544 to 1.394	0.565

* Log transformation applied due to skewed distribution of data

**Calculated with robust standard errors.

Results of this analysis indicate that odds of retrieving a higher proportion of IPD are significantly associated with older IPDMA, IPDMA including only randomised studies, non-Cochrane IPDMA and IPDMA with an authorship policy. There was no association between the number of eligible participants and source of funding on the proportion of IPD retrieved.

7. Multivariable logistic regression was also performed to examine associations between the IPDMA characteristics defined above and the proportion of study data retrieved.

A total of 744 IPDMA were included in this analysis for which we could calculate the proportion of study data retrieved (i.e. the number of studies eligible for analysis and the number of studies data was provided for was reported).

IPD MA Characteristic	100% of study data retrieved compared to less than 100% of study data			At least 80% of study data retrieved compared to less than 80% of study data		
	**Odds Ratio**	**95% Confidence Interval**	**P value**	**Odds Ratio**	**95% Confidence Interval**	**P value**
Age of publication*	1.172	0.961 to 1.431	0.116	1.235	1.050 to 1.454	0.011
Number of eligible studies*	0.498	0.428 to 0.576	<0.001	0.681	0.610 to 0.759	<0.001
Includes randomised studies only	1.555	1.050 to 2.304	0.028	1.301	0.936 to 1.807	0.117
Cochrane IPD-MA	0.441	0.207 to 0.937	0.033	0.664	0.373 to 1.181	0.163
Authorship Policy	1.078	0.739 to 1.573	0.695	1.851	1.355 to 2.529	<0.001
Commercial source of funding	1.339	0.819 to 2.187	0.244	1.227	0.781 to 1.927	0.375

* Log transformation applied due to skewed distribution of data

Results of this sensitivity analysis are mostly similar to those reported in Table 2, however these results suggest that the odds of retrieving at least 80% of study data are significantly associated with older IPDMA; suggesting that IPD retrieval rate on a study level has got worse over time.

References

1. Riley RD, Simmonds MC, Look MP. Evidence synthesis combining individual patient data and aggregate data: a systematic review identified current practice and possible methods. J Clin Epidemiol 2007;**60**(5):431-9.
2. Simmonds M, Stewart G, Stewart L. A decade of individual participant data meta-analyses: A review of current practice. Contemp Clin Trials 2015;**45**(Pt A):76-83.
3. Simmonds MC, Higgins JP, Stewart LA, et al. Meta-analysis of individual patient data from randomized trials: a review of methods used in practice. Clin Trials 2005;**2**(3):209-17.
4. Huang Y, Mao C, Yuan J, et al. Distribution and epidemiological characteristics of published individual patient data meta-analyses. PLoS One 2014;**9**(6):e100151.
5. Papke LE, Wooldridge J. Econometric methods for fractional response variables with an application to 401 (k) plan participation rates: National Bureau of Economic Research Cambridge, Mass., USA, 1993.
