# Supplementary material for: Exploring changes over time and characteristics associated with data retrieval across individual participant data meta-analyses: systematic review
Source: BMJ. 2017 Apr 5;357:j1390. doi: 10.1136/bmj.j1390 (PMC5733815; doi:10.1136/bmj.j1390)
Supplement: Supplementary file 4 — Appendix 4 : Reference details of 1278 eligible articles describing 1280 IPD meta-analyses [file nevs036543.ww4.pdf]

1. Aabo K, Adams M, Adnitt P, et al. Chemotherapy in advanced ovarian cancer: four systematic meta-analyses of individual patient data from 37 randomized trials. Advanced Ovarian Cancer Trialists' Group. Br J Cancer 1998;**78**(11):1479-87.
2. Abdulla J, Pogue J, Abildstrom SZ, et al. Effect of angiotensin-converting enzyme inhibition on functional class in patients with left ventricular systolic dysfunction--a meta-analysis. Eur J Heart Fail 2006;**8**(1):90-6.
3. Abe R, Enomoto K, Koyama H, et al. Effects of radiotherapy and of differences in the extent of surgery for early breast cancer on local recurrence and 15-year survival: An overview of the randomised trials. Lancet 2005;**366**(9503):2087-106.
4. Abrishamchian R, Kanhai D, Zwets E, et al. Low birth weight or diagnosis, which is a higher risk? - a meta-analysis of observational studies. Eur J Cardiothorac Surg 2006;**30**(5):700-05.
5. ACE Inhibitors in Diabetic Nephropathy Trialist G. Should all patients with type 1 diabetes mellitus and microalbuminuria receive angiotensin-converting enzyme inhibitors? A meta-analysis of individual patient data.[see comment]. Ann Intern Med 2001;**134**(5):370-9.
6. Achan J, Adam I, Arinaitwe E, et al. The Effect of Dosing Regimens on the Antimalarial Efficacy of Dihydroartemisinin-Piperaquine: A Pooled Analysis of Individual Patient Data. PLoS Med 2013;**10**(12):Article number e1001564.
7. Adachi JD, Rizzoli R, Boonen S, et al. Vertebral fracture risk reduction with risedronate in post-menopausal women with osteoporosis: A meta-analysis of individual patient data. Aging Clin Exp Res 2005;**17**(2):150-56.
8. Adachi JD, Roux C, Pitt PI, et al. A pooled data analysis on the use of intermittent cyclical etidronate therapy for the prevention and treatment of corticosteroid induced bone loss. J Rheumatol 2000;**27**(10):2424-31.
9. Adjuik M, Babiker A, Garner P, et al. Artesunate combinations for treatment of malaria: meta-analysis.[see comment]. Lancet 2004;**363**(9402):9-17.
10. Advanced Bladder Cancer (ABC) Meta-analysis Collaboration. Adjuvant chemotherapy for invasive bladder cancer (individual patient data). [Review] [25 refs]. Cochrane Database Syst Rev 2006(2):CD006018-.
11. Advanced Bladder Cancer Meta-analysis Collaboration. Neoadjuvant chemotherapy in invasive bladder cancer: a systematic review and meta-analysis. Lancet 2003;**361**(9373):1927-34.
12. Advanced Bladder Cancer Overview Collaboration. Neoadjuvant cisplatin for advanced bladder cancer. Cochrane Database Syst Rev 2000:Issue 2.
13. Advanced Colorectal Cancer Meta-Analysis Project. Modulation of fluorouracil by leucovorin in patients with advanced colorectal cancer: evidence in terms of response rate. . J Clin Oncol 1992;**10**(6):896-903.
14. Advanced Ovarian Cancer Trialists Group. Chemotherapy for advanced ovarian cancer. . Cochrane Database Syst Rev 2000(2):CD001418.
15. Ahlbom A, Day N, Feychting M, et al. A pooled analysis of magnetic fields and childhood leukaemia. Br J Cancer 2000;**83**(5):692.
16. Ahuja SD, Ashkin D, Avendano M, et al. Multidrug resistant pulmonary tuberculosis treatment regimens and patient outcomes: an individual patient data meta-analysis of 9,153 patients. PLoS Medicine / Public Library of Science 2012;**9**(8):e1001300.
17. Alberti W, Anderson G, Bartolucci A, et al. Chemotherapy in non-small cell lung cancer: a meta-analysis using updated data on individual patients from 52 randomised clinical trials. Br Med J 1995;**311**(7010):899.
18. Alessio L, Apostoli P, Braga M, et al. Estimation of pooled reference values for cadmium in blood using meta-analysis and TRACY criteria. Sci Total Environ 1994;**152**(2):169-77.
19. Alexopoulos D, Xanthopoulou I, Storey RF, et al. Platelet reactivity during ticagrelor maintenance therapy: a patient-level data meta-analysis. Am Heart J 2014;**168**(4):530-6.
20. Alfredsson J, Clayton T, Damman P, et al. Impact of an invasive strategy on 5 years outcome in men and women with non-ST-segment elevation acute coronary syndromes. Am Heart J 2014;**168**(4):522-9.
21. Alghamdi AA, McCrindle BW, Van Arsdell GS. Physiologic versus anatomic repair of congenitally corrected transposition of the great arteries: Meta-analysis of individual patient data. Ann Thorac Surg 2006;**81**(4):1529-35.
22. Ali AMG, Schmidt MK, Bolla MK, et al. Alcohol consumption and survival after a breast cancer diagnosis: A literature-based meta-analysis and collaborative analysis of data for 29,239 cases. Cancer Epidemiology Biomarkers and Prevention 2014;**23**(6):934-45.
23. Al-Jurf M, Aranha F, Annasetti C, et al. Allogeneic peripheral blood stem-cell compared with bone marrow transplantation in the management of hematologic malignancies: An individual patient data meta-analysis of nine randomized trials. J Clin Oncol 2005;**23**(22):5074-87.
24. Allgulander C, Hartford J, Russell J, et al. Pharmacotherapy of generalized anxiety disorder: Results of duloxetine treatment from a pooled analysis of three clinical trials. Curr Med Res Opin 2007;**23**(6):1245-52.

25. Amiodarone Trials Meta-Analysis Investigators. Effect of prophylactic amiodarone on mortality after acute myocardial infarction and in congestive heart failure: meta-analysis of individual data from 6500 patients in randomised trials. . Lancet 1997;**350**(9089):1417-24.
26. Amir E, Clemons M, Purdie CA, et al. Tissue confirmation of disease recurrence in breast cancer patients: pooled analysis of multi-centre, multi-disciplinary prospective studies. Cancer Treat Rev 2012;**38**(6):708-14.
27. AML Collaborative Group. A systematic collaborative overview of randomized trials comparing idarubicin with daunorubicin (or other anthracyclines) as induction therapy for acute myeloid leukaemia. . Br J Haematol 1998;**103**(1):100-9.
28. Anderson JJ, Wells G, Verhoeven AC, et al. Factors predicting response to treatment in rheumatoid arthritis: the importance of disease duration. Arthritis Rheum 2000;**43**(1):22-9.
29. Andes DR, Safdar N, Baddley JW, et al. Impact of treatment strategy on outcomes in patients with candidemia and other forms of invasive candidiasis: a patient-level quantitative review of randomized trials. Clin Infect Dis 2012;**54**(8):1110-22.
30. Andreoli MT, Itani KMF. Malignant eccrine spiradenoma: a meta-analysis of reported cases. Am J Surg 2011;**201**(5):695-9.
31. Andrews C, Aviles-Olmos I, Hariz M, et al. Which patients with dystonia benefit from deep brain stimulation? A metaregression of individual patient outcomes. Journal of Neurology, Neurosurgery and Psychiatry 2010;**81**(12):1383-9.
32. Annane D, Chevrolet J, Chevret S, et al. Nocturnal mechanical ventilation for chronic hypoventilation in patients with neuromuscular and chest wall disorders. Cochrane Database Syst Rev 2000.
33. Anonymous. Risk factors for stroke and efficacy of antithrombotic therapy in atrial fibrillation. Analysis of pooled data from five randomized controlled trials.[erratum appears in Arch Intern Med 1994 Oct 10;154(19):2254]. Arch Intern Med 1994;**154**(13):1449-57.
34. Antiplatelet Trialists' Collaboration. Collaborative overview of randomised trials of antiplatelet therapy Prevention of death, myocardial infarction, and stroke by prolonged antiplatelet therapy in various categories of patients. BMJ 1994;**308**(6921):81-106.
35. Antithrombotic Trialists Collaboration, Baigent C, Blackwell L, et al. Aspirin in the primary and secondary prevention of vascular disease: collaborative meta-analysis of individual participant data from randomised trials. Lancet 2009;**373**(9678):1849-60.
36. Antithrombotic Trialists' Collaboration. Collaborative meta-analysis of randomised trials of antiplatelet therapy for prevention of death, myocardial infarction, and stroke in high risk patients. BMJ 2002;**324**(7329):71-86.
37. Appleby P, Beral V, Berrington de Gonzalez A, et al. Carcinoma of the cervix and tobacco smoking: collaborative reanalysis of individual data on 13,541 women with carcinoma of the cervix and 23,017 women without carcinoma of the cervix from 23 epidemiological studies. Int J Cancer 2006;**118**(6):1481-95.
38. Ara RM, Reynolds AV, Conway P. The cost-effectiveness of etanercept in patients with severe ankylosing spondylitis in the UK. Rheumatology 2007;**46**(8):1338-44.
39. Ardizzoni A, Boni L, Tiseo M, et al. Cisplatin- versus carboplatin-based chemotherapy in first-line treatment of advanced non-small-cell lung cancer: an individual patient data meta-analysis.[see comment]. J Natl Cancer Inst 2007;**99**(11):847-57.
40. Arendts G, Dickson C, Howard K, et al. Transfer from residential aged care to emergency departments: an analysis of patient outcomes. Intern Med J 2012;**42**(1):75-82.
41. Arentz M, Pavlinac P, Kimerling ME, et al. Use of anti-retroviral therapy in tuberculosis patients on second-line anti-TB regimens: a systematic review. PLoS ONE [Electronic Resource] 2012;**7**(11):e47370.
42. Ariesen MJ, Algra A, Koudstaal PJ, et al. Risk of intracerebral hemorrhage in patients with arterial versus cardiac origin of cerebral ischemia on aspirin or placebo: analysis of individual patient data from 9 trials. [Review] [10 refs]. Stroke 2004;**35**(3):710-4.
43. Arima H, Murakami Y, Lam TH, et al. Effects of prehypertension and hypertension subtype on cardiovascular disease in the Asia-Pacific Region. Hypertension 2012;**59**(6):1118-23.
44. Armaganijan LV, Toff WD, Nielsen JC, et al. Are elderly patients at increased risk of complications following pacemaker implantation? A meta-analysis of randomized trials. Pacing Clin Electrophysiol 2012;**35**(2):131-4.
45. Armstrong MJ, Houlihan DD, Rowe IA, et al. Safety and efficacy of liraglutide in patients with type 2 diabetes and elevated liver enzymes: individual patient data meta-analysis of the LEAD program. Aliment Pharmacol Ther 2013;**37**(2):234-42.

46. Arnaud L, Mathian A, Devilliers H, et al. Patient-level analysis of five international cohorts further confirms the efficacy of aspirin for the primary prevention of thrombosis in patients with antiphospholipid antibodies. Autoimmun Rev 2015;**14**(3):192-200.
47. Arnott SJ, Duncan W, Gignoux M, et al. Preoperative radiotherapy for esophageal carcinoma.[update of Cochrane Database Syst Rev. 2000;(2):CD001799; PMID: 10796823]. [Review] [24 refs]. Cochrane Database Syst Rev 2000(4):CD001799.
48. Arnott SJ, Duncan W, Gignoux M, et al. Preoperative radiotherapy for esophageal carcinoma.[update of Cochrane Database Syst Rev. 2000;(4):CD001799; PMID: 11034728]. [Review] [35 refs]. Cochrane Database Syst Rev 2005(4):CD001799-.
49. Arrich J, Holzer M, Havel C, et al. Hypothermia for neuroprotection in adults after cardiopulmonary resuscitation. Cochrane Database Syst Rev 2012;**9**:CD004128.
50. Artemether-Quinine Meta-analysis Study G. A meta-analysis using individual patient data of trials comparing artemether with quinine in the treatment of severe falciparum malaria. Trans R Soc Trop Med Hyg 2001;**95**(6):637-50.
51. Asherson P, Bushe C, Saylor K, et al. Efficacy of atomoxetine in adults with attention deficit hyperactivity disorder: an integrated analysis of the complete database of multicenter placebo-controlled trials. J Psychopharmacol 2014;**28**(9):837-46.
52. Asherson P, Stes S, Markhed MN, et al. The effects of atomoxetine on emotional control in adults with ADHD: An integrated analysis of multicenter studies. Eur Psychiatry 2015;**30**(4):511-20.
53. Ashworth AB, Senan S, Palma DA, et al. An Individual Patient Data Metaanalysis of Outcomes and Prognostic Factors After Treatment of Oligometastatic Non-Small-Cell Lung Cancer. Clin Lung Cancer 2014.
54. Asia Pacific Cohort Studies Collaboration. An evaluation of metabolic risks for coronary death in the Asia Pacific region. Diabetes Res Clin Pract 2006;**74**(3):274-81.
55. Asia Pacific Cohort Studies Collaboration. Coronary risk prediction for those with and without diabetes. Eur J Cardiovasc Prev Rehabil 2006;**13**(1):30-6.
56. Asia Pacific Cohort Studies Collaboration. Impact of cigarette smoking on the relationship between body mass index and coronary heart disease: a pooled analysis of 3264 stroke and 2706 CHD events in 378579 individuals in the Asia Pacific region. BMC Public Health 2009;**9**:294.
57. Askie LM, Ballard RA, Cutter GR, et al. Inhaled nitric oxide in preterm infants: an individual-patient data meta-analysis of randomized trials. Pediatrics 2011;**128**(4):729-39.
58. Askie LM, Duley L, Henderson-Smart DJ, et al. Antiplatelet agents for prevention of pre-eclampsia: a meta-analysis of individual patient data.[see comment]. [Review] [59 refs]. Lancet 2007;**369**(9575):1791-98.
59. Askling J, Fahrbach K, Nordstrom B, et al. Cancer risk with tumor necrosis factor alpha (TNF) inhibitors: meta-analysis of randomized controlled trials of adalimumab, etanercept, and infliximab using patient level data. Pharmacoepidemiol Drug Saf 2011;**20**(2):119-30.
60. Astrup A, Gotzsche PC, van de Werken K, et al. Meta-analysis of resting metabolic rate in formerly obese subjects.[see comment]. Am J Clin Nutr 1999;**69**(6):1117-22.
61. Asvold BO, Vatten LJ, Bjoro T, et al. Thyroid function within the normal range and risk of coronary heart disease: an individual participant data analysis of 14 cohorts. JAMA Intern Med 2015;**175**(6):1037-47.
62. Athappan G, Habib M, Ponniah T, et al. Multi-detector computerized tomography angiography for evaluation of acute chest pain--a meta analysis and systematic review of literature. Int J Cardiol 2010;**141**(2):132-40.
63. Atherton PJ, Burger KN, Loprinzi CL, et al. Using the Skindex-16 and Common Terminology Criteria for Adverse Events to assess rash symptoms: results of a pooled-analysis (N0993). Support Care Cancer 2012;**20**(8):1729-35.
64. Atrial Fibrillation Investigators. The efficacy of aspirin in patients with atrial fibrillation. Analysis of pooled data from 3 randomized trials. . Arch Intern Med 1997;**157**(11):1237-40.
65. Auperin A, Le PC, Pignon JP, et al. Concomitant radio-chemotherapy based on platin compounds in patients with locally advanced non-small cell lung cancer (NSCLC): A meta-analysis of individual data from 1764 patients. Ann Oncol 2006;**17**(3):473-83.
66. Auperin A, Le Pechoux C, Rolland E, et al. Meta-analysis of concomitant versus sequential radiochemotherapy in locally advanced non-small-cell lung cancer. J Clin Oncol 2010;**28**(13):2181-90.
67. Bacci S, Di Paola R, Menzaghi C, et al. ENPP1 Q121 variant, increased pulse pressure and reduced insulin signaling, and nitric oxide synthase activity in endothelial cells. Arteriosclerosis, Thrombosis and Vascular Biology 2009;**29**(10):1678-83.
68. Badhiwala JH, Farrokhyar F, Alhazzani W, et al. Surgical outcomes and natural history of intramedullary spinal cord cavernous malformations: a single-center series and meta-analysis of individual patient data: Clinic article. Journal of Neurosurgery Spine 2014;**21**(4):662-76.

69. Bae EH, Theodore WH, Fregni F, et al. An estimate of placebo effect of repetitive transcranial magnetic stimulation in epilepsy. Epilepsy and Behavior 2011;**20**(2):355-9.
70. Bae JM, Choi YY, Kim H, et al. Mohs micrographic surgery for extramammary Paget disease: a pooled analysis of individual patient data. J Am Acad Dermatol 2013;**68**(4):632-7.
71. Baglin T, Douketis J, Tosetto A, et al. Does the clinical presentation and extent of venous thrombosis predict likelihood and type of recurrence? A patient-level meta-analysis. J Thromb Haemost 2010;**8**(11):2436-42.
72. Baigent C, Keech A, Kearney PM, et al. Efficacy and safety of cholesterol-lowering treatment: prospective meta-analysis of data from 90,056 participants in 14 randomised trials of statins. Lancet 2005;**366**(9493):1267-78.
73. Bailey M, McGuinness S, Haase M, et al. Sodium bicarbonate and renal function after cardiac surgery: a prospectively planned individual patient meta-analysis. Anesthesiology 2015;**122**(2):294-306.
74. Bakker JP, Edwards BA, Gautam SP, et al. Blood pressure improvement with continuous positive airway pressure is independent of obstructive sleep apnea severity. J Clin Sleep Med 2014;**10**(4):365-69.
75. Bakker NA, Veeger NJGM, Vergeer RA, et al. Prognosis after spinal cord and cauda compression in spontaneous spinal epidural hematomas. Neurology 2015;**84**(18):1894-903.
76. Bakker OJ, van Brunschot S, Farre A, et al. Timing of enteral nutrition in acute pancreatitis: meta-analysis of individuals using a single-arm of randomised trials. Pancreatology 2014;**14**(5):340-6.
77. Balachandra S, Siriwardena AK. Systematic appraisal of the management of the major vascular complications of pancreatitis. Am J Surg 2005;**190**(3):489-95.
78. Baman TS, Meier P, Romero J, et al. Safety of pacemaker reuse: a meta-analysis with implications for underserved nations. Circulation: Arrhythmia and Electrophysiology 2011;**4**(3):318-23.
79. Bansback NJ, Ara R, Barkham N, et al. Estimating the cost and health status consequences of treatment with TNF antagonists in patients with psoriatic arthritis. Rheumatology 2006;**45**(8):1029-38.
80. Barnett GC, Elliott RM, Alsner J, et al. Individual patient data meta-analysis shows no association between the SNP rs1800469 in TGFB and late radiotherapy toxicity. Radiother Oncol 2012;**105**(3):289-95.
81. Barnett PG, Rodgers JH, Bloch DA. A meta-analysis comparing buprenorphine to methadone for treatment of opiate dependence. Addiction 2001;**96**(5):683-90.
82. Barshes NR, Horwitz IB, Franzini L, et al. Waitlist mortality decreases with increased use of extended criteria donor liver grafts at adult liver transplant centers. Am J Transplant 2007;**7**(5):1265-70.
83. Barta SK, Xue X, Wang D, et al. Treatment factors affecting outcomes in HIV-associated non-Hodgkin lymphomas: a pooled analysis of 1546 patients. Blood 2013;**122**(19):3251-62.
84. Bartlett JMS, McConkey CC, Munro AF, et al. Predicting Anthracycline Benefit: TOP2A and CEP17-Not Only but Also. J Clin Oncol 2015;**33**(15):1680-7.
85. Barton I. The relationship between osteoporotic fracture risk and a surrogate: Apparent discrepancies between analyses based on individual patient data and summary statisrics. Pharm Stat 2004;**3**(3):205-12.
86. Barzi F, Patel A, Woodward M, et al. A comparison of lipid variables as predictors of cardiovascular disease in the Asia Pacific region. Ann Epidemiol 2005;**15**(5):405-13.
87. Bastos ML, Hussain H, Weyer K, et al. Treatment outcomes of patients with multidrug-resistant and extensively drug-resistant tuberculosis according to drug susceptibility testing to first- and second-line drugs: an individual patient data meta-analysis. Clin Infect Dis 2014;**59**(10):1364-74.
88. Bauer TM, El-Rayes BF, Li X, et al. Carbohydrate antigen 19-9 is a prognostic and predictive biomarker in patients with advanced pancreatic cancer who receive gemcitabine-containing chemotherapy: a pooled analysis of 6 prospective trials. Cancer 2013;**119**(2):285-92.
89. Baujat B, Audry H, Bourhis J, et al. Chemotherapy in locally advanced nasopharyngeal carcinoma: An individual patient data meta-analysis of eight randomized trials and 1753 patients. International Journal of Radiation Oncology Biology Physics 2006;**64**(1):47-56.
90. Baujat B, Bourhis J, Blanchard P, et al. Hyperfractionated or accelerated radiotherapy for head and neck cancer. Cochrane Database Syst Rev 2010(12):CD002026.
91. Bazelier MT, de Vries F, Vestergaard P, et al. Risk of fracture with thiazolidinediones: An individual patient data meta-analysis. Front Endocrinol (Lausanne) 2013;**4**(FEB).
92. Becquet R, Marston M, Dabis F, et al. Children who acquire HIV infection perinatally are at higher risk of early death than those acquiring infection through breastmilk: a meta-analysis. PLoS One 2012;**7**(2):e28510.
93. Bedaiwy MA, El-Nashar SA, El Saman AM, et al. Reproductive outcome after transplantation of ovarian tissue: A systematic review. Hum Reprod 2008;**23**(12):2709-17.
94. Beer TM, Berry W, Wersinger EM, et al. Weekly docetaxel in elderly patients with prostate cancer: efficacy and toxicity in patients at least 70 years of age compared with patients younger than 70 years. Clin Prostate Cancer 2003;**2**(3):167-72.

95. Bell KJL, Hayen A, Macaskill P, et al. Monitoring initial response to Angiotensin-converting enzyme inhibitor-based regimens: an individual patient data meta-analysis from randomized, placebo-controlled trials. Hypertension 2010;**56**(3):533-9.
96. Bendtsen F, D'Amico G, Rusch E, et al. Effect of recombinant Factor VIIa on outcome of acute variceal bleeding: An individual patient based meta-analysis of two controlled trials. J Hepatol 2014.
97. Benhamou S, Lee WJ, Alexandrie AK, et al. Meta- and pooled analyses of the effects of glutathione S-transferase M1 polymorphisms and smoking on lung cancer risk. Carcinogenesis 2002;**23**(8):1343-50.
98. Benner A, Mansouri L, Rossi D, et al. MDM2 promotor polymorphism and disease characteristics in chronic lymphocytic leukemia: results of an individual patient data-based meta-analysis. Haematologica 2014;**99**(8):1285-91.
99. Bennett K, Manassis K, Walter SD, et al. Cognitive behavioral therapy age effects in child and adolescent anxiety: an individual patient data metaanalysis. Depress Anxiety 2013;**30**(9):829-41.
100. Ben-Shlomo Y, Spears M, Boustred C, et al. Aortic pulse wave velocity improves cardiovascular event prediction: an individual participant meta-analysis of prospective observational data from 17,635 subjects. J Am Coll Cardiol 2014;**63**(7):636-46.
101. Bensinger WI, Al-Jurf M, Annasetti C, et al. Individual patient data meta-analysis of allogeneic peripheral blood stem cell transplant vs bone marrow transplant in the management of hematological malignancies: Indirect assessment of the effect of day 11 methotrexate administration. Bone Marrow Transplant 2006;**38**(8):539-46.
102. Beral V, Bull D, Doll R, et al. Breast cancer and hormone replacement therapy: collaborative reanalysis of data from 51 epidemiological studies of 52, 705 women with breast cancer and 108, 411 women without breast cancer. Lancet 1997;**350**(9084):1047-59.
103. Beral V, Doll R, Hermon C, et al. Ovarian cancer and oral contraceptives: collaborative reanalysis of data from 45 epidemiological studies including 23,257 women with ovarian cancer and 87,303 controls. Lancet 2008;**371**(9609):303-14.
104. Berghella V, Odibo AO, To MS, et al. Cerclage for short cervix on ultrasonography: Meta-analysis of trials using individual patient-level data. Obstet Gynecol 2005;**106**(1):181-89.
105. Berry DA, Ueno NT, Johnson MM, et al. High-dose chemotherapy with autologous stem-cell support as adjuvant therapy in breast cancer: overview of 15 randomized trials. J Clin Oncol 2011;**29**(24):3214-23.
106. Berry SM, Broglio K, Bunker M, et al. A patient-level meta-analysis of studies evaluating vagus nerve stimulation therapy for treatment-resistant depression. Medical Devices: Evidence and Research 2013;**6**(1):17-35.
107. Best L, Simmonds P, Baughan C, et al. Palliative chemotherapy for advanced or metastatic colorectal cancer. Cochrane Database Syst Rev 2000;**2**.
108. Beveridge LA, Struthers AD, Khan F, et al. Effect of Vitamin D Supplementation on Blood Pressure: A Systematic Review and Meta-analysis Incorporating Individual Patient Data. JAMA Intern Med 2015;**175**(5):745-54.
109. Beyerlein A, Hadders-Algra M, Kennedy K, et al. Infant formula supplementation with long-chain polyunsaturated fatty acids has no effect on Bayley developmental scores at 18 months of age--IPD meta-analysis of 4 large clinical trials. J Pediatr Gastroenterol Nutr 2010;**50**(1):79-84.
110. Bhutta ZA, Bird SM, Black RE, et al. Therapeutic effects of oral zinc in acute and persistent diarrhea in children in developing countries: pooled analysis of randomized controlled trials. The American journal of clinical nutrition 2000;**72**(6):1516-22.
111. Biau DJ, Katsahian S, Kartus J, et al. Patellar tendon versus hamstring tendon autografts for reconstructing the anterior cruciate ligament: a meta-analysis based on individual patient data. Am J Sports Med 2009;**37**(12):2470-8.
112. Bidard F-C, Peeters DJ, Fehm T, et al. Clinical validity of circulating tumour cells in patients with metastatic breast cancer: a pooled analysis of individual patient data. Lancet Oncol 2014;**15**(4):406-14.
113. Bingham CO, 3rd, Smugar SS, Wang H, et al. Early response to COX-2 inhibitors as a predictor of overall response in osteoarthritis: pooled results from two identical trials comparing etoricoxib, celecoxib and placebo. Rheumatology (Oxford) 2009;**48**(9):1122-7.
114. Birks J, Flicker L. Selegiline for Alzheimer's disease. Cochrane Database Syst Rev 2003(1):Issue 1.
115. Biros E, Norman PE, Jones GT, et al. Meta-analysis of the association between single nucleotide polymorphisms in TGF-beta receptor genes and abdominal aortic aneurysm. Atherosclerosis 2011;**219**(1):218-23.
116. Bischoff-Ferrari HA, Willett WC, Orav EJ, et al. A pooled analysis of vitamin D dose requirements for fracture prevention. N Engl J Med 2012;**367**(1):40-9.

117. Bisset L, Smidt N, Van der Windt DA, et al. Conservative treatments for tennis elbow - Do subgroups of patients respond differently? Rheumatology 2007;**46**(10):1601-05.
118. Blacher J, Staessen JA, Girerd X, et al. Pulse pressure not mean pressure determines cardiovascular risk in older hypertensive patients. Arch Intern Med 2000;**160**(8):1085-9.
119. Black JA, Herbison GP, Lyons RA, et al. Recovery after injury: an individual patient data meta-analysis of general health status using the EQ-5D. Journal of Trauma-Injury Infection and Critical Care 2011;**71**(4):1003-10.
120. Blanchard P, Bourhis J, Lacas B, et al. Taxane-cisplatin-fluorouracil as induction chemotherapy in locally advanced head and neck cancers: an individual patient data meta-analysis of the meta-analysis of chemotherapy in head and neck cancer group. J Clin Oncol 2013;**31**(23):2854-60.
121. Blanchard P, Lee A, Marguet S, et al. Chemotherapy and radiotherapy in nasopharyngeal carcinoma: an update of the MAC-NPC meta-analysis. Lancet Oncol 2015;**16**(6):645-55.
122. Blanke CD, Bot BM, Thomas DM, et al. Impact of young age on treatment efficacy and safety in advanced colorectal cancer: a pooled analysis of patients from nine first-line phase III chemotherapy trials. J Clin Oncol 2011;**29**(20):2781-6.
123. Bliss JM, Ford D, Swerdlow AJ, et al. Risk of cutaneous melanoma associated with pigmentation characteristics and freckling: systematic overview of 10 case-control studies. The International Melanoma Analysis Group (IMAGE). Int J Cancer 1995;**62**(4):367-76.
124. Blood Pressure Lowering Treatment Trialists Collaboration, Ninomiya T, Perkovic V, et al. Blood pressure lowering and major cardiovascular events in people with and without chronic kidney disease: meta-analysis of randomised controlled trials. BMJ 2013;**347**:f5680.
125. Blood Pressure Lowering Treatment Trialists Collaboration, Sundstrom J, Arima H, et al. Blood pressure-lowering treatment based on cardiovascular risk: a meta-analysis of individual patient data. Lancet 2014;**384**(9943):591-8.
126. Blood Pressure Lowering Treatment Trialists Collaboration, Ying A, Arima H, et al. Effects of blood pressure lowering on cardiovascular risk according to baseline body-mass index: a meta-analysis of randomised trials. Lancet 2015;**385**(9971):867-74.
127. Blows FM, Driver KE, Schmidt MK, et al. Subtyping of breast cancer by immunohistochemistry to investigate a relationship between subtype and short and long term survival: a collaborative analysis of data for 10,159 cases from 12 studies. PLoS Med 2010;**7**(5):e1000279.
128. Blum JL, Barrios CH, Feldman N, et al. Pooled analysis of individual patient data from capecitabine monotherapy clinical trials in locally advanced or metastatic breast cancer. Breast Cancer Res Treat 2012;**136**(3):777-88.
129. Blum MR, Bauer DC, Collet T-H, et al. Subclinical thyroid dysfunction and fracture risk: a meta-analysis. JAMA 2015;**313**(20):2055-65.
130. Boccia S, Hung R, Ricciardi G, et al. Meta- and pooled analyses of the methylenetetrahydrofolate reductase C677T and A1298C polymorphisms and gastric cancer risk: a huge-GSEC review. Am J Epidemiol 2008;**167**(5):505-16.
131. Boeker EB, Ram K, Klopotowska JE, et al. An individual patient data meta-analysis on factors associated with adverse drug events in surgical and non-surgical inpatients. Br J Clin Pharmacol 2015;**79**(4):548-57.
132. Boekholdt SM, Arsenault BJ, Mora S, et al. Association of LDL cholesterol, non-HDL cholesterol, and apolipoprotein B levels with risk of cardiovascular events among patients treated with statins: a meta-analysis.[Erratum appears in JAMA. 2012 Apr 25;307(16):1694], [Erratum appears in JAMA. 2012 May 9;307(18):1915]. JAMA 2012;**307**(12):1302-9.
133. Boekholdt SM, Hovingh GK, Mora S, et al. Very low levels of atherogenic lipoproteins and the risk for cardiovascular events: a meta-analysis of statin trials. J Am Coll Cardiol 2014;**64**(5):485-94.
134. Boekholdt SM, Sacks FM, Jukema JW, et al. Cholesteryl ester transfer protein TaqIB variant, high-density lipoprotein cholesterol levels, cardiovascular risk, and efficacy of pravastatin treatment: Individual patient meta-analysis of 13 677 subjects. Circulation 2005;**111**(3):278-87.
135. Boersma E. Does time matter? A pooled analysis of randomized clinical trials comparing primary percutaneous coronary intervention and in-hospital fibrinolysis in acute myocardial infarction patients. Eur Heart J 2006;**27**(7):779-88.
136. Boersma E, Harrington RA, Moliterno DJ, et al. Platelet glycoprotein IIb/IIIa inhibitors in acute coronary syndromes: a meta-analysis of all major randomised clinical trials.[see comment][erratum appears in Lancet 2002 Jun 15;359(9323):2120]. Lancet 2002;**359**(9302):189-98.
137. Bogaerts J, Ford R, Sargent D, et al. Individual patient data analysis to assess modifications to the RECIST criteria. Eur J Cancer 2009;**45**(2):248-60.

138. Boghani Z, Husain Q, Kanumuri VV, et al. Juvenile nasopharyngeal angiofibroma: a systematic review and comparison of endoscopic, endoscopic-assisted, and open resection in 1047 cases. Laryngoscope 2013;**123**(4):859-69.
139. Bohlius J, Schmidlin K, Brillant C, et al. Erythropoietin or Darbepoetin for patients with cancer--meta-analysis based on individual patient data. Cochrane Database Syst Rev 2009(3):CD007303.
140. Bokemeyer C, Kollmannsberger C, Stenning S, et al. Metastatic seminoma treated with either single agent carboplatin or cisplatin-based combination chemotherapy: a pooled analysis of two randomised trials. Br J Cancer 2004;**91**(4):683-7.
141. Bokemeyer C, Van Cutsem E, Rougier P, et al. Addition of cetuximab to chemotherapy as first-line treatment for KRAS wild-type metastatic colorectal cancer: pooled analysis of the CRYSTAL and OPUS randomised clinical trials. Eur J Cancer 2012;**48**(10):1466-75.
142. Bolland MJ, Avenell A, Baron JA, et al. Effect of calcium supplements on risk of myocardial infarction and cardiovascular events: meta-analysis. BMJ 2010;**341**:c3691.
143. Bolton KL, Chenevix-Trench G, Goh C, et al. Association between BRCA1 and BRCA2 mutations and survival in women with invasive epithelial ovarian cancer. JAMA 2012;**307**(4):382-90.
144. Bongartz T, Warren FC, Mines D, et al. Etanercept therapy in rheumatoid arthritis and the risk of malignancies: a systematic review and individual patient data meta-analysis of randomised controlled trials. Ann Rheum Dis 2009;**68**(7):1177-83.
145. Bonnetain F, Bosset JF, Gerard JP, et al. What is the clinical benefit of preoperative chemoradiotherapy with 5FU/leucovorin for T3-4 rectal cancer in a pooled analysis of EORTC 22921 and FFCD 9203 trials: surrogacy in question? Eur J Cancer 2012;**48**(12):1781-90.
146. Booij L, Van der Does W, Benkelfat C, et al. Predictors of mood response to acute tryptophan depletion. A reanalysis. Neuropsychopharmacology 2002;**27**(5):852-61.
147. Boonacker CW, Rovers MM, Browning GG, et al. Adenoidectomy with or without grommets for children with otitis media: an individual patient data meta-analysis. Health Technol Assess 2014;**18**(5):1-118.
148. Boonstra N, Klaassen R, Sytema S, et al. Duration of untreated psychosis and negative symptoms--a systematic review and meta-analysis of individual patient data. Schizophr Res 2012;**142**(1-3):12-9.
149. Bosetti C, Negri E, Kolonel L, et al. A pooled analysis of case-control studies of thyroid cancer. VII. Cruciferous and other vegetables (International). Cancer Causes Control 2002;**13**(8):765-75.
150. Bossie CA, Turkoz I, Lindenmayer J-P, et al. Palperidone ER and oral risperidone in patients with schizophrenia: A comparitive database analysis. BMC Psychiatry 2011;**11**.
151. Bourhis J, Blanchard P, Maillard E, et al. Effect of amifostine on survival among patients treated with radiotherapy: a meta-analysis of individual patient data. J Clin Oncol 2011;**29**(18):2590-7.
152. Bourhis J, Overgaard J, Audry H, et al. Hyperfractionated or accelerated radiotherapy in head and neck cancer: a meta-analysis. Lancet 2006;**368**(9538):843-54.
153. Bourne C, Aydemir O, Balanza-Martinez V, et al. Neuropsychological testing of cognitive impairment in euthymic bipolar disorder: an individual patient data meta-analysis. Acta Psychiatr Scand 2013;**128**(3):149-62.
154. Boutitie F, Gueyffier F, Pocock S, et al. J-shaped relationship between blood pressure and mortality in hypertensive patients: new insights from a meta-analysis of individual-patient data.[see comment][summary for patients in Ann Intern Med. 2002 Mar 19;136(6):I49; PMID: 11900515]. Ann Intern Med 2002;**136**(6):438-48.
155. Boutitie F, Pinede L, Schulman S, et al. Influence of preceding length of anticoagulant treatment and initial presentation of venous thromboembolism on risk of recurrence after stopping treatment: analysis of individual participants' data from seven trials. BMJ 2011;**342**:d3036.
156. Bova C, Sanchez O, Prandoni P, et al. Identification of intermediate-risk patients with acute symptomatic pulmonary embolism. Eur Respir J 2014;**44**(3):694-703.
157. Bower P, Byford S, Barber J, et al. Meta-analysis of data on costs from trials of counselling in primary care: using individual patient data to overcome sample size limitations in economic analyses.[see comment]. BMJ 2003;**326**(7401):1247-50.
158. Bower P, Kontopantelis E, Sutton A, et al. Influence of initial severity of depression on effectiveness of low intensity interventions: meta-analysis of individual patient data. BMJ 2013;**346**:f540.
159. Brand JS, Rovers MM, Yeap BB, et al. Testosterone, sex hormone-binding globulin and the metabolic syndrome in men: An individual participant data meta-analysis of observational studies. PLoS One 2014;**9**(7).
160. Brar SS, ten Berg J, Marcucci R, et al. Impact of platelet reactivity on clinical outcomes after percutaneous coronary intervention. A collaborative meta-analysis of individual participant data. J Am Coll Cardiol 2011;**58**(19):1945-54.

161. Bratton DJ, Stradling JR, Barbe F, et al. Effect of CPAP on blood pressure in patients with minimally symptomatic obstructive sleep apnoea: a meta-analysis using individual patient data from four randomised controlled trials. Thorax 2014;**69**(12):1128-35.
162. Braun S, Vogl FD, Naume B, et al. A pooled analysis of bone marrow micrometastasis in breast cancer. N Engl J Med 2005;**353**(8):793-802.
163. Breugom AJ, Swets M, Bosset J-F, et al. Adjuvant chemotherapy after preoperative (chemo)radiotherapy and surgery for patients with rectal cancer: a systematic review and meta-analysis of individual patient data. Lancet Oncol 2015;**16**(2):200-7.
164. Briel M, Meade M, Mercat A, et al. Higher vs lower positive end-expiratory pressure in patients with acute lung injury and acute respiratory distress syndrome: systematic review and meta-analysis. JAMA 2010;**303**(9):865-73.
165. Brin MF, Boodhoo TI, Pogoda JM, et al. Safety and tolerability of onabotulinumtoxinA in the treatment of facial lines: a meta-analysis of individual patient data from global clinical registration studies in 1678 participants. J Am Acad Dermatol 2009;**61**(6):961-70.e1-11.
166. Bringhen S, Mateos MV, Zweegman S, et al. Age and organ damage correlate with poor survival in myeloma patients: meta-analysis of 1435 individual patient data from 4 randomized trials. Haematologica 2013;**98**(6):980-7.
167. Brinton LA, Cook MB, McCormack V, et al. Anthropometric and hormonal risk factors for male breast cancer: Male breast cancer pooling project results. J Natl Cancer Inst 2014;**106**(3).
168. Broer SL, Dolleman M, van Disseldorp J, et al. Prediction of an excessive response in in vitro fertilization from patient characteristics and ovarian reserve tests and comparison in subgroups: an individual patient data meta-analysis. Fertil Steril 2013;**100**(2):420-9.e7.
169. Broer SL, van Disseldorp J, Broeze KA, et al. Added value of ovarian reserve testing on patient characteristics in the prediction of ovarian response and ongoing pregnancy: an individual patient data approach. Hum Reprod Update 2013;**19**(1):26-36.
170. Broeze KA, Opmeer BC, Coppus SFPJ, et al. Chlamydia antibody testing and diagnosing tubal pathology in subfertile women: an individual patient data meta-analysis. Hum Reprod Update 2011;**17**(3):301-10.
171. Broeze KA, Opmeer BC, Van Geloven N, et al. Are patient characteristics associated with the accuracy of hysterosalpingography in diagnosing tubal pathology? An individual patient data meta-analysis. Hum Reprod Update 2011;**17**(3):293-300.
172. Broutet N, Tchamgoue S, Pereira E, et al. Risk factors for failure of Helicobacter pylori therapy--results of an individual data analysis of 2751 patients. Aliment Pharmacol Ther 2003;**17**(1):99-109.
173. Brouwer IA, Raitt MH, Dullemeijer C, et al. Effect of fish oil on ventricular tachyarrhythmia in three studies in patients with implantable cardioverter defibrillators. Eur Heart J 2009;**30**(7):820-6.
174. Brunotto M, Zarate AM, Bono A, et al. Risk genes in head and neck cancer: A systematic review and meta-analysis of last 5 years. Oral Oncol 2014;**50**(3):178-88.
175. Bruzzi P, Del Mastro L, Sormani MP, et al. Objective response to chemotherapy as a potential surrogate end point of survival in metastatic breast cancer patients. J Clin Oncol 2005;**23**(22):5117-25.
176. Bueters RRG, Van De Kar NCAJ, Schreuder MF. Adult renal size is not a suitable marker for nephron numbers: An individual patient data meta-analysis. Kidney Blood Press Res 2013;**37**(6):540-46.
177. Burdett S, Pignon JP, Tierney J, et al. Adjuvant chemotherapy for resected early-stage non-small cell lung cancer. Cochrane Database Syst Rev 2015;**3**.
178. Burdett S, Stephens R, Stewart L, et al. Chemotherapy in addition to supportive care improves survival in advanced non-small-cell lung cancer: A systematic review and meta-analysis of individual patient data from 16 randomized controlled trials. J Clin Oncol 2008;**26**(28):4617-25.
179. Burdett S, Stewart L, on behalf of the PM-aG. Postoperative radiotherapy in non-small-cell lung cancer: update of an individual patient data meta-analysis. Lung Cancer 2005;**47**(1):81-3.
180. Burns A, Yeates A, Akintade L, et al. Defining treatment response to donepezil in Alzheimer's disease: responder analysis of patient-level data from randomized, placebo-controlled studies. Drugs Aging 2008;**25**(8):707-14.
181. Burzotta F, De Vita M, Gu YL, et al. Clinical impact of thrombectomy in acute ST-elevation myocardial infarction: an individual patient-data pooled analysis of 11 trials. Eur Heart J 2009;**30**(18):2193-203.
182. Burzykowski T, Buyse M, Piccart-Gebhart MJ, et al. Evaluation of tumor response, disease control, progression-free survival, and time to progression as potential surrogate end points in metastatic breast cancer. J Clin Oncol 2008;**26**(12):1987-92.
183. Bustamante A, Sobrino T, Giralt D, et al. Prognostic value of blood interleukin-6 in the prediction of functional outcome after stroke: a systematic review and meta-analysis. J Neuroimmunol 2014;**274**(1-2):215-24.

184. Butali A, Little J, Chevrier C, et al. Folic acid supplementation use and the MTHFR C677T polymorphism in orofacial clefts etiology: An individual participant data pooled-analysis. Birth Defects Research 2013;**97**(8):509-14.
185. Butcher I, Maas AIR, Lu J, et al. Prognostic value of admission blood pressure in traumatic brain injury: Results from the IMPACT study. J Neurotrauma 2007;**24**(2):294-302.
186. Butcher I, McHugh GS, Lu J, et al. Prognostic value of cause of injury in traumatic brain injury: results from the IMPACT study. J Neurotrauma 2007;**24**(2):281-6.
187. Butturini G, Stocken DD, Wente MN, et al. Influence of resection margins and treatment on survival in patients with pancreatic cancer: meta-analysis of randomized controlled trials. Arch Surg 2008;**143**(1):75-83; discussion 83.
188. Buyse M. Cyclophosphamide Plus Cisplatin Versus Cyclophosphomide, Doxorubicin, and Cisplatin Chemotherapy of Ovarian-carcinoma-a Metaanalysis. J Clin Oncol 1991;**9**(9):1668-74.
189. Buyse M, Burzykowski T, Carroll K, et al. Progression-free survival is a surrogate for survival in advanced colorectal cancer. J Clin Oncol 2007;**25**(33):5218-24.
190. Buyse M, Squifflet P, Lange BJ, et al. Individual patient data meta-analysis of randomized trials evaluating IL-2 monotherapy as remission maintenance therapy in acute myeloid leukemia. Blood 2011;**117**(26):7007-13.
191. Buyse M, Thirion P, Carlson RW, et al. Relation between tumour response to first-line chemotherapy and survival in advanced colorectal cancer: a meta-analysis. Lancet 2000;**356**(9227):373-78.
192. C Reactive Protein Coronary Heart Disease Genetics Collaboration, Wensley F, Gao P, et al. Association between C reactive protein and coronary heart disease: mendelian randomisation analysis based on individual participant data. BMJ 2011;**342**:d548.
193. C. L. L. Trialists' Collaborative Group. Systematic review of purine analog treatment for chronic lymphocytic leukemia: lessons for future trials. Haematologica 2012;**97**(3):428-36.
194. Calatayud CM, Garcia AM, Aragones AM, et al. Safety and efficacy profile and immunological changes associated with oral immunotherapy for IgE-mediated cow's milk allergy in children: systematic review and meta-analysis. J Investig Allergol Clin Immunol 2014;**24**(5):298-307.
195. Caldon LJ, Walters SJ, Reed JA, et al. Case-mix fails to explain variation in mastectomy rates: management of screen-detected breast cancer in a UK region 1997-2003. Br J Cancer 2005;**92**(1):55-9.
196. Caleyachetty R, Echouffo-Tcheugui JB, Tait CA, et al. Prevalence of behavioural risk factors for cardiovascular disease in adolescents in low-income and middle-income countries: An individual participant data meta-analysis. Lancet Diabetes Endocrinol 2015;**3**(7):535-44.
197. Callan MJ, Shead NW, Olson JM. The relation between personal relative deprivation and the urge to gamble among gamblers is moderated by problem gambling severity: A meta-analysis. Addict Behav 2015;**45**:146-49.
198. Camacho M, Teixeira J, Abdullatif J, et al. Maxillomandibular advancement and tracheostomy for morbidly obese obstructive sleep apnea: a systematic review and meta-analysis. Otolaryngology - Head and Neck Surgery 2015;**152**(4):619-30.
199. Camma C, Bruno S, Schepis F, et al. Retreatment with interferon plus ribavirin of chronic hepatitis C non-responders to interferon monotherapy: a meta-analysis of individual patient data. Gut 2002;**51**(6):864-9.
200. Camma C, Di Bona D, Schepis F, et al. Effect of peginterferon alfa-2a on liver histology in chronic hepatitis C: a meta-analysis of individual patient data. Hepatology 2004;**39**(2):333-42.
201. Camma C, Giunta M, Chemello L, et al. Chronic hepatitis C: interferon retreatment of relapsers. A meta-analysis of individual patient data. European Concerted Action on Viral Hepatitis (EUROHEP). Hepatology 1999;**30**(3):801-7.
202. Canaud L, Karthikesalingam A, Jackson D, et al. Clinical outcomes of single versus staged hybrid repair for thoracoabdominal aortic aneurysm. J Vasc Surg 2013;**58**(5):1192-200.
203. Cancer CGoHFiB. Breast cancer and hormonal contraceptives: collaborative reanalysis of individual data on 53 297 women with breast cancer and 100 239 women without breast cancer from 54 epidemiological studies. Lancet 1996;**347**(9017):1713-27.
204. Cancer. CGoESoO. Ovarian cancer and body size: individual participant meta-analysis including 25,157 women with ovarian cancer from 47 epidemiological studies. PLoS Med 2012;**9**(4):e1001200.
205. Cardarelli MG, Young AJ, Griffith B. Use of extracorporeal membrane oxygenation for adults in cardiac arrest (E-CPR): a meta-analysis of observational studies. ASAIO J 2009;**55**(6):581-6.
206. Cardwell CR, Stene LC, Joner G, et al. Maternal age at birth and childhood type 1 diabetes: a pooled analysis of 30 observational studies. Diabetes 2010;**59**(2):486-94.
207. Cardwell CR, Stene LC, Joner G, et al. Birth order and childhood type 1 diabetes risk: a pooled analysis of 31 observational studies. Int J Epidemiol 2011;**40**(2):363-74.

208. Cardwell CR, Stene LC, Joner G, et al. Birthweight and the risk of childhood-onset type 1 diabetes: a meta-analysis of observational studies using individual patient data. Diabetologia 2010;**53**(4):641-51.
209. Cardwell CR, Stene LC, Ludvigsson J, et al. Breast-feeding and childhood-onset type 1 diabetes: a pooled analysis of individual participant data from 43 observational studies. Diabetes Care 2012;**35**(11):2215-25.
210. Carnero-Pardo C, Lopez-Alcalde S, Allegri RF, et al. A systematic review and meta-analysis of the diagnostic accuracy of the phototest for cognitive impairment and dementia. Dementia e Neuropsychologia 2014;**8**(2):141-47.
211. Caro JJ, Huybrechts KF, Green TC. Estimates of the effect on hepatic iron of oral deferiprone compared with subcutaneous desferrioxamine for treatment of iron overload in thalassemia major: A systematic review. BMC Blood Disord 2002;**2**(4).
212. Carotid Stenting Trialists Collaboration, Bonati LH, Dobson J, et al. Short-term outcome after stenting versus endarterectomy for symptomatic carotid stenosis: a preplanned meta-analysis of individual patient data. Lancet 2010;**376**(9746):1062-73.
213. Cassese S, Desch S, Kastrati A, et al. Polymer-free sirolimus-eluting versus polymer-based paclitaxel-eluting stents: An individual patient data analysis of randomized trials. Revista Espanola de Cardiologia 2013;**66**(6):435-42.
214. Cassidy J, Saltz L, Twelves C, et al. Efficacy of capecitabine versus 5-fluorouracil in colorectal and gastric cancers: a meta-analysis of individual data from 6171 patients. Ann Oncol 2011;**22**(12):2604-9.
215. Castellsague X, Diaz M, Vaccarella S, et al. Intrauterine device use, cervical infection with human papillomavirus, and risk of cervical cancer: a pooled analysis of 26 epidemiological studies. Lancet Oncol 2011;**12**(11):1023-31.
216. Cates CJ, Cates MJ. Regular treatment with salmeterol for chronic asthma: Serious adverse events. Cochrane Database Syst Rev 2008(3):-.
217. Cazzola M, Floriani I, Page CP. The therapeutic efficacy of erdosteine in the treatment of chronic obstructive bronchitis: a meta-analysis of individual patient data. Pulm Pharmacol Ther 2010;**23**(2):135-44.
218. Celio L, Bonizzoni E, Bajetta E, et al. Palonosetron plus single-dose dexamethasone for the prevention of nausea and vomiting in women receiving anthracycline/cyclophosphamide-containing chemotherapy: meta-analysis of individual patient data examining the effect of age on outcome in two phase III trials. Support Care Cancer 2013;**21**(2):565-73.
219. Chaker L, Baumgartner C, Den Elzen WPJ, et al. Subclinical hypothyroidism and the risk of stroke events and fatal stroke: An individual participant data analysis. J Clin Endocrinol Metab 2015;**100**(6):2181-91.
220. Chalidis B, Stengel D, Giannoudis PV. Early excision and late excision of heterotopic ossification after traumatic brain injury are equivalent: A systematic review of the literature. J Neurotrauma 2007;**24**(11):1675-86.
221. Chambrone L, Pannuti CM, Tu Y-K, et al. Evidence-based periodontal plastic surgery. II. An individual data meta-analysis for evaluating factors in achieving complete root coverage. J Periodontol 2012;**83**(4):477-90.
222. Chambrone L, Preshaw PM, Rosa EF, et al. Effects of smoking cessation on the outcomes of non-surgical periodontal therapy: a systematic review and individual patient data meta-analysis. J Clin Periodontol 2013;**40**(6):607-15.
223. Chang K-C, Yew W-W, Tam C-M, et al. WHO group 5 drugs and difficult multidrug-resistant tuberculosis: a systematic review with cohort analysis and meta-analysis. Antimicrob Agents Chemother 2013;**57**(9):4097-104.
224. Chappell FM, Wardlaw JM, Young GR, et al. Carotid artery stenosis: accuracy of noninvasive tests--individual patient data meta-analysis. Radiology 2009;**251**(2):493-502.
225. Chau I, Norman AR, Cunningham D, et al. The impact of primary tumour origins in patients with advanced oesophageal, oesophago-gastric junction and gastric adenocarcinoma--individual patient data from 1775 patients in four randomised controlled trials. Ann Oncol 2009;**20**(5):885-91.
226. Chau I, Norman AR, Cunningham D, et al. Multivariate prognostic factor analysis in locally advanced and metastatic esophago-gastric cancer--pooled analysis from three multicenter, randomized, controlled trials using individual patient data. J Clin Oncol 2004;**22**(12):2395-403.
227. Chemoradiotherapy for Cervical Cancer Meta-analysis Collaboration. Reducing uncertainties about the effects of chemoradiotherapy for cervical cancer: individual patient data meta-analysis. Cochrane Database Syst Rev 2010(1):CD008285.
228. Chen ZM, Sandercock P, Pan HC, et al. Indications for early aspirin use in acute ischemic stroke : A combined analysis of 40 000 randomized patients from the chinese acute stroke trial and the international stroke trial. On behalf of the CAST and IST collaborative groups. Stroke 2000;**31**(6):1240-9.

229. Cheung WY, Shi Q, O'Connell M, et al. The predictive and prognostic value of sex in early-stage colon cancer: a pooled analysis of 33,345 patients from the ACCENT database. Clin Colorectal Cancer 2013;**12**(3):179-87.
230. Chiappini E, Galli L, Giaquinto C, et al. Use of combination neonatal prophylaxis for the prevention of mother-to-child transmission of HIV infection in European high-risk infants. AIDS 2013;**27**(6):991-1000.
231. Chierakul W, Anunnatsiri S, Short JM, et al. Two randomized controlled trials of ceftazidime alone versus ceftazidime in combination with trimethoprim-sulfamethoxazole for the treatment of severe melioidosis. Clin Infect Dis 2005;**41**(8):1105-13.
232. Childhood Acute Lymphoblastic Leukaemia Collaborative Group. Beneficial and harmful effects of anthracyclines in the treatment of childhood acute lymphoblastic leukaemia: a systematic review and meta-analysis. Br J Haematol 2009;**145**(3):376-88.
233. Childhood ALL Collaborative Group. Duration and intensity of maintenance chemotherapy in acute lymphoblastic leukaemia: overview of 42 trials involving 12 000 randomised children. . Lancet 1996;**347**(9018):1783-8.
234. Cho JK, Woo S-H, Park J, et al. Primary squamous cell carcinomas in the thyroid gland: an individual participant data meta-analysis. Cancer Medicine 2014;**3**(5):1396-403.
235. Cholesterol Treatment Trialists Collaboration, Baigent C, Blackwell L, et al. Efficacy and safety of more intensive lowering of LDL cholesterol: a meta-analysis of data from 170,000 participants in 26 randomised trials. Lancet 2010;**376**(9753):1670-81.
236. Cholesterol Treatment Trialists Collaborators, Mihaylova B, Emberson J, et al. The effects of lowering LDL cholesterol with statin therapy in people at low risk of vascular disease: meta-analysis of individual data from 27 randomised trials. Lancet 2012;**380**(9841):581-90.
237. Chowdhury N, Sapru S. Association of prot1ein translation and extracellular matrix gene sets with breast cancer metastasis: Findings uncovered on analysis of multiple publicly available datasets using individual patient data approach. PLoS One 2015;**10**(6).
238. Choy Pui Yee G, Bissett Ian P, Docherty James G, et al. Stapled versus handsewn methods for ileocolic anastomoses. Cochrane Database Syst Rev 2007.
239. Chronic Myeloid Leukemia Trialists' Collaborative Group. Interferon alfa versus chemotherapy for chronic myeloid leukemia: a meta-analysis of seven randomized trials. J Natl Cancer Inst 1997;**89**(21):1616-20.
240. Chronic myeloid leukemia trialists' collaborative group. Hydroxyurea versus busulphan for chronic myeloid leukaemia: an individual patient data meta-analysis of three randomized trials. . Br J Haematol 2000;**110**(3):573-6.
241. Chua DT, Ma J, Sham JS, et al. Long-term survival after cisplatin-based induction chemotherapy and radiotherapy for nasopharyngeal carcinoma: a pooled data analysis of two phase III trials. J Clin Oncol 2005;**23**(6):1118-24.
242. Cil AP, Bang H, Oktay K. Age-specific probability of live birth with oocyte cryopreservation: an individual patient data meta-analysis. Fertil Steril 2013;**100**(2):492-9.e3.
243. Clahsen PC, van de Velde CJ, Goldhirsch A, et al. Overview of randomized perioperative polychemotherapy trials in women with early-stage breast cancer. J Clin Oncol 1997;**15**(7):2526-35.
244. Clark IA, Mackay CE, Holmes EA. Low emotional response to traumatic footage is associated with an absence of analogue flashbacks: An individual participant data meta-analysis of 16 trauma film paradigm experiments. Cognition and Emotion 2015;**29**(4):702-13.
245. Clarke M, Gaynon P, Hann I, et al. CNS-directed therapy for childhood acute lymphoblastic leukemia: Childhood ALL Collaborative Group overview of 43 randomized trials. J Clin Oncol 2003;**21**(9):1798-809.
246. Clarke R, Halsey J, Lewington S, et al. Effects of lowering homocysteine levels with B vitamins on cardiovascular disease, cancer, and cause-specific mortality: Meta-analysis of 8 randomized trials involving 37 485 individuals. Arch Intern Med 2010;**170**(18):1622-31.
247. Clauser C, Nieri M, Franceschi D, et al. Evidence-based mucogingival therapy. Part 2: Ordinary and individual patient data meta-analyses of surgical treatment of recession using complete root coverage as the outcome variable. J Periodontol 2003;**74**(5):741-56.
248. Clauss W, Dinger J, Meissner C. Renal tolerance of iotrolan 280—a meta-analysis of 14 double-blind studies. Eur Radiol 1995;**5**(2):S79-S84.
249. Cleland JG, Abraham WT, Linde C, et al. An individual patient meta-analysis of five randomized trials assessing the effects of cardiac resynchronization therapy on morbidity and mortality in patients with symptomatic heart failure. Eur Heart J 2013;**34**(46):3547-56.
250. Clemens A, Fraessdorf M, Friedman J. Cardiovascular outcomes during treatment with dabigatran: comprehensive analysis of individual subject data by treatment. Vascular Health and Risk Management 2013;**9**:599-615.

251. Cohen EEW, Halpern AB, Kasza K, et al. Factors associated with clinical benefit from epidermal growth factor receptor inhibitors in recurrent and metastatic squamous cell carcinoma of the head and neck. Oral Oncol 2009;**45**(10):e155-60.
252. Collaborative Group on Epidemiological Studies of Ovarian Cancer, Beral V, Gaitskell K, et al. Ovarian cancer and smoking: individual participant meta-analysis including 28,114 women with ovarian cancer from 51 epidemiological studies. Lancet Oncol 2012;**13**(9):946-56.
253. Collet T-H, Bauer DC, Cappola AR, et al. Thyroid antibody status, subclinical hypothyroidism, and the risk of coronary heart disease: an individual participant data analysis. J Clin Endocrinol Metab 2014;**99**(9):3353-62.
254. Collette L, Burzykowski T, Carroll KJ, et al. Is prostate-specific antigen a valid surrogate end point for survival in hormonally treated patients with metastatic prostate cancer? Joint research of the European Organisation for Research and treatment of cancer, the Limburgs Universitair Centrum, and AstraZeneca Pharmaceuticals. J Clin Oncol 2005;**23**(25):6139-48.
255. Collins M, Wilhelm M, Conyers R, et al. Benefits and adverse events in younger versus older patients receiving neoadjuvant chemotherapy for osteosarcoma: findings from a meta-analysis. J Clin Oncol 2013;**31**(18):2303-12.
256. Colorectal Cancer Collaborative G. Adjuvant radiotherapy for rectal cancer: a systematic overview of 8,507 patients from 22 randomised trials.[see comment]. Lancet 2001;**358**(9290):1291-304.
257. Connolly SJ, Hallstrom AP, Cappato R, et al. Meta-analysis of the implantable cardioverter defibrillator secondary prevention trials. AVID, CASH and CIDS studies. Antiarrhythmics vs Implantable Defibrillator study. Cardiac Arrest Study Hamburg . Canadian Implantable Defibrillator Study. Eur Heart J 2000;**21**(24):2071-8.
258. Conway DI, Brenner DR, McMahon AD, et al. Estimating and explaining the effect of education and income on head and neck cancer risk: INHANCE consortium pooled analysis of 31 case-control studies from 27 countries. Int J Cancer 2015;**136**(5):1125-39.
259. Cook MB, Guénel P, Gapstur SM, et al. Tobacco and alcohol in relation to male breast cancer: An analysis of the male breast cancer pooling project consortium. Cancer Epidemiology Biomarkers and Prevention 2015;**24**(3):520-31.
260. Cools F, Askie LM, Offringa M, et al. Elective high-frequency oscillatory versus conventional ventilation in preterm infants: a systematic review and meta-analysis of individual patients' data. Lancet 2010;**375**(9731):2082-91.
261. Cooper BC, Mullins PR, Jones MR, et al. Clinical efficacy of roxithromycin in the treatment of adults with upper and lower respiratory tract infection due to Haemophilus influenzae: A meta-analysis of 12 clinical studies. Drug Investigation 1994;**7**(6):299-314.
262. Cooper C, Kanters S, Klein M, et al. Liver transplant outcomes in HIV-infected patients: a systematic review and meta-analysis with synthetic cohort. AIDS 2011;**25**(6):777-86.
263. Cooper ME, Perkovic V, McGill JB, et al. Kidney Disease End Points in a Pooled Analysis of Individual Patient-Level Data From a Large Clinical Trials Program of the Dipeptidyl Peptidase 4 Inhibitor Linagliptin in Type 2 Diabetes. Am J Kidney Dis 2015.
264. Cope S, Zhang J, Williams J, et al. Efficacy of once-daily indacaterol 75 mug relative to alternative bronchodilators in COPD: a study level and a patient level network meta-analysis. BMC Pulm Med 2012;**12**:29.
265. Coresh J, Turin TC, Matsushita K, et al. Decline in estimated glomerular filtration rate and subsequent risk of end-stage renal disease and mortality. JAMA 2014;**311**(24):2518-31.
266. Cornu C, Boutitie F, Candelise L, et al. Streptokinase in acute ischemic stroke: an individual patient data meta-analysis : The Thrombolysis in Acute Stroke Pooling Project. Stroke 2000;**31**(7):1555-60.
267. Costantino G, Casazza G, Reed M, et al. Syncope risk stratification tools vs clinical judgment: an individual patient data meta-analysis. Am J Med 2014;**127**(11):1126.e13-25.
268. Coutinho T, Goel K, Correa de Sa D, et al. Central obesity and survival in subjects with coronary artery disease: a systematic review of the literature and collaborative analysis with individual subject data. J Am Coll Cardiol 2011;**57**(19):1877-86.
269. Coutsoudis A, Dabis F, Fawzi W, et al. Late postnatal transmission of HIV-1 in breast-fed children: an individual patient data meta-analysis.[see comment]. J Infect Dis 2004;**189**(12):2154-66.
270. Couture F, Turner AR, Melosky B, et al. Prior red blood cell transfusions in cancer patients increase the risk of subsequent transfusions with or without recombinant human erythropoietin management. Oncologist 2005;**10**(1):63-71.
271. Cowling BJ, Shaw JEH, Hutton JL, et al. New statistical method for analyzing time to first seizure: Example using data comparing carbamazepine and valproate monotherapy. Epilepsia 2007;**48**(6):1173-78.

272. Coxib and traditional NSAID Trialists' (CNT) Collaboration, Bhala N, Emberson J, et al. Vascular and upper gastrointestinal effects of non-steroidal anti-inflammatory drugs: meta-analyses of individual participant data from randomised trials. Lancet 2013;**382**(9894):769-79.
273. Craig JV, Lancaster GA, Taylor S, et al. Infrared ear thermometry compared with rectal thermometry in children: a systematic review.[see comment]. [Review] [48 refs]. Lancet 2002;**360**(9333):603-9.
274. Craig LE, Bernhardt J, Langhorne P, et al. Early mobilization after stroke: an example of an individual patient data meta-analysis of a complex intervention. Stroke 2010;**41**(11):2632-6.
275. Cranney A, Wells GA, Yetisir E, et al. Ibandronate for the prevention of nonvertebral fractures: A pooled analysis of individual patient data. Osteoporos Int 2009;**20**(2):291-97.
276. Crawford MR, Bartlett DJ, Coughlin SR, et al. The effect of continuous positive airway pressure usage on sleepiness in obstructive sleep apnoea: real effects or expectation of benefit? Thorax 2012;**67**(10):920-4.
277. Crowe FL, Appleby PN, Travis RC, et al. Circulating fatty acids and prostate cancer risk: individual participant meta-analysis of prospective studies. J Natl Cancer Inst 2014;**106**(9):dju240.
278. Crump M, Sawka CA, DeBoer G, et al. An individual patient-based meta-analysis of tamoxifen versus ovarian ablation as first line endocrine therapy for premenopausal women with metastatic breast cancer. Breast Cancer Res Treat 1997;**44**(3):201-10.
279. Cuijpers P, Weitz E, Twisk J, et al. Gender as predictor and moderator of outcome in cognitive behavior therapy and pharmacotherapy for adult depression: an "individual patient data" meta-analysis. Depress Anxiety 2014;**31**(11):941-51.
280. Cuzick J, Clavel C, Petry K-U, et al. Overview of the European and North American studies on HPV testing in primary cervical cancer screening. Int J Cancer 2006;**119**(5):1095-101.
281. Cuzick J, Sestak I, Bonanni B, et al. Selective oestrogen receptor modulators in prevention of breast cancer: an updated meta-analysis of individual participant data. Lancet 2013;**381**(9880):1827-34.
282. Cuzick J, Stewart H, Peto R, et al. Overview of randomized trials comparing radical mastectomy without radiotherapy against simple mastectomy with radiotherapy in breast cancer. Cancer Treat Rep 1987;**71**(1):7-14.
283. Czernichow S, Kengne AP, Stamatakis E, et al. Body mass index, waist circumference and waist-hip ratio: which is the better discriminator of cardiovascular disease mortality risk?: evidence from an individual-participant meta-analysis of 82 864 participants from nine cohort studies. Obes Rev 2011;**12**(9):680-7.
284. Czobor P, Van Dorn RA, Citrome L, et al. Treatment adherence in schizophrenia: A patient-level meta-analysis of combined CATIE and EUFEST studies. Eur Neuropsychopharmacol 2015.
285. Daemen J, Boersma E, Flather M, et al. Long-term safety and efficacy of percutaneous coronary intervention with stenting and coronary artery bypass surgery for multivessel coronary artery disease: a meta-analysis with 5-year patient-level data from the ARTS, ERACI-II, MASS-II, and SoS trials. Circulation 2008;**118**(11):1146-54.
286. Dafni U, Grimani I, Xyrafas A, et al. Fifteen-year trends in metastatic breast cancer survival in Greece. Breast Cancer Res Treat 2010;**119**(3):621-31.
287. Dahlof CG, Pascual J, Dodick DW, et al. Efficacy, speed of action and tolerability of almotriptan in the acute treatment of migraine: Pooled individual patient data from four randomized, double-blind, placebo-controlled clinical trials. Cephalalgia 2006;**26**(4):400-08.
288. D'Amico R, Pifferi S, Leonetti C, et al. Effectiveness of antibiotic prophylaxis in critically ill adult patients: systematic review of randomised controlled trials.[see comment]. BMJ 1998;**316**(7140):1275-85.
289. Damman P, Wallentin L, Fox KAA, et al. Long-term cardiovascular mortality after procedure-related or spontaneous myocardial infarction in patients with non-ST-segment elevation acute coronary syndrome: a collaborative analysis of individual patient data from the FRISC II, ICTUS, and RITA-3 trials (FIR). Circulation 2012;**125**(4):568-76.
290. Danesh J, Lewington S, Thompson SG, et al. Plasma fibrinogen level and the risk of major cardiovascular diseases and nonvascular mortality: An individual participant meta-analysis. J Am Med Assoc 2005;**294**(14):1799-809.
291. Dangas GD, Serruys PW, Kereiakes DJ, et al. Meta-analysis of everolimus-eluting versus paclitaxel-eluting stents in coronary artery disease: final 3-year results of the SPIRIT clinical trials program (Clinical Evaluation of the Xience V Everolimus Eluting Coronary Stent System in the Treatment of Patients With De Novo Native Coronary Artery Lesions). JACC Cardiovasc Interv 2013;**6**(9):914-22.
292. Dangour AD, Watson L, Cumming O, et al. Interventions to improve water quality and supply, sanitation and hygiene practices, and their effects on the nutritional status of children. Cochrane Database Syst Rev 2013;**8**:CD009382.

293. Daniels JP, Middleton L, Xiong T, et al. Individual patient data meta-analysis of randomized evidence to assess the effectiveness of laparoscopic uterosacral nerve ablation in chronic pelvic pain. Hum Reprod Update 2010;**16**(6):568-76.
294. Darby S, Hill D, Deo H, et al. Residential radon and lung cancer--detailed results of a collaborative analysis of individual data on 7148 persons with lung cancer and 14,208 persons without lung cancer from 13 epidemiologic studies in Europe. Scand J Work Environ Health 2006;**32 Suppl 1**:1-83.
295. Darbyshire J, Foulkes M, Peto R, et al. Zidovudine (AZT) versus AZT plus didanosine (ddI) versus AZT plus zalcitabine (ddC) in HIV infected adults.[update in Cochrane Database Syst Rev. 2000;(3):CD002038; PMID: 10908523]. [Review] [26 refs]. Cochrane Database Syst Rev 2000(2):CD002038.
296. Darbyshire J, Foulkes M, Peto R, et al. Immediate versus deferred zidovudine (AZT) in asymptomatic or mildly symptomatic HIV infected adults.[update of Cochrane Database Syst Rev. 2000;(2):CD002039; PMID: 10796852]. [Review] [30 refs]. Cochrane Database Syst Rev 2000(3):CD002039.
297. Davalos A, Castillo J, Alvarez-Sabin J, et al. Oral citicoline in acute ischemic stroke: an individual patient data pooling analysis of clinical trials. Stroke 2002;**33**(12):2850-7.
298. Davidson JA, Liebl A, Christiansen JS, et al. Risk for nocturnal hypoglycemia with biphasic insulin aspart 30 compared with biphasic human insulin 30 in adults with type 2 diabetes mellitus: a meta-analysis. Clin Ther 2009;**31**(8):1641-51.
299. Davis JM, Chen N. The effects of olanzapine on the 5 dimensions of schizophrenia derived by factor analysis: combined results of the North American and international trials. J Clin Psychiatry 2001;**62**(10):757-71.
300. Davison BA, Metra M, Cotter G, et al. Worsening heart failure following admission for acute heart failure. A pooled analysis of the PROTECT and RELAX-AHF Studies. JACC Heart Fail 2015;**3**(5):395-403.
301. Dawson SJ, Makretsov N, Blows FM, et al. BCL2 in breast cancer: a favourable prognostic marker across molecular subtypes and independent of adjuvant therapy received.[Erratum appears in Br J Cancer. 2010 Sep 28;103(7):1137]. Br J Cancer 2010;**103**(5):668-75.
302. Daya S, Gunby J. The effectiveness of allogeneic leukocyte immunization in unexplained primary recurrent spontaneous abortion. Am J Reprod Immunol 1994;**32**(4):294-302.
303. Daya S, Gunby J, Porter F, et al. Critical analysis of intravenous immunoglobulin therapy for recurrent miscarriage. Hum Reprod Update 1999;**5**(5):475-82.
304. de Backer Tine LM, Vander Stichele R, Lehert P, et al. Naftidrofuryl for intermittent claudication. Cochrane Database Syst Rev 2008(2).
305. de Backer TLM, Vander Stichele R, Lehert P, et al. Naftidrofuryl for intermittent claudication. Cochrane Database Syst Rev 2012;**12**:CD001368.
306. de Boer SPM, Barnes EH, Westerhout CM, et al. High-risk patients with ST-elevation myocardial infarction derive greatest absolute benefit from primary percutaneous coronary intervention: results from the Primary Coronary Angioplasty Trialist versus thrombolysis (PCAT)-2 collaboration. Am Heart J 2011;**161**(3):500-07.e1.
307. de Haas S, Delmar P, Bansal AT, et al. Genetic variability of VEGF pathway genes in six randomized phase III trials assessing the addition of bevacizumab to standard therapy. Angiogenesis 2014;**17**(4):909-20.
308. De Laet C, Kanis JA, Oden A, et al. Body mass index as a predictor of fracture risk: A meta-analysis. Osteoporos Int 2005;**16**(11):1330-38.
309. De LG, Gibson CM, Bellandi F, et al. Early glycoprotein IIb-IIIa inhibitors in primary angioplasty (EGYPT) cooperation: An individual patient data meta-analysis. Heart 2008;**94**(12):1548-58.
310. De Luca G, Bellandi F, Huber K, et al. Early glycoprotein IIb-IIIa inhibitors in primary angioplasty-abciximab long-term results (EGYPT-ALT) cooperation: individual patient's data meta-analysis. J Thromb Haemost 2011;**9**(12):2361-70.
311. De Luca G, Dirksen MT, Kelbæk H, et al. Paclitaxel-eluting versus bare metal stents in primary PCI: a pooled patient-level meta-analysis of randomized trials. J Thromb Thrombolysis 2014.
312. De Luca G, Dirksen MT, Spaulding C, et al. Drug-eluting vs bare-metal stents in primary angioplasty: a pooled patient-level meta-analysis of randomized trials. Arch Intern Med 2012;**172**(8):611-21; discussion 21-2.
313. De Luca G, Michael Gibson C, Bellandi F, et al. Benefits of pharmacological facilitation with glycoprotein IIb-IIIa inhibitors in diabetic patients undergoing primary angioplasty for STEMI. A subanalysis of the EGYPT cooperation. J Thromb Thrombolysis 2009;**28**(3):288-98.
314. De Luca G, Wirianta J, Lee JH, et al. Sirolimus-eluting versus paclitaxel-eluting stent in primary angioplasty: a pooled patient-level meta-analysis of randomized trials. J Thromb Thrombolysis 2014.
315. De Morton NA, Jones CT, Keating JL, et al. The effect of exercise on outcomes for hospitalised older acute medical patients: An individual patient data meta-analysis [1]. Age Ageing 2007;**36**(2):219-22.
316. de Oliveira IR, de Sena EP, Pereira EL, et al. Haloperidol blood levels and clinical outcome: a meta-analysis of studies relevant to testing the therapeutic window hypothesis. J Clin Pharm Ther 1996;**21**(4):229-36.

317. de Waha A, Dibra A, Kufner S, et al. Long-term outcome after sirolimus-eluting stents versus bare metal stents in patients with diabetes mellitus: a patient-level meta-analysis of randomized trials.[Erratum appears in Clin Res Cardiol. 2011 Sep;100(9):811-3]. Clin Res Cardiol 2011;**100**(7):561-70.
318. De Waha A, Stefanini GG, King LA, et al. Long-term outcomes of biodegradable polymer versus durable polymer drug-eluting stents in patients with diabetes a pooled analysis of individual patient data from 3 randomized trials. Int J Cardiol 2013;**168**(6):5162-66.
319. de Weerd M, Greving JP, Hedblad B, et al. Prevalence of asymptomatic carotid artery stenosis in the general population: an individual participant data meta-analysis. Stroke 2010;**41**(6):1294-7.
320. Degraeuwe PLJ, Beld MPA, Ashorn M, et al. Faecal calprotectin in suspected paediatric inflammatory bowel disease. J Pediatr Gastroenterol Nutr 2015;**60**(3):339-46.
321. Dehghan N, McKee MD, Nauth A, et al. Surgical fixation of vancouver type B1 periprosthetic femur fractures: A systematic review. J Orthop Trauma 2014.
322. del Canho R, Grosheide PM, Mazel JA, et al. Ten-year neonatal hepatitis B vaccination program, The Netherlands, 1982-1992: protective efficacy and long-term immunogenicity. Vaccine 1997;**15**(15):1624-30.
323. Delaney B, Ford AC, Forman D, et al. Initial management strategies for dyspepsia.[update of Cochrane Database Syst Rev. 2003;(2):CD001961; PMID: 12804417]. [Review] [90 refs]. Cochrane Database Syst Rev 2005(4):CD001961-.
324. Delfino A, Petrelli N. Meta-analysis of randomized trials testing the biochemical modulation of fluorouracil by methotrexate in metastatic colorectal cancer. J Clin Oncol 1994;**12**(5):960-69.
325. Demonty I, Ras RT, van der Knaap HCM, et al. The effect of plant sterols on serum triglyceride concentrations is dependent on baseline concentrations: a pooled analysis of 12 randomised controlled trials. Eur J Nutr 2013;**52**(1):153-60.
326. Den Ruijter HM, Peters SAE, Anderson TJ, et al. Common carotid intima-media thickness measurements in cardiovascular risk prediction: a meta-analysis.[Erratum appears in JAMA. 2013 Oct 23;310(16):1739]. JAMA 2012;**308**(8):796-803.
327. Denhaerynck K, Abraham I, Gourley G, et al. Validity testing of the Long-Term Medication Behavior Self-Efficacy Scale. J Nurs Meas 2003;**11**(3):267-82.
328. Depla AL, Scharloo-Karels CH, de Jong MAA, et al. Treatment and prognostic factors of radiation-associated angiosarcoma (RAAS) after primary breast cancer: A systematic review. Eur J Cancer 2014.
329. Desjardins PJ, Mehlisch DR, Chang DJ, et al. The time to onset and overall analgesic efficacy of rofecoxib 50 mg: a meta-analysis of 13 randomized clinical trials. Clin J Pain 2005;**21**(3):241-50.
330. Devaiah AK, Andreoli MT. Treatment of esthesioneuroblastoma: a 16-year meta-analysis of 361 patients. Laryngoscope 2009;**119**(7):1412-6.
331. Devaiah AK, Andreoli S. Postmaneuver restrictions in benign paroxysmal positional vertigo: an individual patient data meta-analysis. Otolaryngology - Head and Neck Surgery 2010;**142**(2):155-9.
332. Di Leo A, Desmedt C, Bartlett JMS, et al. HER2 and TOP2A as predictive markers for anthracycline-containing chemotherapy regimens as adjuvant treatment of breast cancer: a meta-analysis of individual patient data. Lancet Oncol 2011;**12**(12):1134-42.
333. Di Maio M, Chiodini P, Georgoulias V, et al. Meta-analysis of single-agent chemotherapy compared with combination chemotherapy as second-line treatment of advanced non-small-cell lung cancer. J Clin Oncol 2009;**27**(11):1836-43.
334. Di Maio M, Lama N, Morabito A, et al. Clinical assessment of patients with advanced non-small-cell lung cancer eligible for second-line chemotherapy: a prognostic score from individual data of nine randomised trials. Eur J Cancer 2010;**46**(4):735-43.
335. Di MM, Perrone F, Chiodini P, et al. Individual patient data meta-analysis of docetaxel administered once every 3 weeks compared with once every week second-line treatment of advanced non-small-cell lung cancer. J Clin Oncol 2007;**25**(11):1377-82.
336. Diener HC, Lees KR, Lyden P, et al. NXY-059 for the treatment of acute stroke: pooled analysis of the SAINT I and II Trials. Stroke 2008;**39**(6):1751-8.
337. DiGiuro G, Ignjatovic D, Brogger J, et al. How accurate are published recurrence rates after rectal prolapse surgery? A meta-analysis of individual patient data. Am J Surg 2006;**191**(6):773-78.
338. Diletti R, Garcia-Garcia HM, Bourantas C, et al. Impact of body mass index on long-term clinical outcomes after second-generation drug eluting stent implantation: Insights from the international global RESOLUTE program. Catheter Cardiovasc Interv 2015;**85**(6):952-58.
339. Dillon DG, Gurdasani D, Riha J, et al. Association of HIV and ART with cardiometabolic traits in sub-Saharan Africa: A systematic review and meta-analysis. Int J Epidemiol 2013;**42**(6):1754-71.

340. Ding J, Wai KL, McGeechan K, et al. Retinal vascular caliber and the development of hypertension: a meta-analysis of individual participant data. Journal of Hypertension 2014;**32**(2):207-15.
341. Dipart Group. Patient level pooled analysis of 68 500 patients from seven major vitamin D fracture trials in US and Europe. BMJ 2010;**340**:b5463.
342. Direct Thrombin Inhibitor Trialists' Collaborative G. Direct thrombin inhibitors in acute coronary syndromes: principal results of a meta-analysis based on individual patients' data.[see comment]. Lancet 2002;**359**(9303):294-302.
343. Dobson J, Whitley RJ, Pocock S, et al. Oseltamivir treatment for influenza in adults: a meta-analysis of randomised controlled trials. Lancet 2015;**385**(9979):1729-37.
344. Donadini MP, Ageno W, Antonucci E, et al. Prognostic significance of residual venous obstruction in patients with treated unprovoked deep vein thrombosis: A patient-level meta-analysis. Thromb Haemost 2013;**111**(1):172-79.
345. Douketis J, Tosetto A, Marcucci M, et al. Risk of recurrence after venous thromboembolism in men and women: patient level meta-analysis. BMJ 2011;**342**:d813.
346. Douketis J, Tosetto A, Marcucci M, et al. Patient-level meta-analysis: effect of measurement timing, threshold, and patient age on ability of D-dimer testing to assess recurrence risk after unprovoked venous thromboembolism. Ann Intern Med 2010;**153**(8):523-31.
347. Downing M, Lau F, Lesperance M, et al. Meta-analysis of survival prediction with Palliative Performance Scale. J Palliat Care 2007;**23**(4):245-52.
348. Dowsett M, Cuzick J, Ingle J, et al. Meta-analysis of breast cancer outcomes in adjuvant trials of aromatase inhibitors versus tamoxifen. J Clin Oncol 2010;**28**(3):509-18.
349. Draube A, Klein-Gonzalez N, Mattheus S, et al. Dendritic cell based tumor vaccination in prostate and renal cell cancer: a systematic review and meta-analysis. PLoS ONE [Electronic Resource] 2011;**6**(4):e18801.
350. Dubreuil M, Greger S, LaValley M, et al. Improvement in wrist pain with ultrasound-guided glucocorticoid injections: a meta-analysis of individual patient data. Semin Arthritis Rheum 2013;**42**(5):492-7.
351. Duparc S, Borghini-Fuhrer I, Craft CJ, et al. Safety and efficacy of pyronaridine-artesunate in uncomplicated acute malaria: an integrated analysis of individual patient data from six randomized clinical trials. Malaria Journal 2013;**12**:70.
352. Durier C, Launay O, Meiffredy V, et al. Clinical safety of HIV lipopeptides used as vaccines in healthy volunteers and HIV-infected adults. AIDS 2006;**20**(7):1039-49.
353. Early Breast Cancer Trialists' Collaborative Group. Ovarian ablation in early breast cancer: overview of the randomised trials. Lancet 1996;**348**(9036):1189-96.
354. Early Breast Cancer Trialists' Collaborative Group. Tamoxifen for early breast cancer: An overview of the randomised trials. Lancet 1998;**351**(9114):1451.
355. Early Breast Cancer Trialists' Collaborative Group. Polychemotherapy for early breast cancer: an overview of the randomised trials. Lancet 1998;**352**(9132):930-42.
356. Early Breast Cancer Trialists' Collaborative Group. Favourable and unfavourable effects on long-term survival of radiotherapy for early breast cancer: an overview of the randomised trials. . Lancet 2000;**355**(9217):1757-70.
357. Early Breast Cancer Trialists' Collaborative Group. Adjuvant chemotherapy in oestrogen-receptor-poor breast cancer: patient-level meta-analysis of randomised trials. Lancet 2008;**371**(9606):29-40.
358. Early Breast Cancer Trialists' Collaborative Group. Effect of radiotherapy after mastectomy and axillary surgery on 10-year recurrence and 20-year breast cancer mortality: meta-analysis of individual patient data for 8135 women in 22 randomised trials. Lancet 2014.
359. Early Breast Cancer Trialists' Collaborative Group, Correa C, McGale P, et al. Overview of the randomized trials of radiotherapy in ductal carcinoma in situ of the breast. J Natl Cancer Inst Monogr 2010;**2010**(41):162-77.
360. Early Breast Cancer Trialists' Collaborative Group, Darby S, McGale P, et al. Effect of radiotherapy after breast-conserving surgery on 10-year recurrence and 15-year breast cancer death: meta-analysis of individual patient data for 10,801 women in 17 randomised trials. Lancet 2011;**378**(9804):1707-16.
361. Early Breast Cancer Trialists' Collaborative Group, Davies C, Godwin J, et al. Relevance of breast cancer hormone receptors and other factors to the efficacy of adjuvant tamoxifen: patient-level meta-analysis of randomised trials. Lancet 2011;**378**(9793):771-84.
362. Early Breast Cancer Trialists' Collaborative Group, Peto R, Davies C, et al. Comparisons between different polychemotherapy regimens for early breast cancer: meta-analyses of long-term outcome among 100,000 women in 123 randomised trials. Lancet 2012;**379**(9814):432-44.

363. Early Breast Cancer Trialists' Collaborative Group (EBCTCG). Effects of chemotherapy and hormonal therapy for early breast cancer on recurrence and 15-year survival: an overview of the randomised trials. Lancet 2005;**365**(9472):1687-717.
364. Early Breast Cancer Trialists’ Collaborative Group (EBCTCG). Adjuvant bisphosphonate treatment in early breast cancer: meta-analyses of individual patient data from randomised trials. Lancet 2015.
365. EARLY BREAST CANCER TRIALISTS'COLLABORATIVE GROUP. Systemic treatment of early breast cancer by hormonal, cytotoxic, or immune therapy: 133 randomised trials involving 31 000 recurrences and 24 000 deaths among 75 000 women. Lancet 1992;**339**(8784):1-15.
366. Early Supported Discharge T. Services for reducing duration of hospital care for acute stroke patients. Cochrane Database Syst Rev 2005(2).
367. Ebbing M, Bonaa KH, Arnesen E, et al. Combined analyses and extended follow-up of two randomized controlled homocysteine-lowering B-vitamin trials. J Intern Med 2010;**268**(4):367-82.
368. Ebrahim S, Montoya L, Truong W, et al. Effectiveness of cognitive behavioral therapy for depression in patients receiving disability benefits: a systematic review and individual patient data meta-analysis. PLoS ONE [Electronic Resource] 2012;**7**(11):e50202.
369. Eckmann C, Montravers P, Bassetti M, et al. Efficacy of tigecycline for the treatment of complicated intra-abdominal infections in real-life clinical practice from five European observational studies. J Antimicrob Chemother 2013;**68 Suppl 2**:ii25-35.
370. Edwards JE, McQuay HJ, Moore RA. Combination analgesic efficacy: individual patient data meta-analysis of single-dose oral tramadol plus acetaminophen in acute postoperative pain.[see comment]. J Pain Symptom Manage 2002;**23**(2):121-30.
371. Edwards JE, Moore RA, McQuay HJ. Rofecoxib for dysmenorrhoea: Meta-analysis using individual patient data. BMC Womens Health 2004;**4**(5).
372. Egberts J, Brand R, Walti H, et al. Mortality, severe respiratory distress syndrome, and chronic lung disease of the newborn are reduced more after prophylactic than after therapeutic administration of the surfactant Curosurf. Pediatrics 1997;**100**(1):e4-e4.
373. Eggeling T, Regitz-Zagrosek V, Zimmermann A, et al. Baseline severity but not gender modulates quantified Crataegus extract effects in early heart failure--a pooled analysis of clinical trials. Phytomedicine 2011;**18**(14):1214-9.
374. Eikelboom JW, Quinlan DJ, O'Donnell M. Major bleeding, mortality, and efficacy of fondaparinux in venous thromboembolism prevention trials. Circulation 2009;**120**(20):2006-11.
375. Eikendal ALM, Groenewegen KA, Anderson TJ, et al. Common carotid intima-media thickness relates to cardiovascular events in adults aged <45 years. Hypertension 2015;**65**(4):707-13.
376. Eisen DP, Dean MM, Boermeester MA, et al. Low serum mannose-binding lectin level increases the risk of death due to pneumococcal infection. Clin Infect Dis 2008;**47**(4):510-6.
377. Elkayam LU, Koehler JL, Sheldon TJ, et al. The influence of atrial and ventricular pacing on the incidence of atrial fibrillation: a meta-analysis. Pacing Clin Electrophysiol 2011;**34**(12):1593-9.
378. Ellis AG, Reginster JY, Luo X, et al. Bazedoxifene versus Oral Bisphosphonates for the Prevention of Nonvertebral Fractures in Postmenopausal Women with Osteoporosis at Higher Risk of Fracture: A Network Meta-Analysis. Value Health 2014.
379. Ellis AG, Reginster J-Y, Luo X, et al. Indirect comparison of bazedoxifene vs oral bisphosphonates for the prevention of vertebral fractures in postmenopausal osteoporotic women. Curr Med Res Opin 2014;**30**(8):1617-26.
380. Ellis G, Mant J, Langhorne P, et al. Stroke liaison workers for stroke patients and carers: an individual patient data meta-analysis. Cochrane Database Syst Rev 2010(5):CD005066.
381. Elsayed EF, Sarnak MJ, Tighiouart H, et al. Waist-to-Hip Ratio, Body Mass Index, and Subsequent Kidney Disease and Death. Am J Kidney Dis 2008;**52**(1):29-38.
382. Elsayed EF, Tighiouart H, Griffith J, et al. Cardiovascular disease and subsequent kidney disease. Arch Intern Med 2007;**167**(11):1130-36.
383. Emberson J, Lees KR, Lyden P, et al. Effect of treatment delay, age, and stroke severity on the effects of intravenous thrombolysis with alteplase for acute ischaemic stroke: a meta-analysis of individual patient data from randomised trials. Lancet 2014;**384**(9958):1929-35.
384. Emberson JR, Kearney PM, Blackwell L, et al. Lack of effect of lowering LDL cholesterol on cancer: meta-analysis of individual data from 175,000 people in 27 randomised trials of statin therapy. PLoS One 2012;**7**(1):e29849.
385. Emerging Risk Factors Collaboration. Adult height and the risk of cause-specific death and vascular morbidity in 1 million people: individual participant meta-analysis. Int J Epidemiol 2012;**41**(5):1419-33.

386. Emerging Risk Factors Collaboration, Di Angelantonio E, Gao P, et al. Glycated hemoglobin measurement and prediction of cardiovascular disease. JAMA 2014;**311**(12):1225-33.
387. Emerging Risk Factors Collaboration, Di Angelantonio E, Kaptoge S, et al. Association of Cardiometabolic Multimorbidity With Mortality. JAMA 2015;**314**(1):52-60.
388. Emerging Risk Factors Collaboration, Seshasai SRK, Kaptoge S, et al. Diabetes mellitus, fasting glucose, and risk of cause-specific death.[Erratum appears in N Engl J Med. 2011 Mar 31;364(13):1281]. N Engl J Med 2011;**364**(9):829-41.
389. Empana JP, Tafflet M, Escolano S, et al. Predicting CHD risk in France: a pooled analysis of the D.E.S.I.R., Three City, PRIME, and SU.VI.MAX studies. Eur J Cardiovasc Prev Rehabil 2011;**18**(2):175-85.
390. Endogenous Hormones and Breast Cancer Collaborative Group, Key TJ, Appleby PN, et al. Sex hormones and risk of breast cancer in premenopausal women: a collaborative reanalysis of individual participant data from seven prospective studies. Lancet Oncol 2013;**14**(10):1009-19.
391. Engel LS, Taioli E, Pfeiffer R, et al. Pooled analysis and meta-analysis of glutathione S-transferase M1 and bladder cancer: a HuGE review. Am J Epidemiol 2002;**156**(2):95-109.
392. Entsuah AR, Huang H, Thase ME. Response and remission rates in different subpopulations with major depressive disorder administered venlafaxine, selective serotonin reuptake inhibitors, or placebo. J Clin Psychiatry 2001;**62**(11):869-77.
393. Ervasti J, Kivimäki M, Dray-Spira R, et al. Psychosocial factors associated with work disability in men and women with diabetes: A pooled analysis of three occupational cohort studies. Diabet Med 2015.
394. Escherich G, Richards S, Stork LC, et al. Meta-analysis of randomised trials comparing thiopurines in childhood acute lymphoblastic leukaemia. Leukemia 2011;**25**(6):953-9.
395. EU Hernia Trialists Collaboration. Repair of groin hernia with synthetic mesh: meta-analysis of randomized controlled trials. Ann Surg 2002;**235**(3):322-32.
396. Fabbri A, Magrini N, Bianchi G, et al. Overview of randomized clinical trials of oral branched-chain amino acid treatment in chronic hepatic encephalopathy. JPEN J Parenter Enteral Nutr 1996;**20**(2):159-64.
397. Fagard RH, Celis H, Thijs L, et al. Daytime and nighttime blood pressure as predictors of death and cause-specific cardiovascular events in hypertension. Hypertension 2008;**51**(1):55-61.
398. Falagas ME, Manta KG, Ntziora F, et al. Linezolid for the treatment of patients with endocarditis: A systematic review of the published evidence. J Antimicrob Chemother 2006;**58**(2):273-80.
399. Fallah A, Guyatt GH, Snead OC, 3rd, et al. Predictors of seizure outcomes in children with tuberous sclerosis complex and intractable epilepsy undergoing resective epilepsy surgery: an individual participant data meta-analysis. PLoS ONE [Electronic Resource] 2013;**8**(2):e53565.
400. Farmer AJ, Perera R, Ward A, et al. Meta-analysis of individual patient data in randomised trials of self monitoring of blood glucose in people with non-insulin treated type 2 diabetes. BMJ 2012;**344**:e486.
401. Fayad ZY, Semaan E, Fahoum B, et al. Aortic mural thrombus in the normal or minimally atherosclerotic aorta. Ann Vasc Surg 2013;**27**(3):282-90.
402. Fayers PM, Palumbo A, Hulin C, et al. Thalidomide for previously untreated elderly patients with multiple myeloma: meta-analysis of 1685 individual patient data from 6 randomized clinical trials. Blood 2011;**118**(5):1239-47.
403. Fearon P, Langhorne P, Early Supported Discharge T. Services for reducing duration of hospital care for acute stroke patients. Cochrane Database Syst Rev 2012;**9**:CD000443.
404. Feigin V, Parag V, Lawes CMM, et al. Smoking and elevated blood pressure are the most important risk factors for subarachnoid hemorrhage in the Asia-Pacific region: An overview of 26 cohorts involving 306 620 participants. Stroke 2005;**36**(7):1360-65.
405. Ferrari MD, Goadsby PJ, Roon KI, et al. Triptans (serotonin, 5-HT1B/1D agonists) in migraine: detailed results and methods of a meta-analysis of 53 trials. Cephalalgia 2002;**22**(8):633-58.
406. Ferrari MD, Roon KI, Lipton RB, et al. Oral triptans (serotonin 5-HT(1B/1D) agonists) in acute migraine treatment: a meta-analysis of 53 trials. Lancet 2001;**358**(9294):1668-75.
407. Ferreira ML, Ferreira PH, Henschke N, et al. Age does not modify the effects of treatment on pain in patients with low back pain: Secondary analyses of randomized clinical trials. European Journal of Pain (United Kingdom) 2014;**18**(7):932-38.
408. Feuer D, Broadley K. Systematic review and meta-analysis of corticosteroids for the resolution of malignant bowel obstruction in advanced gynaecological and gastrointestinal cancers. Ann Oncol 1999;**10**(9):1035-41.
409. Feuer DJ, Broadley KE. Corticosteroids for the resolution of malignant bowel obstruction in advanced gynaecological and gastrointestinal cancer. [Review] [10 refs]. Cochrane Database Syst Rev 2000(2):CD001219.

410. Fillmore KM, Golding JM, Leino EV, et al. Relationships of measures of alcohol consumption with alcohol-related problems in multiple studies: a research synthesis from the collaborative alcohol-related longitudinal project. Addiction 1994;**89**(9):1143-56.
411. Filmann N, Rey J, Schneeweiss S, et al. Prevalence of anemia in inflammatory bowel diseases in european countries: a systematic review and individual patient data meta-analysis. Inflamm Bowel Dis 2014;**20**(5):936-45.
412. Fisher WD, Eriksson BI, Bauer KA, et al. Rivaroxaban for thromboprophylaxis after orthopaedic surgery: pooled analysis of two studies. Thromb Haemost 2007;**97**(6):931-7.
413. Fizazi K, Le MA, Hudes G, et al. Addition of estramustine to chemotherapy and survival of patients with castration-refractory prostate cancer: a meta-analysis of individual patient data. Lancet Oncol 2007;**8**(11):994-1000.
414. Flather MD, Yusuf S, Kober L, et al. Long-term ACE-inhibitor therapy in patients with heart failure or left-ventricular dysfunction: a systematic overview of data from individual patients. ACE-Inhibitor Myocardial Infarction Collaborative Group.[see comment]. Lancet 2000;**355**(9215):1575-81.
415. Flint A, Gregersen NT, Gluud LL, et al. Associations between postprandial insulin and blood glucose responses, appetite sensations and energy intake in normal weight and overweight individuals: A meta-analysis of test meal studies. Br J Nutr 2007;**98**(1):17-25.
416. Flossmann E, Rothwell PM. Prognosis of vertebrobasilar transient ischaemic attack and minor stroke. Brain 2003;**126**(Pt 9):1940-54.
417. Flynn JT, Schiffman J, Feuer W, et al. The therapy of amblyopia: an analysis of the results of amblyopia therapy utilizing the pooled data of published studies. Trans Am Ophthalmol Soc 1998;**96**:431-53.
418. Foerster BR, Dwamena BA, Petrou M, et al. Diagnostic accuracy of diffusion tensor imaging in amyotrophic lateral sclerosis: a systematic review and individual patient data meta-analysis. Acad Radiol 2013;**20**(9):1099-106.
419. Fokkema M, Vrijenhoek JEP, Den Ruijter HM, et al. Stenting versus endarterectomy for restenosis following prior ipsilateral carotid endarterectomy: an individual patient data meta-analysis. Ann Surg 2015;**261**(3):598-604.
420. Fokkema ML, van der Meer P, Rao SV, et al. Safety and clinical outcome of erythropoiesis-stimulating agents in patients with ST-elevation myocardial infarction: a meta-analysis of individual patient data. Am Heart J 2014;**168**(3):354-62.e2.
421. Fonseca V, Davidson J, Home P, et al. Starting insulin therapy with basal insulin analog or premix insulin analog in T2DM: a pooled analysis of treat-to-target trials. Curr Med Res Opin 2010;**26**(7):1621-8.
422. Ford AC, Moayyedi P, Jarbol DE, et al. Meta-analysis: Helicobacter pylori 'test and treat' compared with empirical acid suppression for managing dyspepsia. Aliment Pharmacol Ther 2008;**28**(5):534-44.
423. Ford AC, Qume M, Moayyedi P, et al. Helicobacter pylori "test and treat" or endoscopy for managing dyspepsia: An individual patient data meta-analysis. Gastroenterology 2005;**128**(7):1838-44.
424. Forrester LT, Maayan N, Orrell M, et al. Aromatherapy for dementia. Cochrane Database Syst Rev 2014(2).
425. Foster NR, Renfro LA, Schild SE, et al. Multitrial Evaluation of Progression-Free Survival as a Surrogate End Point for Overall Survival in First-Line Extensive-Stage Small-Cell Lung Cancer. J Thorac Oncol 2015;**10**(7):1099-106.
426. Fournier JC, DeRubeis RJ, Hollon SD, et al. Antidepressant drug effects and depression severity: a patient-level meta-analysis. JAMA 2010;**303**(1):47-53.
427. Fowkes G, Fowkes FGR, Murray GD, et al. Ankle brachial index combined with Framingham risk score to predict cardiovascular events and mortality: A meta-analysis. JAMA 2008;**300**(2):197-208.
428. Fox K, Komajda M, Ford I, et al. Effect of ivabradine in patients with left-ventricular systolic dysfunction: a pooled analysis of individual patient data from the BEAUTIFUL and SHIFT trials. Eur Heart J 2013;**34**(29):2263-70.
429. Franchini M, Mengoli C, Lippi G, et al. Immune tolerance with rituximab in congenital haemophilia with inhibitors: a systematic literature review based on individual patients' analysis. [Review] [39 refs]. Haemophilia 2008;**14**(5):903-12.
430. Francis HP, Wade DT, Turner-Stokes L, et al. Does reducing spasticity translate into functional benefit? An exploratory meta-analysis. J Neurol Neurosurg Psychiatry 2004;**75**(11):1547-51.
431. Franklin J, Paus Marcus D, Pluetschow A, et al. Chemotherapy, radiotherapy and combined modality for Hodgkin's disease, with emphasis on second cancer risk. Cochrane Database Syst Rev 2005(4).
432. Fransson EI, Heikkila K, Nyberg ST, et al. Job strain as a risk factor for leisure-time physical inactivity: an individual-participant meta-analysis of up to 170,000 men and women: the IPD-Work Consortium. Am J Epidemiol 2012;**176**(12):1078-89.

433. Fransson EI, Nyberg ST, Heikkila K, et al. Comparison of alternative versions of the job demand-control scales in 17 European cohort studies: the IPD-Work consortium. BMC Public Health 2012;**12**:62.
434. Fransson EI, Nyberg ST, Heikkila K, et al. Job strain and the risk of stroke: an individual-participant data meta-analysis. Stroke 2015;**46**(2):557-9.
435. Franzosi M, Santoro E, Zuanetti G, et al. Indications for ACE inhibitors in the early treatment of acute myocardial infarction-Systematic overview of individual data from 100,000 patients in randomized trials. Circulation 1998;**97**(22):2202-12.
436. Freeman R, Emir B, Parsons B. Predictors of placebo response in peripheral neuropathic pain: Insights from pregabalin clinical trials. J Pain Res 2015;**8**:257-68.
437. Fried LF, Orchard TJ, Kasiske BL. Effect of lipid reduction on the progression of renal disease: a meta-analysis. Kidney Int 2001;**59**(1):260-9.
438. Frosch K-H, Preiss A, Heider S, et al. Primary ligament sutures as a treatment option of knee dislocations: a meta-analysis. Knee Surgery, Sports Traumatology, Arthroscopy 2013;**21**(7):1502-9.
439. Fruh M, Rolland E, Pignon J-P, et al. Pooled analysis of the effect of age on adjuvant cisplatin-based chemotherapy for completely resected non-small-cell lung cancer. J Clin Oncol 2008;**26**(21):3573-81.
440. Fu R, Selph S, McDonagh M, et al. Effectiveness and harms of recombinant human bone morphogenetic protein-2 in spine fusion: a systematic review and meta-analysis. Ann Intern Med 2013;**158**(12):890-902.
441. Furst DE, Anderson W. Differential effects of diclofenac and aspirin on serum glutamic oxaloacetic transaminase elevations in patients with rheumatoid arthritis and osteoarthritis. Arthritis Rheum 1993;**36**(6):804-10.
442. Gage BF, van Walraven C, Pearce L, et al. Selecting patients with atrial fibrillation for anticoagulation: stroke risk stratification in patients taking aspirin. Circulation 2004;**110**(16):2287-92.
443. Galiatsatos P, Shrier I, Lamoureux E, et al. Meta-analysis of outcome of cytomegalovirus colitis in immunocompetent hosts. [Review] [41 refs]. Dig Dis Sci 2005;**50**(4):609-16.
444. Galsky MD, Krege S, Lin CC, et al. Cisplatin-based combination chemotherapy in septuagenarians with metastatic urothelial cancer. Urologic Oncology: Seminars and Original Investigations 2014;**32**(1):30.e15-30.e21.
445. Gamble C, Williamson PR, Chadwick DW, et al. A meta-analysis of individual patient responses to lamotrigine or carbamazepine monotherapy. Neurology 2006;**66**(9):1310-17.
446. Gans SL, Atema JJ, Stoker J, et al. C-reactive protein and white blood cell count as triage test between urgent and nonurgent conditions in 2961 patients with acute abdominal pain. Medicine 2015;**94**(9):e569.
447. Garcia-Berrocoso T, Giralt D, Bustamante A, et al. B-type natriuretic peptides and mortality after stroke: a systematic review and meta-analysis. Neurology 2013;**81**(23):1976-85.
448. Gardner MP, Lightman S, Sayer AA, et al. Dysregulation of the hypothalamic pituitary adrenal (HPA) axis and physical performance at older ages: an individual participant meta-analysis. Psychoneuroendocrinology 2013;**38**(1):40-9.
449. Gasowski J, Fagard RH, Staessen JA, et al. Pulsatile blood pressure component as predictor of mortality in hypertension: a meta-analysis of clinical trial control groups. Journal of Hypertension 2002;**20**(1):145-51.
450. Gasowski J, Wilkins A, Drzewoski J, et al. Short-term antihypertensive efficacy of perindopril according to clinical profile of 3,188 patients: A meta-analysis. Cardiol J 2010;**17**(3):259-66.
451. Gastric Group, Oba K, Paoletti X, et al. Role of chemotherapy for advanced/recurrent gastric cancer: an individual-patient-data meta-analysis. Eur J Cancer 2013;**49**(7):1565-77.
452. Gastric Group, Paoletti X, Oba K, et al. Benefit of adjuvant chemotherapy for resectable gastric cancer: a meta-analysis. JAMA 2010;**303**(17):1729-37.
453. Gauthier S, Proaño JV, Jia J, et al. Cerebrolysin in Mild-to-Moderate Alzheimer's Disease: A Meta-Analysis of Randomized Controlled Clinical Trials. Dement Geriatr Cogn Disord 2015;**39**(5/6):332-47.
454. Gaynor JW, Stopp C, Wypij D, et al. Neurodevelopmental outcomes after cardiac surgery in infancy. Pediatrics 2015;**135**(5):816-25.
455. Geddes JR, Calabrese JR, Goodwin GM. Lamotrigine for treatment of bipolar depression: independent meta-analysis and meta-regression of individual patient data from five randomised trials. [Review] [27 refs]. Br J Psychiatry 2009;**194**(1):4-9.
456. Geddes JR, Verdoux H, Takei N, et al. Schizophrenia and complications of pregnancy and labor: an individual patient data meta-analysis. Schizophr Bull 1999;**25**(3):413-23.
457. Geersing GJ, Zuithoff NPA, Kearon C, et al. Exclusion of deep vein thrombosis using the Wells rule in clinically important subgroups: individual patient data meta-analysis. BMJ 2014;**348**:g1340.
458. Gencer B, Collet T-H, Virgini V, et al. Subclinical thyroid dysfunction and cardiovascular outcomes among prospective cohort studies. Endocrine, Metabolic and Immune Disorders Drug Targets 2013;**13**(1):4-12.

459. Gencer B, Collet T-H, Virgini V, et al. Subclinical thyroid dysfunction and the risk of heart failure events: an individual participant data analysis from 6 prospective cohorts. Circulation 2012;**126**(9):1040-9.
460. Genders TSS, Steyerberg EW, Hunink MGM, et al. Prediction model to estimate presence of coronary artery disease: retrospective pooled analysis of existing cohorts. BMJ 2012;**344**:e3485.
461. Gennari A, Conte P, Rosso R, et al. Survival of metastatic breast carcinoma patients over a 20-year period: A retrospective analysis based on individual patient data from six consecutive studies. Cancer 2005;**104**(8):1742-50.
462. Getahun H, Kittikraisak W, Heilig CM, et al. Development of a standardized screening rule for tuberculosis in people living with HIV in resource-constrained settings: individual participant data meta-analysis of observational studies. PLoS Medicine / Public Library of Science 2011;**8**(1):e1000391.
463. Gevers TJG, Inthout J, Caroli A, et al. Young women with polycystic liver disease respond best to somatostatin analogues: a pooled analysis of individual patient data. Gastroenterology 2013;**145**(2):357-65.e1-2.
464. Ghio S, Temporelli PL, Marsan NA, et al. Prognostic implications of left ventricular dilation in patients with nonischemic heart failure: interactions with restrictive filling pattern and mitral regurgitation. Congest Heart Fail 2012;**18**(4):198-204.
465. Giallauria F, Vigorito C, Piepoli MF, et al. Effects of cardiac contractility modulation by non-excitatory electrical stimulation on exercise capacity and quality of life: an individual patient's data meta-analysis of randomized controlled trials. Int J Cardiol 2014;**175**(2):352-7.
466. Gibbons RD, Hur K, Brown CH, et al. Benefits from antidepressants: synthesis of 6-week patient-level outcomes from double-blind placebo-controlled randomized trials of fluoxetine and venlafaxine. Arch Gen Psychiatry 2012;**69**(6):572-9.
467. Gidal BE, Baltes E, Otoul C, et al. Effect of levetiracetam on the pharmacokinetics of adjunctive antiepileptic drugs: a pooled analysis of data from randomized clinical trials. Epilepsy Res 2005;**64**(1-2):1-11.
468. Giebel S, Thomas X, Hallbook H, et al. The prophylactic use of granulocyte-colony stimulating factor during remission induction is associated with increased leukaemia-free survival of adults with acute lymphoblastic leukaemia: a joint analysis of five randomised trials on behalf of the EWALL. Eur J Cancer 2012;**48**(3):360-7.
469. Gill PJ, Ashdown HF, Wang K, et al. Identification of children at risk of influenza-related complications in primary and ambulatory care: A systematic review and meta-analysis. Lancet Respir Med 2015;**3**(2):139-49.
470. Gill S, Loprinzi CL, Sargent DJ, et al. Pooled analysis of fluorouracil-based adjuvant therapy for stage II and III colon cancer: who benefits and by how much? J Clin Oncol 2004;**22**(10):1797-806.
471. Giorgino FL, Egan CG. Use of isoxsuprine hydrochloride as a tocolytic agent in the treatment of preterm labour: a systematic review of previous literature. Arzneimittelforschung 2010;**60**(7):415-20.
472. Giovanni B, Michelle E, Letizia C, et al. Thalidomide in myelofibrosis with myeloid metaplasia: a pooled-analysis of individual patient data from five studies. Leuk Lymphoma 2002;**43**(12):2301-7.
473. Gluud LL, Dam G, Borre M, et al. Oral branched-chain amino acids have a beneficial effect on manifestations of hepatic encephalopathy in a systematic review with meta-analyses of randomized controlled trials. J Nutr 2013;**143**(8):1263-8.
474. Gluud LL, Knop FK, Vilsboll T. Effects of lixisenatide on elevated liver transaminases: systematic review with individual patient data meta-analysis of randomised controlled trials on patients with type 2 diabetes. BMJ Open 2014;**4**(12):e005325.
475. Gnanenthiran SR, Adie S, Harris IA. Nonoperative versus operative treatment for thoracolumbar burst fractures without neurologic deficit: a meta-analysis. Clin Orthop Relat Res 2012;**470**(2):567-77.
476. Goetz CG, Wuu J, McDermott MP, et al. Placebo response in Parkinson's disease: Comparisons among 11 trials covering medical and surgical interventions. Mov Disord 2008;**23**(5):690-99.
477. Gomes M, Ribeiro I, Warsame M, et al. Rectal artemisinins for malaria: A review of efficacy and safety from individual patient data in clinical studies. BMC Infect Dis 2008;**8**(39):-.
478. Gonik NJ, Cheng J, Lesser M, et al. Patient selection in congenital pyriform aperture stenosis repair - 14 year experience and systematic review of literature. Int J Pediatr Otorhinolaryngol 2015;**79**(2):235-39.
479. Goodacre S, Stevens JW, Pandor A, et al. Prehospital noninvasive ventilation for acute respiratory failure: systematic review, network meta-analysis, and individual patient data meta-analysis. Acad Emerg Med 2014;**21**(9):960-70.
480. Gordon CE, Uhlig K, Lau J, et al. Interferon for hepatitis C virus in hemodialysis--an individual patient meta-analysis of factors associated with sustained virological response. Clinical Journal of The American Society of Nephrology: CJASN 2009;**4**(9):1449-58.
481. Gorman JD, David-Vaudey E, Pai M, et al. Particular HLA-DRB1 shared epitope genotypes are strongly associated with rheumatoid vasculitis. Arthritis Rheum 2004;**50**(11):3476-84.

482. Gorman JD, David-Vaudey E, Pai M, et al. Lack of association of the HLA-DRB1 shared epitope with rheumatoid nodules: an individual patient data meta-analysis of 3,272 Caucasian patients with rheumatoid arthritis. Arthritis Rheum 2004;**50**(3):753-62.
483. Gorman JD, Lum RF, Chen JJ, et al. Impact of shared epitope genotype and ethnicity on erosive disease: a meta-analysis of 3,240 rheumatoid arthritis patients. Arthritis Rheum 2004;**50**(2):400-12.
484. Gouw SC, Van Den Berg HM, Le CS, et al. Treatment characteristics and the risk of inhibitor development: A multicenter cohort study among previously untreated patients with severe hemophilia A. J Thromb Haemost 2007;**5**(7):1383-90.
485. Grant AM, Collaboration EHT. Laparoscopic versus open groin hernia repair: meta-analysis of randomised trials based on individual patient data. Hernia 2002;**6**(1):2-10.
486. Greb A, Bohlius J, Schiefer D, et al. High-dose chemotherapy with autologous stem cell transplantation in the first line treatment of aggressive Non-Hodgkin Lymphoma (NHL) in adults. Cochrane Database Syst Rev 2008(1):-.
487. Green SM, Roback MG, Krauss B, et al. Predictors of emesis and recovery agitation with emergency department ketamine sedation: an individual-patient data meta-analysis of 8,282 children. Ann Emerg Med 2009;**54**(2):171-80.e1-4.
488. Green SM, Roback MG, Krauss B, et al. Predictors of airway and respiratory adverse events with ketamine sedation in the emergency department: an individual-patient data meta-analysis of 8,282 children. Ann Emerg Med 2009;**54**(2):158-68.e1-4.
489. Greenland S, Sheppard AR, Kaune WT, et al. A pooled analysis of magnetic fields, wire codes, and childhood leukemia. Epidemiology 2000;**11**(6):624-34.
490. Gregson BA, Broderick JP, Auer LM, et al. Individual patient data subgroup meta-analysis of surgery for spontaneous supratentorial intracerebral hemorrhage.[Erratum appears in Stroke. 2013 Jul;44(7):e82]. Stroke 2012;**43**(6):1496-504.
491. Greving JP, Wermer MJH, Brown RD, Jr., et al. Development of the PHASES score for prediction of risk of rupture of intracranial aneurysms: a pooled analysis of six prospective cohort studies. Lancet Neurol 2014;**13**(1):59-66.
492. Griffith LE, Shannon HS, Wells RP, et al. Individual participant data meta-analysis of mechanical workplace risk factors and low back pain. Am J Public Health 2012;**102**(2):309-18.
493. Grines C, Patel A, Zijlstra F, et al. Primary coronary angioplasty compared with intravenous thrombolytic therapy for acute myocardial infarction: six-month follow up and analysis of individual patient data from randomized trials. [Review] [35 refs]. Am Heart J 2003;**145**(1):47-57.
494. Groen RJM, Middel B, Meilof JF, et al. Operative treatment of anterior thoracic spinal cord herniation: three new cases and an individual patient data meta-analysis of 126 case reports. Neurosurgery 2009;**64**(3 Suppl):ons145-59; discussion ons59-60.
495. Groeneveld E, Broeze KA, Lambers MJ, et al. Is aspirin effective in women undergoing in vitro fertilization (IVF)? Results from an individual patient data meta-analysis (IPD MA). Hum Reprod Update 2011;**17**(4):501-9.
496. Groeneveld E, Lambers MJ, Lambalk CB, et al. Preconceptional low-dose aspirin for the prevention of hypertensive pregnancy complications and preterm delivery after IVF: a meta-analysis with individual patient data. Hum Reprod 2013;**28**(6):1480-8.
497. Groves L, Shellenberger MK, Davis CS. Tizanidine treatment of spasticity: a meta-analysis of controlled, double-blind, comparative studies with baclofen and diazepam. Adv Ther 1998;**15**(4):241-51.
498. Gruss HJ, Pediconi C, Jacobs A. Meta-analysis for cardiovascular effects of NRL001 after rectal application in healthy volunteers. Colorectal Dis 2014;**16**(S1):51-58.
499. Gueyffier F, Boissel J-P, Pocock S, et al. Identification of risk factors in hypertensive patients contribution of randomized controlled trials through an individual patient database. Circulation 1999;**100**(18):e88-e94.
500. Gueyffier F, Boutitie F, Boissel JP, et al. Effect of antihypertensive drug treatment on cardiovascular outcomes in women and men. A meta-analysis of individual patient data from randomized, controlled trials. The INDANA Investigators. Ann Intern Med 1997;**126**(10):761-7.
501. Guirao X, Sanchez Garcia M, Bassetti M, et al. Safety and tolerability of tigecycline for the treatment of complicated skin and soft-tissue and intra-abdominal infections: an analysis based on five European observational studies. J Antimicrob Chemother 2013;**68 Suppl 2**:ii37-44.
502. Gulli G, Marquardt L, Rothwell PM, et al. Stroke risk after posterior circulation stroke/transient ischemic attack and its relationship to site of vertebrobasilar stenosis: pooled data analysis from prospective studies. Stroke 2013;**44**(3):598-604.

503. Gupta V, Richards S, Rowe J, et al. Allogeneic, but not autologous, hematopoietic cell transplantation improves survival only among younger adults with acute lymphoblastic leukemia in first remission: an individual patient data meta-analysis. Blood 2013;**121**(2):339-50.
504. Guthmann J-P, Pinoges L, Checchi F, et al. Methodological issues in the assessment of antimalarial drug treatment: Analysis of 13 studies in eight African countries from 2001 to 2004. Antimicrob Agents Chemother 2006;**50**(11):3734-39.
505. Gyongyosi M, Wojakowski W, Lemarchand P, et al. Meta-Analysis of Cell-based CaRdiac stUdiEs (ACCRUE) in patients with acute myocardial infarction based on individual patient data. Circ Res 2015;**116**(8):1346-60.
506. Ha TC, Spreafico F, Graf N, et al. An international strategy to determine the role of high dose therapy in recurrent Wilms' tumour. Eur J Cancer 2013;**49**(1):194-210.
507. Haasova M, Warren FC, Ussher M, et al. The acute effects of physical activity on cigarette cravings: systematic review and meta-analysis with individual participant data. Addiction 2013;**108**(1):26-37.
508. Hacke W, Donnan G, Fieschi C, et al. Association of outcome with early stroke treatment: pooled analysis of ATLANTIS, ECASS, and NINDS rt-PA stroke trials. Lancet 2004;**363**(9411):768-74.
509. Halkes PHA, Gray LJ, Bath PMW, et al. Dipyridamole plus aspirin versus aspirin alone in secondary prevention after TIA or stroke: A meta-analysis by risk. Journal of Neurology, Neurosurgery and Psychiatry 2008;**79**(11):1218-23.
510. Hallahan B, Newell J, Soares JC, et al. Structural magnetic resonance imaging in bipolar disorder: an international collaborative mega-analysis of individual adult patient data. Biol Psychiatry 2011;**69**(4):326-35.
511. Haller DG, O'Connell MJ, Cartwright TH, et al. Impact of age and medical comorbidity on adjuvant treatment outcomes for stage III colon cancer: A pooled analysis of individual patient data from four randomized, controlled trials. Ann Oncol 2015;**26**(4):715-24.
512. Halpin D, Menjoge S, Viel K. Patient-level pooled analysis of the effect of tiotropium on COPD exacerbations and related hospitalisations. Prim Care Respir J 2009;**18**(2):106-13.
513. Hamada C, Tanaka F, Ohta M, et al. Meta-analysis of postoperative adjuvant chemotherapy with tegafur-uracil in non-small-cell lung cancer. J Clin Oncol 2005;**23**(22):4999-5006.
514. Hamajima N, Hirose K, Tajima K, et al. Alcohol, tobacco and breast cancer--collaborative reanalysis of individual data from 53 epidemiological studies, including 58,515 women with breast cancer and 95,067 women without the disease. Br J Cancer 2002;**87**(11):1234-45.
515. Hammad TA, Laughren T, Racoosin J. Suicidality in pediatric patients treated with antidepressant drugs. Arch Gen Psychiatry 2006;**63**(3):332-39.
516. Hansen RA, Song L, Moore CG, et al. Effect of ropinirole on sleep outcomes in patients with restless legs syndrome: meta-analysis of pooled individual patient data from randomized controlled trials. Pharmacotherapy:The Journal of Human Pharmacology and Drug Therapy 2009;**29**(3):255-62.
517. Harada E, Schacht A, Koyama T, et al. Efficacy comparison of duloxetine and SSRIs at doses approved in Japan. Neuropsychiatr Dis Treat 2015;**11**:115-23.
518. Harris R, Whittemore AS, Itnyre J. Characteristics Relating to Ovarian Cancer Risk: Collaborative Analysis of 12 US Case-Control Studies III. Epithelial Tumors of Low Malignant Potential in White Women. Am J Epidemiol 1992;**136**(10):1204-11.
519. Harris R, Wilson-Barnett J, Griffiths P. Effectiveness of nursing-led inpatient care for patients with post-acute health care needs: Secondary data analysis from a programme of randomized controlled trials. J Eval Clin Pract 2007;**13**(2):198-205.
520. Harris ST, Blumentals WA, Miller PD. Ibandronate and the risk of non-vertebral and clinical fractures in women with postmenopausal osteoporosis: Results of a meta-analysis of phase III studies. Curr Med Res Opin 2008;**24**(1):237-45.
521. Hasan B, Greillier L, Pallis A, et al. Progression free survival rate at 9 and 18 weeks predict overall survival in patients with malignant pleural mesothelioma: an individual patient pooled analysis of 10 European Organisation for Research and Treatment of Cancer Lung Cancer Group studies and an independent study validation. Eur J Cancer 2014;**50**(16):2771-82.
522. Hasford J, Pfirrmann M, Hehlmann R, et al. A new prognostic score for survival of patients with chronic myeloid leukemia treated with interferon alfa. Writing Committee for the Collaborative CML Prognostic Factors Project Group. Journal of the National Cancer Institute: JNCI 1998;**90**(11):850-58.
523. Hasford J, Pfirrmann M, Shepherd P, et al. The impact of the combination of baseline risk group and cytogenetic response on the survival of patients with chronic myeloid leukemia treated with interferon-alpha. Haematologica 2005;**90**(3):335-40.
524. Hassan C, Zullo A, De F, V, et al. Systematic review: Endoscopic dilatation in Crohn's disease. Aliment Pharmacol Ther 2007;**26**(11-12):1457-64.

525. Hassell MECJ, Hildick-Smith D, Durand E, et al. Antiplatelet therapy following transcatheter aortic valve implantation. Heart 2015;**101**(14):1118-25.
526. He W, Chen F, Dalm B, et al. Metastatic involvement of the pituitary gland: a systematic review with pooled individual patient data analysis. Pituitary 2014:1-10.
527. He W, Gandhi CD, Quinn J, et al. True aneurysms of the posterior communicating artery: a systematic review and meta-analysis of individual patient data. World Neurosurg 2011;**75**(1):64-72; discussion 49.
528. Healey JS, Hallstrom AP, Kuck K-H, et al. Role of the implantable defibrillator among elderly patients with a history of life-threatening ventricular arrhythmias. Eur Heart J 2007;**28**(14):1746-49.
529. Healey JS, Toff WD, Lamas GA, et al. Cardiovascular outcomes with atrial-based pacing compared with ventricular pacing: Meta-analysis of randomized trials, using individual patient data. Circulation 2006;**114**(1):11-17.
530. Heffner JE, Nietert PJ, Barbieri C. Pleural fluid pH as a predictor of pleurodesis failure: analysis of primary data. Chest 2000;**117**(1):87-95.
531. Heffner JE, Sahn SA, Brown LK. Multilevel likelihood ratios for identifying exudative pleural effusions(*). Chest 2002;**121**(6):1916-20.
532. Heikkilä K, Fransson EI, Nyberg ST, et al. Job strain and health-related lifestyle: Findings from an individual-participant meta-analysis of 118,000 working adults. Am J Public Health 2013;**103**(11):2090-97.
533. Heikkilä K, Madsen IEH, Nyberg ST, et al. Job strain and the risk of inflammatory bowel diseases: Individual-participant meta-analysis of 95 000 men and women. PLoS ONE [Electronic Resource] 2014;**9**(2).
534. Heikkilä K, Madsen IEH, Nyberg ST, et al. Job strain and the risk of severe asthma exacerbations: A meta-analysis of individual-participant data from 100 000 European men and women. Allergy: European Journal of Allergy and Clinical Immunology 2014;**69**(6):775-83.
535. Heikkila K, Nyberg ST, Fransson EI, et al. Job strain and alcohol intake: a collaborative meta-analysis of individual-participant data from 140,000 men and women. PLoS ONE [Electronic Resource] 2012;**7**(7):e40101.
536. Heikkila K, Nyberg ST, Fransson EI, et al. Job strain and tobacco smoking: an individual-participant data meta-analysis of 166,130 adults in 15 European studies. PLoS ONE [Electronic Resource] 2012;**7**(7):e35463.
537. Heikkila K, Nyberg ST, Theorell T, et al. Work stress and risk of cancer: meta-analysis of 5700 incident cancer events in 116,000 European men and women. BMJ 2013;**346**:f165.
538. Heinemann V, Boeck S, Hinke A, et al. Meta-analysis of randomized trials: evaluation of benefit from gemcitabine-based combination chemotherapy applied in advanced pancreatic cancer. BMC Cancer 2008;**8**:82.
539. Heinemann V, Labianca R, Hinke A, et al. Increased survival using platinum analog combined with gemcitabine as compared to single-agent gemcitabine in advanced pancreatic cancer: Pooled analysis of two randomized trials, the GERCOR/GISCAD intergroup study and a German multicenter study. Ann Oncol 2007;**18**(10):1652-59.
540. Helicobacter and Cancer Collaborative G. Gastric cancer and Helicobacter pylori: a combined analysis of 12 case control studies nested within prospective cohorts. [Review] [36 refs]. Gut 2001;**49**(3):347-53.
541. Heller S, Bode B, Kozlovski P, et al. Meta-analysis of insulin aspart versus regular human insulin used in a basal-bolus regimen for the treatment of diabetes mellitus. J Diabetes 2013;**5**(4):482-91.
542. Heneghan C, Ward A, Perera R, et al. Self-monitoring of oral anticoagulation: systematic review and meta-analysis of individual patient data. Lancet 2012;**379**(9813):322-34.
543. Hicks LK, Cheung MC, Ding K, et al. Venous thromboembolism and nonsmall cell lung cancer: a pooled analysis of National Cancer Institute of Canada Clinical Trials Group trials. Cancer 2009;**115**(23):5516-25.
544. Higgins TS, Thorp B, Rawlings BA, et al. Outcome results of endoscopic vs craniofacial resection of sinonasal malignancies: a systematic review and pooled-data analysis. Int Forum Allergy Rhinol 2011;**1**(4):255-61.
545. Hill CJ, Maxwell AP, Cardwell CR, et al. Glycated hemoglobin and risk of death in diabetic patients treated with hemodialysis: a meta-analysis. Am J Kidney Dis 2014;**63**(1):84-94.
546. Hills RK, Castaigne S, Appelbaum FR, et al. Addition of gemtuzumab ozogamicin to induction chemotherapy in adult patients with acute myeloid leukaemia: a meta-analysis of individual patient data from randomised controlled trials. Lancet Oncol 2014;**15**(9):986-96.
547. HIV SURROGATE MARKER COLLABORATIVE GROUP. Human immunodeficiency virus type 1 RNA level and CD4 count as prognostic markers and surrogate end points: A meta-analysis. AIDS Res Hum Retroviruses 2000;**16**(12):1123-33.
548. Hlatky MA, Boothroyd DB, Bravata DM, et al. Coronary artery bypass surgery compared with percutaneous coronary interventions for multivessel disease: a collaborative analysis of individual patient data from ten randomised trials. Lancet 2009;**373**(9670):1190-7.

549. Hollaar G, Gooszen H, Post S, et al. Perioperative Blood Transfusion Does Not Prevent Recurrence in Crohn's Disease: A Pooled Analysis. J Clin Gastroenterol 1995;**21**(2):134-38.
550. Hollevoet K, Reitsma JB, Creaney J, et al. Serum mesothelin for diagnosing malignant pleural mesothelioma: an individual patient data meta-analysis. J Clin Oncol 2012;**30**(13):1541-9.
551. Holloway KL, Baron MS, Brown R, et al. Deep brain stimulation for dystonia: a meta-analysis. Neuromodulation 2006;**9**(4):253-61.
552. Holmes JM, Lazar EL, Melia BM, et al. Effect of age on response to amblyopia treatment in children. Arch Ophthalmol 2011;**129**(11):1451-7.
553. Holmes MV, Dale CE, Zuccolo L, et al. Association between alcohol and cardiovascular disease: Mendelian randomisation analysis based on individual participant data. BMJ 2014;**349**:g4164.
554. Holmes MV, Frikke-Schmidt R, Melis D, et al. A systematic review and meta-analysis of 130,000 individuals shows smoking does not modify the association of APOE genotype on risk of coronary heart disease. Atherosclerosis 2014;**237**(1):5-12.
555. Holty JE, Guilleminault C. Maxillomandibular advancement for the treatment of obstructive sleep apnea: a systematic review and meta-analysis. Sleep Med Rev 2010;**14**(5):287-97.
556. Holty J-EC, Gould MK, Meinke L, et al. Tuberculosis in liver transplant recipients: a systematic review and meta-analysis of individual patient data. Liver Transpl 2009;**15**(8):894-906.
557. Holzer M, Bernard SA, Hachimi-Idrissi S, et al. Hypothermia for neuroprotection after cardiac arrest: systematic review and individual patient data meta-analysis. [Review] [53 refs]. Crit Care Med 2005;**33**(2):414-8.
558. Home PD, Fritsche A, Schinzel S, et al. Meta-analysis of individual patient data to assess the risk of hypoglycaemia in people with type 2 diabetes using NPH insulin or insulin glargine. Diabetes, Obesity and Metabolism 2010;**12**(9):772-9.
559. Homocysteine Lowering Trialists' Collaboration. Lowering blood homocysteine with folic acid based supplements: meta-analysis of randomised trials. BMJ 1998;**316**(7135):894-98.
560. Homocysteine Studies C. Homocysteine and risk of ischemic heart disease and stroke: a meta-analysis.[see comment]. JAMA 2002;**288**(16):2015-22.
561. Horn J, Naylor AR, Laman DM, et al. Identification of patients at risk for ischaemic cerebral complications after carotid endarterectomy with TCD monitoring. Eur J Vasc Endovasc Surg 2005;**30**(3):270-74.
562. Houben RMGJ, Crampin AC, Ndhlovu R, et al. Human immunodeficiency virus associated tuberculosis more often due to recent infection than reactivation of latent infection. Int J Tuberc Lung Dis 2011;**15**(1):24-31.
563. Houssami N, Turner R, Macaskill P, et al. An individual person data meta-analysis of preoperative magnetic resonance imaging and breast cancer recurrence. J Clin Oncol 2014;**32**(5):392-401.
564. Howell A, Ribeiro G, Swindell R. Effects of radiotherapy and surgery in early breast cancer. An overview of the randomized trials. Early Breast Cancer Trialists' Collaborative Group. N Engl J Med 1995.
565. Hoyo C, Cook MB, Kamangar F, et al. Body mass index in relation to oesophageal and oesophagogastric junction adenocarcinomas: a pooled analysis from the International BEACON Consortium. Int J Epidemiol 2012;**41**(6):1706-18.
566. Huang J, van Gelder JM. The probability of sudden death from rupture of intracranial aneurysms: a meta-analysis. Neurosurgery 2002;**51**(5):1101-5; discussion 05-7.
567. Hubbard J, Thomas DM, Yothers G, et al. Benefits and adverse events in younger versus older patients receiving adjuvant chemotherapy for colon cancer: findings from the Adjuvant Colon Cancer Endpoints data set. J Clin Oncol 2012;**30**(19):2334-9.
568. Hughes MD, Daniels MJ, Fischl MA, et al. CD4 cell count as a surrogate endpoint in HIV clinical trials: a meta-analysis of studies of the AIDS Clinical Trials Group. AIDS 1998;**12**(14):1823-32.
569. Huh D, Mun EY, Larimer ME, et al. Brief motivational interventions for college student drinking may not be as powerful as we think: An individual participant-level data meta-analysis. Alcoholism: Clinical and Experimental Research 2015;**39**(5):919-31.
570. Hukkelhoven CW, Steyerberg EW, Rampen AJ, et al. Patient age and outcome following severe traumatic brain injury: an analysis of 5600 patients. J Neurosurg 2003;**99**(4):666-73.
571. Hurwitz HI, Saltz LB, Van Cutsem E, et al. Venous thromboembolic events with chemotherapy plus bevacizumab: a pooled analysis of patients in randomized phase II and III studies. J Clin Oncol 2011;**29**(13):1757-64.
572. Hurwitz HI, Tebbutt NC, Kabbinavar F, et al. Efficacy and safety of bevacizumab in metastatic colorectal cancer: Pooled analysis from seven randomized controlled trials. Oncologist 2013;**18**(9):1004-12.
573. Husain Q, Kanumuri VV, Svider PF, et al. Sinonasal adenoid cystic carcinoma: systematic review of survival and treatment strategies. Otolaryngology - Head and Neck Surgery 2013;**148**(1):29-39.

574. Huth C, Heid IM, Vollmert C, et al. IL6 gene promoter polymorphisms and type 2 diabetes: joint analysis of individual participants' data from 21 studies. Diabetes 2006;**55**(10):2915-21.
575. Huth C, Illig T, Herder C, et al. Joint analysis of individual participants' data from 17 studies on the association of the IL6 variant -174G>C with circulating glucose levels, interleukin-6 levels, and body mass index. Ann Med 2009;**41**(2):128-38.
576. Huxley R. The impact of cardiovascular risk factors on the age-related excess risk of coronary heart disease. Int J Epidemiol 2006;**35**(4):1025-33.
577. Huxley R, Collaboration APCS. The impact of modifiable risk factors on mortality from prostate cancer in populations of the Asia-Pacific region. Asian Pac J Cancer Prev 2007;**8**(2):199-205.
578. Huxley R, Okayama A, Ueshima H, et al. Does sex matter in the associations between classic risk factors and fatal coronary heart disease in populations from the Asia-Pacific region? J Womens Health 2005;**14**(9):820-27.
579. Huxley RR, Barzi F, Lam TH, et al. Isolated low levels of high-density lipoprotein cholesterol are associated with an increased risk of coronary heart disease: an individual participant data meta-analysis of 23 studies in the Asia-Pacific region. Circulation 2011;**124**(19):2056-64.
580. Huxley RR, Barzi F, Woo J, et al. A comparison of risk factors for mortality from heart failure in Asian and non-Asian populations: an overview of individual participant data from 32 prospective cohorts from the Asia-Pacific Region. BMC Cardiovasc Disord 2014;**14**:61.
581. Hyun KK, Huxley RR, Arima H, et al. A comparative analysis of risk factors and stroke risk for Asian and non-Asian men: The Asia Pacific Cohort Studies Collaboration. Int J Stroke 2013;**8**(8):606-11.
582. Iijima R, Byrne RA, Dibra A, et al. Drug-eluting stents versus bare-metal stents in diabetic patients with ST-segment elevation acute myocardial infarction: a pooled analysis of individual patient data from seven randomized trials. Revista Espanola de Cardiologia 2009;**62**(4):354-64.
583. Inglis SC, Bebchuk J, Al-Suhaim SA, et al. Peripheral artery disease and outcomes after myocardial infarction: an individual-patient meta-analysis of 28,771 patients in CAPRICORN, EPEHESUS, OPTIMAAL and VALIANT. Int J Cardiol 2013;**168**(2):1094-101.
584. Inker LA, Levey AS, Pandya K, et al. Early Change in Proteinuria as a Surrogate End Point for Kidney Disease Progression: An Individual Patient Meta-analysis. Am J Kidney Dis 2014.
585. International Collaboration of Epidemiological Studies of Cervical Cancer. Cervical carcinoma and reproductive factors: collaborative reanalysis of individual data on 16,563 women with cervical carcinoma and 33,542 women without cervical carcinoma from 25 epidemiological studies. Int J Cancer 2006;**119**(5):1108-24.
586. International Collaboration of Epidemiological Studies of Cervical Cancer. Comparison of risk factors for invasive squamous cell carcinoma and adenocarcinoma of the cervix: collaborative reanalysis of individual data on 8,097 women with squamous cell carcinoma and 1,374 women with adenocarcinoma from 12 epidemiological studies. Int J Cancer 2007;**120**(4):885-91.
587. International Perinatal HIV Group. The mode of delivery and the risk of vertical transmission of human immunodeficiency virus type 1--a meta-analysis of 15 prospective cohort studies. . N Engl J Med 1999;**340**(13):977-87.
588. International Perinatal HIVG. Duration of ruptured membranes and vertical transmission of HIV-1: a meta-analysis from 15 prospective cohort studies. AIDS 2001;**15**(3):357-68.
589. Ioannidis JP, Collier AC, Cooper DA, et al. Clinical efficacy of high-dose acyclovir in patients with human immunodeficiency virus infection: a meta-analysis of randomized individual patient data. J Infect Dis 1998;**178**(2):349-59.
590. Ioannidis JP, Rosenberg PS, Goedert JJ, et al. Effects of CCR5-Delta32, CCR2-64I, and SDF-1 3'A alleles on HIV-1 disease progression: An international meta-analysis of individual-patient data. Ann Intern Med 2001;**135**(9):782-95.
591. Ioannidis JP, Tektonidou MG, Vlachoyiannopoulos PG, et al. HLA associations of anti-beta2 glycoprotein I response in a Greek cohort with antiphospholipid syndrome and meta-analysis of four ethnic groups. Hum Immunol 1999;**60**(12):1274-80.
592. Ioannidis JP, Vlachoyiannopoulos PG, Haidich AB, et al. Mortality in systemic sclerosis: an international meta-analysis of individual patient data. Am J Med 2005;**118**(1):2-10.
593. Iorio A, Marcucci M, Cheng J, et al. Patient data meta-analysis of Post-Authorization Safety Surveillance (PASS) studies of haemophilia A patients treated with rAHF-PFM. Haemophilia 2014;**20**(6):777-83.
594. Jackson CA, Hutchison A, Dennis MS, et al. Differing risk factor profiles of ischemic stroke subtypes: evidence for a distinct lacunar arteriopathy? Stroke 2010;**41**(4):624-9.
595. Jafar TH, Schmid CH, Landa M, et al. Angiotensin-converting enzyme inhibitors and progression of nondiabetic renal disease. A meta-analysis of patient-level data. Ann Intern Med 2001;**135**(2):73-87.

596. Jafar TH, Schmid CH, Stark PC, et al. The rate of progression of renal disease may not be slower in women compared with men: a patient-level meta-analysis. Nephrol Dial Transplant 2003;**18**(10):2047-53.
597. Jafar TH, Stark PC, Schmid CH, et al. Progression of chronic kidney disease: the role of blood pressure control, proteinuria, and angiotensin-converting enzyme inhibition: a patient-level meta-analysis. Ann Intern Med 2003;**139**(4):244-52.
598. Jaja BNR, Lingsma H, Schweizer TA, et al. Prognostic value of premorbid hypertension and neurological status in aneurysmal subarachnoid hemorrhage: pooled analyses of individual patient data in the SAHIT repository. J Neurosurg 2015;**122**(3):644-52.
599. Janni W, Vogl FD, Wiedswang G, et al. Persistence of disseminated tumor cells in the bone marrow of breast cancer patients predicts increased risk for relapse--a European pooled analysis. Clin Cancer Res 2011;**17**(9):2967-76.
600. Jansen WJ, Ossenkoppele R, Knol DL, et al. Prevalence of cerebral amyloid pathology in persons without dementia: a meta-analysis. JAMA 2015;**313**(19):1924-38.
601. Jauch A, Fsadni M, Gamba G. Meta-analysis of six clinical phase III studies comparing lomefloxacin 0.3% eye drops twice daily to five standard antibiotics in patients with acute bacterial conjunctivitis. Graefe's archive for clinical and experimental ophthalmology 1999;**237**(9):705-13.
602. Jefferis J, Perera R, Everitt H, et al. Acute infective conjunctivitis in primary care: who needs antibiotics? An individual patient data meta-analysis. Br J Gen Pract 2011;**61**(590):e542-8.
603. Jefferson T, Jones MA, Doshi P, et al. Neuraminidase inhibitors for preventing and treating influenza in healthy adults and children. Cochrane Database Syst Rev 2012;**1**:CD008965.
604. Jeger RV, Urban P, Harkness SM, et al. Early revascularization is beneficial across all ages and a wide spectrum of cardiogenic shock severity: A pooled analysis of trials. Acute Card Care 2011;**13**(1):14-20.
605. Jiang W, Yao J, He Y, et al. The timing of surgical treatment of knee dislocations: a systematic review. Knee Surgery, Sports Traumatology, Arthroscopy 2014.
606. Jokela M, Hintsanen M, Hakulinen C, et al. Association of personality with the development and persistence of obesity: a meta-analysis based on individual-participant data. Obes Rev 2013;**14**(4):315-23.
607. Jones PW, Mahler DA, Gale R, et al. Profiling the effects of indacaterol on dyspnoea and health status in patients with COPD. Respir Med 2011;**105**(6):892-9.
608. Jones RW, Schwam E, Wilkinson D, et al. Rates of cognitive change in Alzheimer disease: observations across a decade of placebo-controlled clinical trials with donepezil. Alzheimer Dis Assoc Disord 2009;**23**(4):357-64.
609. Jorgensen AL, Alfirevic Z, Tudur SC, et al. Cervical stitch (cerclage) for preventing pregnancy loss: individual patient data meta-analysis.[see comment]. [Review] [29 refs]. BJOG 2007;**114**(12):1460-76.
610. Joye I, Deroose CM, Vandecaveye V, et al. The role of diffusion-weighted MRI and (18)F-FDG PET/CT in the prediction of pathologic complete response after radiochemotherapy for rectal cancer: a systematic review. Radiother Oncol 2014;**113**(2):158-65.
611. Jumaily JS, Noordzij JP, Dukas AG, et al. Prediction of hypocalcemia after using 1- to 6-hour postoperative parathyroid hormone and calcium levels: an analysis of pooled individual patient data from 3 observational studies. Head Neck 2010;**32**(4):427-34.
612. Kahi CJ, Jensen DM, Sung JJY, et al. Endoscopic therapy versus medical therapy for bleeding peptic ulcer with adherent clot: A meta-analysis. (vol 129, pg 855, 2005). Gastroenterology 2006;**131**(3):980-81.
613. Kaiser R, Barton JL, Chang M, et al. Factor V Leiden and thrombosis in patients with systemic lupus erythematosus: a meta-analysis. Genes Immun 2009;**10**(5):495-502.
614. Kanda J, Ichinohe T, Matsuo K, et al. Impact of ABO mismatching on the outcomes of allogeneic related and unrelated blood and marrow stem cell transplantations for hematologic malignancies: IPD-based meta-analysis of cohort studies. Transfusion (Paris) 2009;**49**(4):624-35.
615. Kanis JA, Barton IP, Johnell O. Risedronate decreases fracture risk in patients selected solely on the basis of prior vertebral fracture. Osteoporos Int 2005;**16**(5):475-82.
616. Kaptoge S, Di Angelantonio E, Lowe G, et al. C-reactive protein concentration and risk of coronary heart disease, stroke, and mortality: an individual participant meta-analysis. Lancet 2010;**375**(9709):132-40.
617. Karlson BW, Barter PJ, Palmer MK, et al. Comparison of the effects of different statins and doses on lipid levels in patients with diabetes: results from VOYAGER. Nutrition Metabolism and Cardiovascular Diseases 2012;**22**(9):697-703.
618. Karyotaki E, Kleiboer A, Smit F, et al. Predictors of treatment dropout in self-guided web-based interventions for depression: an 'individual patient data' meta-analysis. Psychol Med 2015.
619. Kasper S, Corruble E, Hale A, et al. Antidepressant efficacy of agomelatine versus SSRI/SNRI: results from a pooled analysis of head-to-head studies without a placebo control. Int Clin Psychopharmacol 2013;**28**(1):12-9.

620. Kasper S, Dienel A. Cluster analysis of symptoms during antidepressant treatment with Hypericum extract in mildly to moderately depressed out-patients. A meta-analysis of data from three randomized, placebo-controlled trials. Psychopharmacology (Berl) 2002;**164**(3):301-8.
621. Kassai B, Chiron C, Augier S, et al. Severe myoclonic epilepsy in infancy: A systematic review and a meta-analysis of individual patient data. Epilepsia 2008;**49**(2):343-48.
622. Kastrati A, Dibra A, Spaulding C, et al. Meta-analysis of randomized trials on drug-eluting stents vs. bare-metal stents in patients with acute myocardial infarction. Eur Heart J 2007;**28**(22):2706-13.
623. Kastrati A, Mehilli J, Pache J, et al. Analysis of 14 trials comparing sirolimus-eluting stents with bare-metal stents. N Engl J Med 2007;**356**(10):1030-9.
624. Kasturi KS, Chennareddygari S, Mummadi RR. Effect of bisphosphonates on bone mineral density in liver transplant patients: a meta-analysis and systematic review of randomized controlled trials. Transpl Int 2010;**23**(2):200-7.
625. Katanoda K, Marugame T, Saika K, et al. Population attributable fraction of mortality associated with tobacco smoking in Japan: a pooled analysis of three large-scale cohort studies. J Epidemiol 2008;**18**(6):251-64.
626. Kazlauskaite R, Evans AT, Villabona CV, et al. Corticotropin tests for hypothalamic-pituitary- adrenal insufficiency: a metaanalysis. J Clin Endocrinol Metab 2008;**93**(11):4245-53.
627. Kelder JC, Cowie MR, McDonagh TA, et al. Quantifying the added value of BNP in suspected heart failure in general practice: an individual patient data meta-analysis. Heart 2011;**97**(12):959-63.
628. Kelley GA, Kelley KS. Efficacy of resistance exercise on lumbar spine and femoral neck bone mineral density in premenopausal women: a meta-analysis of individual patient data. J Womens Health 2004;**13**(3):293-300.
629. Kelley GA, Kelley KS. Exercise and bone mineral density at the femoral neck in postmenopausal women: A meta-analysis of controlled clinical trials with individual patient data. Am J Obstet Gynecol 2006;**194**(3):760-67.
630. Kelley GA, Kelley KS, Tran ZV. Exercise and lumbar spine bone mineral density in postmenopausal women: a meta-analysis of individual patient data. Journals of Gerontology Series A-Biological Sciences and Medical Sciences 2002;**57**(9):M599-604.
631. Kendrick D, Smith S, Sutton A, et al. Effect of education and safety equipment on poisoning-prevention practices and poisoning: Systematic review, meta-analysis and meta-regression. Arch Dis Child 2008;**93**(7):599-608.
632. Kendrick D, Smith S, Sutton AJ, et al. The effect of education and home safety equipment on childhood thermal injury prevention: meta-analysis and meta-regression. Inj Prev 2009;**15**(3):197-204.
633. Kendrick D, Watson MC, Mulvaney CA, et al. Preventing Childhood Falls at Home. Meta-Analysis and Meta-Regression. Am J Prev Med 2008;**35**(4):370-79.
634. Kendrick D, Young B, Mason-Jones AJ, et al. Home safety education and provision of safety equipment for injury prevention. Cochrane Database Syst Rev 2012;**9**:CD005014.
635. Kengne AP, Nakamura K, Barzi F, et al. Smoking, diabetes and cardiovascular diseases in men in the Asia Pacific region. J Diabetes 2009;**1**(3):173-81.
636. Kengne AP, Patel A, Barzi F, et al. Systolic blood pressure, diabetes and the risk of cardiovascular diseases in the Asia-Pacific region. Journal of Hypertension 2007;**25**(6):1205-13.
637. Kennedy SH, Andersen HF, Thase ME. Escitalopram in the treatment of major depressive disorder: a meta-analysis. Curr Med Res Opin 2009;**25**(1):161-75.
638. Key T, Appleby P, Barnes I, et al. Endogenous sex hormones and breast cancer in postmenopausal women: reanalysis of nine prospective studies. J Natl Cancer Inst 2002;**94**(8):606-16.
639. Key TJ, Appleby PN, Reeves GK, et al. Insulin-like growth factor 1 (IGF1), IGF binding protein 3 (IGFBP3), and breast cancer risk: pooled individual data analysis of 17 prospective studies. Lancet Oncol 2010;**11**(6):530-42.
640. Key TJ, Fraser GE, Thorogood M, et al. Mortality in vegetarians and non-vegetarians: a collaborative analysis of 8300 deaths among 76,000 men and women in five prospective studies. Public Health Nutr 1998;**1**(01):33-41.
641. Khalil AA, Bentzen SM, Bernier J, et al. Compliance to the prescribed dose and overall treatment time in five randomized clinical trials of altered fractionation in radiotherapy for head-and-neck carcinomas. International Journal of Radiation Oncology, Biology, Physics 2003;**55**(3):568-75.
642. Khedmat H, Amini M, Ghamar-Chehreh ME. Colorectal involvement by post-transplant lymphoproliferative disorders: a review of 81 cases. Saudi J Kidney Dis Transpl 2014;**25**(3):597-604.
643. Kheifets L, Ahlbom A, Crespi CM, et al. Pooled analysis of recent studies on magnetic fields and childhood leukaemia. Br J Cancer 2010;**103**(7):1128-35.

644. Kheifets L, Ahlbom A, Crespi CM, et al. A pooled analysis of extremely low-frequency magnetic fields and childhood brain tumors. Am J Epidemiol 2010;**172**(7):752-61.
645. KIDS-ART-LINC Collaboration. Low risk of death, but substantial program attrition, in pediatric HIV treatment cohorts in Sub-Saharan Africa. J Acquir Immune Defic Syndr 2008;**49**(5):523-31.
646. Kim H, Al-Shahi Salman R, McCulloch CE, et al. Untreated brain arteriovenous malformation: patient-level meta-analysis of hemorrhage predictors. Neurology 2014;**83**(7):590-7.
647. Kim PW, Wu YT, Cooper C, et al. Meta-analysis of a possible signal of increased mortality associated with cefepime use. Clin Infect Dis 2010;**51**(4):381-9.
648. Kirtane AJ, Ellis SG, Dawkins KD, et al. Paclitaxel-eluting coronary stents in patients with diabetes mellitus: pooled analysis from 5 randomized trials. J Am Coll Cardiol 2008;**51**(7):708-15.
649. Kishi T, Hirota T, Iwata N. Add-on fluvoxamine treatment for schizophrenia: An updated meta-analysis of randomized controlled trials. Eur Arch Psychiatry Clin Neurosci 2013;**263**(8):633-41.
650. Kishi T, Matsuda Y, Nakamura H, et al. Blonanserin for schizophrenia: systematic review and meta-analysis of double-blind, randomized, controlled trials. J Psychiatr Res 2013;**47**(2):149-54.
651. Kishi T, Mukai T, Matsuda Y, et al. Selective serotonin 3 receptor antagonist treatment for schizophrenia: Meta-analysis and systematic review. Neuromolecular Med 2014;**16**(1):61-69.
652. Kishi T, Mukai T, Matsuda Y, et al. Efficacy and safety of noradrenalin reuptake inhibitor augmentation therapy for schizophrenia: a meta-analysis of double-blind randomized placebo-controlled trials. J Psychiatr Res 2013;**47**(11):1557-63.
653. Kivimaki M, Nyberg ST, Batty GD, et al. Job strain as a risk factor for coronary heart disease: a collaborative meta-analysis of individual participant data. Lancet 2012;**380**(9852):1491-7.
654. Kivimäki M, Virtanen M, Kawachi I, et al. Long working hours, socioeconomic status, and the risk of incident type 2 diabetes: A meta-analysis of published and unpublished data from 222120 individuals. Lancet Diabetes Endocrinol 2015;**3**(1):27-34.
655. Kleinrouweler CE, Bossuyt PMM, Thilaganathan B, et al. Value of adding second-trimester uterine artery Doppler to patient characteristics in identification of nulliparous women at increased risk for pre-eclampsia: an individual patient data meta-analysis. Ultrasound Obstet Gynecol 2013;**42**(3):257-67.
656. Klerk M, Verhoef P, Clarke R, et al. MTHFR 677C-->T polymorphism and risk of coronary heart disease: a meta-analysis.[see comment]. JAMA 2002;**288**(16):2023-31.
657. Klijn JG, Blamey RW, Boccardo F, et al. Combined tamoxifen and luteinizing hormone-releasing hormone (LHRH) agonist versus LHRH agonist alone in premenopausal advanced breast cancer: a meta-analysis of four randomized trials.[see comment]. J Clin Oncol 2001;**19**(2):343-53.
658. Klotz L, Miller K, Crawford ED, et al. Disease Control Outcomes from Analysis of Pooled Individual Patient Data from Five Comparative Randomised Clinical Trials of Degarelix Versus Luteinising Hormone-releasing Hormone Agonists. Eur Urol 2014.
659. Klug SJ, Ressing M, Koenig J, et al. TP53 codon 72 polymorphism and cervical cancer: a pooled analysis of individual data from 49 studies. Lancet Oncol 2009;**10**(8):772-84.
660. Knoll GA, Kokolo MB, Mallick R, et al. Effect of sirolimus on malignancy and survival after kidney transplantation: systematic review and meta-analysis of individual patient data. BMJ 2014;**349**:g6679.
661. Koch A, Ziegler S, Breitschwerdt H, et al. Low molecular weight heparin and unfractionated heparin in thrombosis prophylaxis: meta-analysis based on original patient data. Thromb Res 2001;**102**(4):295-309.
662. Koeter MWJ, van den Brink W, Lehert P. Effect of early and late compliance on the effectiveness of acamprosate in the treatment of alcohol dependence. J Subst Abuse Treat 2010;**39**(3):218-26.
663. Kolli KK, Arif I, Peelukhana SV, et al. Diagnostic performance of pressure drop coefficient in relation to fractional flow reserve and coronary flow reserve. J Invasive Cardiol 2014;**26**(5):188-95.
664. Koopman L, Hoes AW, Glasziou PP, et al. Antibiotic therapy to prevent the development of asymptomatic middle ear effusion in children with acute otitis media: A meta-analysis of individual patient data. Arch Otolaryngol Head Neck Surg 2008;**134**(2):128-32.
665. Kotecha D, Holmes J, Krum H, et al. Efficacy of beta blockers in patients with heart failure plus atrial fibrillation: an individual-patient data meta-analysis. Lancet 2014;**384**(9961):2235-43.
666. Kotecha RS, Jacoby P, Cole CH, et al. Morbidity in survivors of child and adolescent meningioma. Cancer 2013;**119**(24):4350-7.
667. Kotecha RS, Pascoe EM, Rushing EJ, et al. Meningiomas in children and adolescents: a meta-analysis of individual patient data. Lancet Oncol 2011;**12**(13):1229-39.
668. Kovalchik SA. Mother's CD4+ count moderates the risk associated with higher parity for late postnatal HIV-free survival of breastfed children: an individual patient data meta-analysis of randomized controlled trials. AIDS Behav 2012;**16**(1):79-85.

669. Krauter J, Wagner K, Schafer I, et al. Prognostic factors in adult patients up to 60 years old with acute myeloid leukemia and translocations of chromosome band 11q23: individual patient data-based meta-analysis of the German Acute Myeloid Leukemia Intergroup. J Clin Oncol 2009;**27**(18):3000-6.
670. Kricker A, Armstrong BK, Hughes AM, et al. Personal sun exposure and risk of non Hodgkin lymphoma: a pooled analysis from the Interlymph Consortium. Int J Cancer 2008;**122**(1):144-54.
671. Krogsgaard K, Bindslev N, Christensen E, et al. The treatment effect of alpha interferon in chronic hepatitis B is independent of pre-treatment variables. Results based on individual patient data from 10 clinical controlled trials. European Concerted Action on Viral Hepatitis (Eurohep). J Hepatol 1994;**21**(4):646-55.
672. Krogsgaard K, Christensen E, Bindslev N, et al. Relation between treatment efficacy and cumulative dose of alpha interferon in chronic hepatitis B. European Concerted Action on Viral Hepatitis (Eurohep). J Hepatol 1996;**25**(6):795-802.
673. Kubo A, Cook MB, Shaheen NJ, et al. Sex-specific associations between body mass index, waist circumference and the risk of Barrett's oesophagus: a pooled analysis from the international BEACON consortium. Gut 2013;**62**(12):1684-91.
674. Kufner S, de Waha A, Tomai F, et al. A meta-analysis of specifically designed randomized trials of sirolimus-eluting versus paclitaxel-eluting stents in diabetic patients with coronary artery disease. Am Heart J 2011;**162**(4):740-7.
675. Kumagai K, Rouvelas I, Tsai JA, et al. Survival benefit and additional value of preoperative chemoradiotherapy in resectable gastric and gastro-oesophageal junction cancer: a direct and adjusted indirect comparison meta-analysis. Eur J Surg Oncol 2015;**41**(3):282-94.
676. La Vecchia C, Ron E, Franceschi S, et al. A pooled analysis of case-control studies of thyroid cancer¶ III. Oral contraceptives, menopausal replacement therapy and other female hormones. Cancer Causes Control 1999;**10**(2):157-66.
677. Lam TH, Barzi F, Patel A, et al. Smoking, quitting, and the risk of cardiovascular disease among women and men in the Asia-Pacific region. Int J Epidemiol 2005;**34**(5):1036-45.
678. Lammers WJ, van Buuren HR, Hirschfield GM, et al. Levels of alkaline phosphatase and bilirubin are surrogate end points of outcomes of patients with primary biliary cirrhosis: an international follow-up study. Gastroenterology 2014;**147**(6):1338-49.e5; quiz e15.
679. Lampl C, Voelker M, Diener HC. Efficacy and safety of 1,000 mg effervescent aspirin: Individual patient data meta-analysis of three trials in migraine headache and migraine accompanying symptoms. J Neurol 2007;**254**(6):705-12.
680. Lampl C, Voelker M, Steiner TJ. Aspirin is first-line treatment for migraine and episodic tension-type headache regardless of headache intensity. Headache 2012;**52**(1):48-56.
681. Lanas A, McCarthy D, Voelker M, et al. Short-term acetylsalicylic acid (aspirin) use for pain, fever, or colds - gastrointestinal adverse effects: a meta-analysis of randomized clinical trials. Drugs R D 2011;**11**(3):277-88.
682. Landman GWD, van Hateren KJJ, van Dijk PR, et al. Efficacy of device-guided breathing for hypertension in blinded, randomized, active-controlled trials: a meta-analysis of individual patient data. JAMA Intern Med 2014;**174**(11):1815-21.
683. Lane SD, Yechiam E, Busemeyer JR. Application of a computational decision model to examine acute drug effects on human risk taking. Exp Clin Psychopharmacol 2006;**14**(2):254-64.
684. Lange N, Swearer S, Sturner WQ. Human postmortem interval estimation from vitreous potassium: an analysis of original data from six different studies. Forensic Sci Int 1994;**66**(3):159-74.
685. Langhorne P, Taylor G, Murray G, et al. Early supported discharge services for stroke patients: a meta-analysis of individual patients' data.[see comment]. Lancet 2005;**365**(9458):501-6.
686. Lanterna LA, Ruigrok Y, Alexander S, et al. Meta-analysis of APOE genotype and subarachnoid hemorrhage: Clinical outcome and delayed ischemia. Neurology 2007;**69**(8):766-75.
687. Laporte S, Liotier J, Bertoletti L, et al. Individual patient data meta-analysis of enoxaparin vs. unfractionated heparin for venous thromboembolism prevention in medical patients. J Thromb Haemost 2011;**9**(3):464-72.
688. Laporte S, Squifflet P, Baroux N, et al. Prediction of survival benefits from progression-free survival benefits in advanced non-small-cell lung cancer: Evidence from a meta-analysis of 2334 patients from 5 randomised trials. BMJ Open 2013;**3**(3).
689. Lassus J, Gayat E, Mueller C, et al. Incremental value of biomarkers to clinical variables for mortality prediction in acutely decompensated heart failure: the Multinational Observational Cohort on Acute Heart Failure (MOCA) study. Int J Cardiol 2013;**168**(3):2186-94.
690. Latini R, Tognoni G, Maggioni AP, et al. Clinical effects of early angiotensin-converting enzyme inhibitor treatment for acute myocardial infarction are similar in the presence and absence of aspirin: systematic

overview of individual data from 96,712 randomized patients. Angiotensin-converting Enzyme Inhibitor Myocardial Infarction Collaborative Group.[see comment]. J Am Coll Cardiol 2000;**35**(7):1801-7.
691. Lawes CM, Parag V, Bennett DA, et al. Blood glucose and risk of cardiovascular disease in the Asia Pacific region. Diabetes Care 2004;**27**(12):2836-42.
692. Lawes CM, Rodgers A, Bennett DA, et al. Blood pressure and cardiovascular disease in the Asia Pacific region.[see comment]. Journal of Hypertension 2003;**21**(4):707-16.
693. Le Cesne A, Ouali M, Leahy MG, et al. Doxorubicin-based adjuvant chemotherapy in soft tissue sarcoma: pooled analysis of two STBSG-EORTC phase III clinical trials. Ann Oncol 2014;**25**(12):2425-32.
694. Leandro G, Mangia A, Hui J, et al. Relationship between steatosis, inflammation, and fibrosis in chronic hepatitis C: a meta-analysis of individual patient data. Gastroenterology 2006;**130**(6):1636-42.
695. Lee CH, Chung CK, Jahng TA, et al. Which one is a valuable surrogate for predicting survival between Tomita and Tokuhashi scores in patients with spinal metastases? A meta-analysis for diagnostic test accuracy and individual participant data analysis. J Neurooncol 2015;**123**(2):267-75.
696. Lee N, Leo YS, Cao B, et al. Neuraminidase inhibitors, superinfection and corticosteroids affect survival of influenza patients. Eur Respir J 2015;**45**(6):1642-52.
697. Lees KR, Bluhmki E, von Kummer R, et al. Time to treatment with intravenous alteplase and outcome in stroke: an updated pooled analysis of ECASS, ATLANTIS, NINDS, and EPITHET trials. Lancet 2010;**375**(9727):1695-703.
698. Lehert P, Cheron G, Calatayud GA, et al. Racecadotril for childhood gastroenteritis: an individual patient data meta-analysis. Dig Liver Dis 2011;**43**(9):707-13.
699. Lehrke M, Marx N, Patel S, et al. Safety and tolerability of linagliptin in patients with type 2 diabetes: A comprehensive pooled analysis of 22 placebo-controlled studies. Clin Ther 2014;**36**(8):1130-46.
700. Leizorovicz A, Lechat P, Cucherat M, et al. Bisoprolol for the treatment of chronic heart failure: a meta-analysis on individual data of two placebo-controlled studies--CIBIS and CIBIS II. Cardiac Insufficiency Bisoprolol Study. Am Heart J 2002;**143**(2):301-7.
701. Lejoyeux M, Lehert P. Alcohol-use disorders and depression: results from individual patient data meta-analysis of the acamprosate-controlled studies. Alcohol Alcohol 2011;**46**(1):61-7.
702. Lemmens R, Christensen S, Straka M, et al. Patients with single distal MCA perfusion lesions have a high rate of good outcome with or without reperfusion. Int J Stroke 2014;**9**(2):156-9.
703. Leonardi-Bee J, Bath PMW, Bousser M-G, et al. Dipyridamole for preventing recurrent ischemic stroke and other vascular events: A meta-analysis of individual patient data from randomized controlled trials. Stroke 2005;**36**(1):162-68.
704. Leonardi-Bee J, Steiner T, Bath-Hextall F. Naftidrofuryl for acute stroke. Cochrane Database Syst Rev 2007(2):CD005478.
705. Leppik I, Morrell M, Godfroid P, et al. Seizure-free days observed in randomized placebo-controlled add-on trials with levetiracetam in partial epilepsy. Epilepsia 2003;**44**(10):1350-2.
706. Leroy S, Fernandez-Lopez A, Nikfar R, et al. Association of procalcitonin with acute pyelonephritis and renal scars in pediatric UTI. Pediatrics 2013;**131**(5):870-9.
707. Leroy S, Romanello C, Galetto-Lacour A, et al. Procalcitonin is a predictor for high-grade vesicoureteral reflux in children: meta-analysis of individual patient data. J Pediatr 2011;**159**(4):644-51.e4.
708. Leroy V, Halfon P, Bacq Y, et al. Diagnostic accuracy, reproducibility and robustness of fibrosis blood tests in chronic hepatitis C: a meta-analysis with individual data. Clin Biochem 2008;**41**(16-17):1368-76.
709. Leroy V, Newell M-L, Dabis F, et al. International multicentre pooled analysis of late postnatal mother-to-child transmission of HIV-1 infection. Lancet 1998;**352**(9128):597-600.
710. Leucht S, Busch R, Hamann J, et al. Early-onset hypothesis of antipsychotic drug action: A hypothesis tested, confirmed and extended. Biol Psychiatry 2005;**57**(12):1543-49.
711. Leucht S, Busch R, Kissling W, et al. Early prediction of antipsychotic nonresponse among patients with schizophrenia. J Clin Psychiatry 2007;**68**(3):352-60.
712. Levy E, Piedbois P, Buyse M, et al. Toxicity of fluorouracil in patients with advanced colorectal cancer: effect of administration schedule and prognostic factors. Journal of clinical oncology: official journal of the American Society of Clinical Oncology 1998;**16**(11):3537-41.
713. Levy V, Katsahian S, Fermand JP, et al. A meta-analysis on data from 575 patients with multiple myeloma randomly assigned to either high-dose therapy or conventional therapy. Medicine 2005;**8**(4):250-60.
714. Lewington S, Clarke R, Qizilbash N, et al. Age-specific relevance of usual blood pressure to vascular mortality: a meta-analysis of individual data for one million adults in 61 prospective studies.[see comment][erratum appears in Lancet. 2003 Mar 22;361(9362):1060]. Lancet 2002;**360**(9349):1903-13.

715. Lewington S, Whitlock G, Clarke R, et al. Blood cholesterol and vascular mortality by age, sex, and blood pressure: a meta-analysis of individual data from 61 prospective studies with 55,000 vascular deaths. Lancet 2007;**370**(9602):1829-39.
716. Lewis SC, Langman MJ, Laporte JR, et al. Dose-response relationships between individual nonaspirin nonsteroidal anti-inflammatory drugs (NANSAIDs) and serious upper gastrointestinal bleeding: a meta-analysis based on individual patient data. Br J Clin Pharmacol 2002;**54**(3):320-6.
717. LHRH-agonists in Early Breast Cancer Overview group. Use of luteinising-hormone-releasing hormone agonists as adjuvant treatment in premenopausal patients with hormone-receptor-positive breast cancer: a meta-analysis of individual patient data from randomised adjuvant trials. Lancet 2007;**369**(9574):1711-23.
718. Li JZ, Paredes R, Ribaudo HJ, et al. Low-frequency HIV-1 drug resistance mutations and risk of NNRTI-based antiretroviral treatment failure: a systematic review and pooled analysis. JAMA 2011;**305**(13):1327-35.
719. Li W, Gueyffierd F, Liu G, et al. The effect of antihypertensive treatment on cardiovascular events--a meta-analysis of four clinical trials in China. Biomedical and environmental sciences: BES 2001;**14**(4):341-49.
720. Li Wan Po A, Petersen B. How high should total pain-relief score be to obviate the need for analgesic remedication in acute pain? Estimation using signal detection theory and individual-patient meta-analysis. J Clin Pharm Ther 2006;**31**(2):161-5.
721. Lievre M, Gueyffier F, Ekbom T, et al. Efficacy of diuretics and beta-blockers in diabetic hypertensive patients: Results from a meta-analysis. Diabetes Care 2000;**23**(SUPPL. 2):B65-B71.
722. Lim E, Clough R, Goldstraw P, et al. Impact of positive pleural lavage cytology on survival in patients having lung resection for non-small-cell lung cancer: An international individual patient data meta-analysis. J Thorac Cardiovasc Surg 2010;**139**(6):1441-6.
723. Lincoff AM, Wolski K, Nicholls SJ, et al. Pioglitazone and risk of cardiovascular events in patients with type 2 diabetes mellitus: A meta-analysis of randomized trials. J Am Med Assoc 2007;**298**(10):1180-88.
724. Lingsma HF, Roozenbeek B, Li B, et al. Large between-center differences in outcome after moderate and severe traumatic brain injury in the international mission on prognosis and clinical trial design in traumatic brain injury (IMPACT) study. Neurosurgery 2011;**68**(3):601-7; discussion 07-8.
725. Liu PY, Swerdloff RS, Anawalt BD, et al. Determinants of the rate and extent of spermatogenic suppression during hormonal male contraception: An integrated analysis. J Clin Endocrinol Metab 2008;**93**(5):1774-83.
726. Lodrup Carlsen KC, Roll S, Carlsen K-H, et al. Does pet ownership in infancy lead to asthma or allergy at school age? Pooled analysis of individual participant data from 11 European birth cohorts. PLoS ONE [Electronic Resource] 2012;**7**(8):e43214.
727. Loeffler M, Brosteanu O, Hasenclever D, et al. Meta-analysis of chemotherapy versus combined modality treatment trials in Hodgkin's disease. International Database on Hodgkin's Disease Overview Study Group.[see comment]. J Clin Oncol 1998;**16**(3):818-29.
728. Loibl S, Jackisch C, Lederer B, et al. Outcome after neoadjuvant chemotherapy in young breast cancer patients: a pooled analysis of individual patient data from eight prospectively randomized controlled trials. Breast Cancer Res Treat 2015.
729. Loibl S, Skacel T, Nekljudova V, et al. Evaluating the impact of Relative Total Dose Intensity (RTDI) on patients' short and long-term outcome in taxane- and anthracycline-based chemotherapy of metastatic breast cancer- a pooled analysis. BMC Cancer 2011;**11**:131.
730. Longnecker MP, Martin-Moreno JM, Knekt P, et al. Serum alpha-tocopherol concentration in relation to subsequent colorectal cancer: pooled data from five cohorts. J Natl Cancer Inst 1992;**84**(6):430-5.
731. Look MP, van Putten WL, Duffy MJ, et al. Pooled analysis of prognostic impact of urokinase-type plasminogen activator and its inhibitor PAI-1 in 8377 breast cancer patients. J Natl Cancer Inst 2002;**94**(2):116-28.
732. Loprinzi CL, Sloan J, Stearns V, et al. Newer antidepressants and gabapentin for hot flashes: an individual patient pooled analysis. J Clin Oncol 2009;**27**(17):2831-7.
733. Lorenz MW, Polak JF, Kavousi M, et al. Carotid intima-media thickness progression to predict cardiovascular events in the general population (the PROG-IMT collaborative project): a meta-analysis of individual participant data.[Erratum appears in Lancet. 2012 Aug 4;380(9840):474]. Lancet 2012;**379**(9831):2053-62.
734. Losier BJ, Klein RM. A review of the evidence for a disengage deficit following parietal lobe damage. [Review] [57 refs]. Neurosci Biobehav Rev 2001;**25**(1):1-13.
735. Loverix L, Juvonen T, Biancari F. Prosthetic endocarditis after transcatheter aortic valve implantation: Pooled individual patient outcome. Int J Cardiol 2015;**178**:67-68.

736. Low N, Chersich MF, Schmidlin K, et al. Intravaginal practices, bacterial vaginosis, and HIV infection in women: individual participant data meta-analysis. PLoS Medicine / Public Library of Science 2011;**8**(2):e1000416.
737. Lucenteforte E, La Vecchia C, Silverman D, et al. Alcohol consumption and pancreatic cancer: a pooled analysis in the International Pancreatic Cancer Case-Control Consortium (PanC4). Ann Oncol 2012;**23**(2):374-82.
738. Ludwig H, Crawford J, Osterborg A, et al. Pooled analysis of individual patient-level data from all randomized, double-blind, placebo-controlled trials of darbepoetin alfa in the treatment of patients with chemotherapy-induced anemia. J Clin Oncol 2009;**27**(17):2838-47.
739. Maas AIR, Steyerberg EW, Butcher I, et al. Prognostic value of computerized tomography scan characteristics in traumatic brain injury: Results from the IMPACT study. J Neurotrauma 2007;**24**(2):303-14.
740. Maas M, Nelemans PJ, Valentini V, et al. Adjuvant chemotherapy in rectal cancer: defining subgroups who may benefit after neoadjuvant chemoradiation and resection: a pooled analysis of 3,313 patients. Int J Cancer 2015;**137**(1):212-20.
741. Maas M, Nelemans PJ, Valentini V, et al. Long-term outcome in patients with a pathological complete response after chemoradiation for rectal cancer: a pooled analysis of individual patient data. Lancet Oncol 2010;**11**(9):835-44.
742. Mackay HJ, Brady MF, Oza AM, et al. Prognostic relevance of uncommon ovarian histology in women with stage III/IV epithelial ovarian cancer. Int J Gynecol Cancer 2010;**20**(6):945-52.
743. MacPherson H, Maschino AC, Lewith G, et al. Characteristics of acupuncture treatment associated with outcome: an individual patient meta-analysis of 17,922 patients with chronic pain in randomised controlled trials.[Erratum appears in PLoS One. 2013;8(12). doi:10.1371/annotation/23629d97-3b72-474b-9d89-c7198ba43d60 Note: Witt, Claudia [corrected to Witt, Claudia M]]. PLoS ONE [Electronic Resource] 2013;**8**(10):e77438.
744. Mahaffey KW, Van de WF, Shernan SK, et al. Effect of pexelizumab on mortality in patients with acute myocardial infarction or undergoing coronary artery bypass surgery: A systematic overview. Am Heart J 2006;**152**(2):291-96.
745. Mahmoodi BK, Gansevoort RT, Naess IA, et al. Association of mild to moderate chronic kidney disease with venous thromboembolism: pooled analysis of five prospective general population cohorts. Circulation 2012;**126**(16):1964-71.
746. Mahmoodi BK, Yatsuya H, Matsushita K, et al. Association of kidney disease measures with ischemic versus hemorrhagic strokes: Pooled analyses of 4 prospective community-based cohorts. Stroke 2014.
747. Mahner S, Eulenburg C, Staehle A, et al. Prognostic impact of the time interval between surgery and chemotherapy in advanced ovarian cancer: analysis of prospective randomised phase III trials. Eur J Cancer 2013;**49**(1):142-9.
748. Mahr AD, Jover JA, Spiera RF, et al. Adjunctive methotrexate for treatment of giant cell arteritis: An individual patient data meta-analysis. Arthritis Rheum 2007;**56**(8):2789-97.
749. Maino A, Siegerink B, Lotta LA, et al. Plasma ADAMTS-13 levels and the risk of myocardial infarction: An individual patient data meta-analysis. J Thromb Haemost 2015;**13**(8):1396-404.
750. Makanga M, Premji Z, Falade C, et al. Efficacy and safety of the six-dose regimen of artemether-lumefantrine in pediatrics with uncomplicated Plasmodium falciparum malaria: A pooled analysis of individual patient data. Am J Trop Med Hyg 2006;**74**(6):991-98.
751. Malbrain MLNG, Chiumello D, Cesana BM, et al. A systematic review and individual patient data meta-analysis on intra-abdominal hypertension in critically ill patients: The wake-up project. World initiative on Abdominal Hypertension Epidemiology, a Unifying Project (WAKE-Up!). Minerva Anestesiol 2014;**80**(3):293-306.
752. Malmstrom P-U, Sylvester RJ, Crawford DE, et al. An individual patient data meta-analysis of the long-term outcome of randomised studies comparing intravesical mitomycin C versus bacillus Calmette-Guerin for non-muscle-invasive bladder cancer. Eur Urol 2009;**56**(2):247-56.
753. Manassis K, Changgun Le T, Bennett K, et al. Types of parental involvement in CBT with anxious youth: A preliminary meta-analysis. J Consult Clin Psychol 2015;**82**(6):1163-72.
754. Mangano DT. Effects of acadesine on myocardial infarction, stroke, and death following surgery. A meta-analysis of the 5 international randomized trials. The Multicenter Study of Perioperative Ischemia (McSPI) Research Group. JAMA 1997;**277**(4):325-32.
755. Mangia A, Leandro G, Helbling B, et al. Combination therapy with amantadine and interferon in naive patients with chronic hepatitis C: meta-analysis of individual patient data from six clinical trials. J Hepatol 2004;**40**(3):478-83.

756. Man-Son-Hing M, Wells G. Meta-analysis of efficacy of quinine for treatment of nocturnal leg cramps in elderly people.[see comment]. BMJ 1995;**310**(6971):13-7.
757. Man-Son-Hing M, Wells G, Lau A. Quinine for nocturnal leg cramps: a meta-analysis including unpublished data. J Gen Intern Med 1998;**13**(9):600-6.
758. Mant J, Doust J, Roalfe A, et al. Systematic review and individual patient data meta-analysis of diagnosis of heart failure, with modelling of implications of different diagnostic strategies in primary care. Health technology assessment (Winchester, England) 2009;**13**(32):1-207, iii.
759. Marcucci M, Mancuso ME, Santagostino E, et al. Type and intensity of FVIII exposure on inhibitor development in PUPs with haemophilia A: A patient-level meta-analysis. Thromb Haemost 2015;**113**(5):1-10.
760. Margolis DJ, Kantor J, Santanna J, et al. Risk factors for delayed healing of neuropathic diabetic foot ulcers: a pooled analysis. Arch Dermatol 2000;**136**(12):1531-5.
761. Marmarou A, Lu J, Butcher I, et al. Prognostic value of the Glasgow Coma Scale and pupil reactivity in traumatic brain injury assessed pre-hospital and on enrollment: an IMPACT analysis. J Neurotrauma 2007;**24**(2):270-80.
762. Marshall M, Crowther R, Almaraz-Serrano A, et al. Systematic reviews of the effectiveness of day care for people with severe mental disorders: (1) acute day hospital versus admission; (2) vocational rehabilitation; (3) day hospital versus outpatient care. [Review] [66 refs]. Health Technol Assess 2001;**5**(21):1-75.
763. Marshall M, Crowther R, Sledge WH, et al. Day hospital versus admission for acute psychiatric disorders. Cochrane Database Syst Rev 2011(12):CD004026.
764. Marson AG, Williamson PR, Clough H, et al. Carbamazepine versus valproate monotherapy for epilepsy: a meta-analysis. Epilepsia 2002;**43**(5):505-13.
765. Martin J, Donaldson AN, Villarroel R, et al. Efficacy of acupuncture in asthma: systematic review and meta-analysis of published data from 11 randomised controlled trials. Eur Respir J 2002;**20**(4):846-52.
766. Mason BJ, Lehert P. Acamprosate for alcohol dependence: a sex-specific meta-analysis based on individual patient data. Alcoholism: Clinical and Experimental Research 2012;**36**(3):497-508.
767. Mason J, Young P, Freemantle N, et al. Safety and costs of initiating angiotensin converting enzyme inhibitors for heart failure in primary care: analysis of individual patient data from studies of left ventricular dysfunction.[see comment]. BMJ 2000;**321**(7269):1113-6.
768. Mataftsi A, Haidich A-B, Kokkali S, et al. Postoperative glaucoma following infantile cataract surgery: an individual patient data meta-analysis. JAMA Ophthalmol 2014;**132**(9):1059-67.
769. Mathurin P, O'Grady J, Carithers RL, et al. Corticosteroids improve short-term survival in patients with severe alcoholic hepatitis: meta-analysis of individual patient data. Gut 2011;**60**(2):255-60.
770. Matsuda Y, Kishi T, Shibayama H, et al. Yokukansan in the treatment of behavioral and psychological symptoms of dementia: a systematic review and meta-analysis of randomized controlled trials. Human Psychopharmacology 2013;**28**(1):80-6.
771. Matsushita K, Coresh J, Sang Y, et al. Estimated glomerular filtration rate and albuminuria for prediction of cardiovascular outcomes: A collaborative meta-analysis of individual participant data. Lancet Diabetes Endocrinol 2015;**3**(7):514-25.
772. Matsushita K, van der Velde M, Astor BC, et al. Association of estimated glomerular filtration rate and albuminuria with all-cause and cardiovascular mortality in general population cohorts: a collaborative meta-analysis. Lancet 2010;**375**(9731):2073-81.
773. Matsushita Y, Sugihara M, Kaburagi J, et al. Pravastatin use and cancer risk: a meta-analysis of individual patient data from long-term prospective controlled trials in Japan. Pharmacoepidemiol Drug Saf 2010;**19**(2):196-202.
774. Mattila T, Wohlfarth T, Koeter M, et al. Geographic variation in efficacy of atypical antipsychotics for the acute treatment of schizophrenia - An individual patient data meta-analysis. Eur Neuropsychopharmacol 2014.
775. Mauer DK, Nolan J, Plaisance P, et al. Effect of active compression-decompression resuscitation (ACD-CPR) on survival: a combined analysis using individual patient data. Resuscitation 1999;**41**(3):249-56.
776. Mauguen A, Le Pechoux C, Saunders MI, et al. Hyperfractionated or accelerated radiotherapy in lung cancer: an individual patient data meta-analysis. J Clin Oncol 2012;**30**(22):2788-97.
777. Mavros MN, Mayo SC, Hyder O, et al. A systematic review: treatment and prognosis of patients with fibrolamellar hepatocellular carcinoma. J Am Coll Surg 2012;**215**(6):820-30.
778. Mbuagbaw L, Van Der Kop ML, Lester RT, et al. Mobile phone text messages for improving adherence to antiretroviral therapy (ART): An individual patient data meta-analysis of randomised trials. BMJ Open 2013;**3**(12).

779. McAlister FA. Angiotensin-converting enzyme inhibitors or angiotensin receptor blockers are beneficial in normotensive atherosclerotic patients: a collaborative meta-analysis of randomized trials. Eur Heart J 2012;**33**(4):505-14.
780. McAlister FA, Ezekowitz J, Tarantini L, et al. Renal dysfunction in patients with heart failure with preserved versus reduced ejection fraction: impact of the new Chronic Kidney Disease-Epidemiology Collaboration Group formula. Circ Heart Fail 2012;**5**(3):309-14.
781. McCormack K, Wake B, Perez J, et al. Laparoscopic surgery for inguinal hernia repair: Systematic review of effectiveness and economic evaluation. Health Technol Assess 2005;**9**(14):iii-80.
782. McCullough PA, Bertrand ME, Brinker JA, et al. A Meta-Analysis of the Renal Safety of Isosmolar Iodixanol Compared With Low-Osmolar Contrast Media. J Am Coll Cardiol 2006;**48**(4):692-99.
783. McGee DL. Body mass index and mortality: a meta-analysis based on person-level data from twenty-six observational studies. Ann Epidemiol 2005;**15**(2):87-97.
784. McGeechan K, Liew G, Macaskill P, et al. Prediction of incident stroke events based on retinal vessel caliber: a systematic review and individual-participant meta-analysis. Am J Epidemiol 2009;**170**(11):1323-32.
785. McGeechan K, Liew G, Macaskill P, et al. Meta-analysis: retinal vessel caliber and risk for coronary heart disease. Ann Intern Med 2009;**151**(6):404-13.
786. McHugh GS, Engel DC, Butcher I, et al. Prognostic value of secondary insults in traumatic brain injury: Results from the IMPACT study. J Neurotrauma 2007;**24**(2):287-93.
787. McLernon DJ, Harrild K, Bergh C, et al. Clinical effectiveness of elective single versus double embryo transfer: meta-analysis of individual patient data from randomised trials. BMJ 2010;**341**:c6945.
788. Mehta SR, Boden WE, Eikelboom JW, et al. Antithrombotic therapy with fondaparinux in relation to interventional management strategy in patients with ST-and non-ST-segment elevation acute coronary syndromes an individual patient-level combined analysis of the fifth and sixth organization to assess strategies in ischemic syndromes (OASIS 5 and 6) randomized trials. Circulation 2008;**118**(20):2038-46.
789. Meijer A, Conradi HJ, Bos EH, et al. Adjusted prognostic association of depression following myocardial infarction with mortality and cardiovascular events: individual patient data meta-analysis. Br J Psychiatry 2013;**203**(2):90-102.
790. Meijer AB, O YL, Geleijns J, et al. Meta-analysis of 40- and 64-MDCT angiography for assessing coronary artery stenosis. AJR Am J Roentgenol 2008;**191**(6):1667-75.
791. Melichar B, Urminska H, Kohlova T, et al. Brain metastases of epithelial ovarian carcinoma responding to cisplatin and gemcitabine combination chemotherapy: a case report and review of the literature. [Review] [86 refs]. Gynecol Oncol 2004;**94**(2):267-76.
792. Melichar B, Voboril Z, Cerman JJ, et al. Hepatic arterial infusion chemotherapy in gastric cancer: a report of four cases and analysis of the literature. [Review] [41 refs]. Tumori 2004;**90**(4):428-34.
793. Melsen WG, Rovers MM, Groenwold RHH, et al. Attributable mortality of ventilator-associated pneumonia: a meta-analysis of individual patient data from randomised prevention studies. Lancet Infect Dis 2013;**13**(8):665-71.
794. Mercado N, Wijns W, Serruys PW, et al. One-year outcomes of coronary artery bypass graft surgery versus percutaneous coronary intervention with multiple stenting for multisystem disease: A meta-analysis of individual patient data from randomized clinical trials. J Thorac Cardiovasc Surg 2005;**130**(2):512-19.
795. Messori A, Vaiani M, Trippoli S, et al. Survival in patients with intermediate or high grade non-Hodgkin's lymphoma: meta-analysis of randomized studies comparing third generation regimens with CHOP. Br J Cancer 2001;**84**(3):303-7.
796. Meta-analysis Global Group in Chronic Heart Failure. The survival of patients with heart failure with preserved or reduced left ventricular ejection fraction: an individual patient data meta-analysis. Eur Heart J 2012;**33**(14):1750-7.
797. Meta-analysis Research Group, Moller JE, Whalley GA, et al. Independent prognostic importance of a restrictive left ventricular filling pattern after myocardial infarction: an individual patient meta-analysis: Meta-Analysis Research Group in Echocardiography acute myocardial infarction.[see comment]. Circulation 2008;**117**(20):2591-98.
798. Meta-analysis Research Group in Echocardiography (MeRGE) Heart Failure Collaborators. Independence of restrictive filling pattern and LV ejection fraction with mortality in heart failure: An individual patient meta-analysis. Eur J Heart Fail 2008;**10**(8):786-92.
799. Meyer-Baron M, Schaper M, Knapp G, et al. The neurobehavioral impact of manganese: results and challenges obtained by a meta-analysis of individual participant data. Neurotoxicology 2013;**36**:1-9.
800. Mhuircheartaigh RJN, Moore RA, McQuay HJ. Analysis of individual patient data from clinical trials: epidural morphine for postoperative pain. Br J Anaesth 2009;**103**(6):874-81.

801. Michiels S, Le Maitre A, Buyse M, et al. Surrogate endpoints for overall survival in locally advanced head and neck cancer: meta-analyses of individual patient data. Lancet Oncol 2009;**10**(4):341-50.
802. Middleton LJ, Champaneria R, Daniels JP, et al. Hysterectomy, endometrial destruction, and levonorgestrel releasing intrauterine system (Mirena) for heavy menstrual bleeding: systematic review and meta-analysis of data from individual patients. BMJ 2010;**341**:c3929.
803. Migliori GB, Sotgiu G, Gandhi NR, et al. Drug resistance beyond extensively drug-resistant tuberculosis: individual patient data meta-analysis. Eur Respir J 2013;**42**(1):169-79.
804. Miles DW, Dieras V, Cortes J, et al. First-line bevacizumab in combination with chemotherapy for HER2-negative metastatic breast cancer: pooled and subgroup analyses of data from 2447 patients. Ann Oncol 2013;**24**(11):2773-80.
805. Miller PD, Recker RR, Harris S, et al. Long-term fracture rates seen with continued ibandronate treatment: Pooled analysis of DIVA and MOBILE long-term extension studies. Osteoporos Int 2014;**25**(1):349-57.
806. Mills EJ, Lester R, Thorlund K, et al. Interventions to promote adherence to antiretroviral therapy in Africa: A network meta-analysis. Lancet HIV 2014;**1**(3):e104-e11.
807. Mismetti P, Quenet S, Levine M, et al. Enoxaparin in the treatment of deep vein thrombosis with or without pulmonary embolism: An individual patient data meta-analysis. Chest 2005;**128**(4):2203-10.
808. Misra P, Husain Q, Svider PF, et al. Management of sinonasal teratocarcinosarcoma: A systematic review. American Journal of Otolaryngology - Head and Neck Medicine and Surgery 2014;**35**(1):5-11.
809. Mistiaen PJ, Jolley DJ, McGowan S, et al. A multilevel analysis of three randomised controlled trials of the Australian Medical Sheepskin in the prevention of sacral pressure ulcers. Med J Aust 2010;**193**(11-12):638-41.
810. Mitry E, Douillard JY, Van Cutsem E, et al. Predictive factors of survival in patients with advanced colorectal cancer: an individual data analysis of 602 patients included in irinotecan phase III trials. Ann Oncol 2004;**15**(7):1013-7.
811. Mizoue T, Inoue M, Wakai K, et al. Alcohol drinking and colorectal cancer in Japanese: a pooled analysis of results from five cohort studies. Am J Epidemiol 2008;**167**(12):1397-406.
812. Mongraw-Chaffin ML, Peters SAE, Huxley RR, et al. The sex-specific association between BMI and coronary heart disease: A systematic review and meta-analysis of 95 cohorts with 1·2 million participants. Lancet Diabetes Endocrinol 2015;**3**(6):437-49.
813. Mons U, Muezzinler A, Gellert C, et al. Impact of smoking and smoking cessation on cardiovascular events and mortality among older adults: meta-analysis of individual participant data from prospective cohort studies of the CHANCES consortium. BMJ 2015;**350**:h1551.
814. Montalescot G, Antoniucci D, Kastrati A, et al. Abciximab in primary coronary stenting of ST-elevation myocardial infarction: a European meta-analysis on individual patients' data with long-term follow-up. Eur Heart J 2007;**28**(4):443-9.
815. Montravers P, Bassetti M, Dupont H, et al. Efficacy of tigecycline for the treatment of complicated skin and soft-tissue infections in real-life clinical practice from five European observational studies. J Antimicrob Chemother 2013;**68 Suppl 2**:ii15-24.
816. Moore RA, Cai N, Skljarevski V, et al. Duloxetine use in chronic painful conditions—Individual patient data responder analysis. European Journal of Pain 2014;**18**(1):67-75.
817. Moore RA, Edwards JE, McQuay HJ. Acute pain: individual patient meta-analysis shows the impact of different ways of analysing and presenting results. Pain 2005;**116**(3):322-31.
818. Moore RA, McQuay HJ. Single-patient data meta-analysis of 3453 postoperative patients: oral tramadol versus placebo, codeine and combination analgesics. Pain 1997;**69**(3):287-94.
819. Moore RA, Mhuircheartaigh RJN, Derry S, et al. Mean analgesic consumption is inappropriate for testing analgesic efficacy in post-operative pain: analysis and alternative suggestion. Eur J Anaesthesiol 2011;**28**(6):427-32.
820. Moore RA, Moore OA, Derry S, et al. Responder analysis for pain relief and numbers needed to treat in a meta-analysis of etoricoxib osteoarthritis trials: bridging a gap between clinical trials and clinical practice. Ann Rheum Dis 2010;**69**(2):374-9.
821. Moore RA, Straube S, Paine J, et al. Minimum efficacy criteria for comparisons between treatments using individual patient meta-analysis of acute pain trials: examples of etoricoxib, paracetamol, ibuprofen, and ibuprofen/paracetamol combinations after third molar extraction. Pain 2011;**152**(5):982-9.
822. Morita S, Okamoto I, Kobayashi K, et al. Combined survival analysis of prospective clinical trials of gefitinib for non-small cell lung cancer with EGFR mutations. Clin Cancer Res 2009;**15**(13):4493-8.
823. Morrison CS, Chen P-L, Kwok C, et al. Hormonal Contraception and the Risk of HIV Acquisition: An Individual Participant Data Meta-analysis. PLoS Med 2015;**12**(1):e1001778.

824. Morrone D, Weintraub WS, Toth PP, et al. Lipid-altering efficacy of ezetimibe plus statin and statin monotherapy and identification of factors associated with treatment response: a pooled analysis of over 21,000 subjects from 27 clinical trials. Atherosclerosis 2012;**223**(2):251-61.
825. Mosges R, Konig V, Koberlein J. The effectiveness of modern antihistamines for treatment of allergic rhinitis - an IPD meta-analysis of 140,853 patients. Allergology International 2013;**62**(2):215-22.
826. Moss A, Nicholas M. Language rehabilitation in chronic aphasia and time postonset: A review of single-subject data. Stroke 2006;**37**(12):3043-51.
827. Moss RL, Dimmitt RA, Henry MC, et al. A meta-analysis of peritoneal drainage versus laparotomy for perforated necrotizing enterocolitis. J Pediatr Surg 2001;**36**(8):1210-3.
828. Mou J, Paillard F, Turnbull B, et al. Qutenza (Capsaicin) 8% patch onset and duration of response and effects of multiple treatments in neuropathic pain patients. Clin J Pain 2014;**30**(4):286-94.
829. Mueller EA, van VM, Kirch W, et al. Efficacy and safety of the six-dose regimen of artemether-lumefantrine for treatment of uncomplicated Plasmodium falciparum malaria in adolescents and adults: A pooled analysis of individual patient data from randomized clinical trials. Acta Trop 2006;**100**(1-2):41-53.
830. Mulherin SA, O'Brien TR, Ioannidis JP, et al. Effects of CCR5-Delta32 and CCR2-64I alleles on HIV-1 disease progression: the protection varies with duration of infection.[see comment]. AIDS 2003;**17**(3):377-87.
831. Muller M, Marson AG, Williamson PR. Oxcarbazepine versus phenytoin monotherapy for epilepsy. [Review] [20 refs]. Cochrane Database Syst Rev 2006(2):CD003615-.
832. Mullins P, Sharplin P, Yki-Jarvinen H, et al. Negative binomial meta-regression analysis of combined glycosylated hemoglobin and hypoglycemia outcomes across eleven Phase III and IV studies of insulin glargine compared with neutral protamine Hagedorn insulin in type 1 and type 2 diabetes mellitus. Clin Ther 2007;**29**(8):1607-19.
833. Murakami Y. Meta-analyses using individual participant data from cardiovascular cohort studies in Japan: Current status and future directions. J Epidemiol 2014;**24**(2):96-101.
834. Murakami Y, Miura K, Okamura T, et al. Population attributable numbers and fractions of deaths due to smoking: a pooled analysis of 180,000 Japanese. Prev Med 2011;**52**(1):60-5.
835. Murray GD, Butcher I, McHugh GS, et al. Multivariable prognostic analysis in traumatic brain injury: Results from the IMPACT study. J Neurotrauma 2007;**24**(2):329-37.
836. Mushkudiani NA, Engel DC, Steyerberg EW, et al. Prognostic value of demographic characteristics in traumatic brain injury: Results from the IMPACT study. J Neurotrauma 2007;**24**(2):259-69.
837. Musso G, Gambino R, Tabibian JH, et al. Association of Non-alcoholic Fatty Liver Disease with Chronic Kidney Disease: A Systematic Review and Meta-analysis. PLoS Med 2014;**11**(7).
838. Muthuri SG, Venkatesan S, Myles PR, et al. Effectiveness of neuraminidase inhibitors in reducing mortality in patients admitted to hospital with influenza A H1N1pdm09 virus infection: A meta-analysis of individual participant data. Lancet Respir Med 2014;**2**(5):395-404.
839. Myeloma Trialists' Collaborative G. Interferon as therapy for multiple myeloma: an individual patient data overview of 24 randomized trials and 4012 patients. Br J Haematol 2001;**113**(4):1020-34.
840. Myeloma Trialists' Collaborative Group. Combination chemotherapy versus melphalan plus prednisone as treatment for multiple myeloma: an overview of 6,633 patients from 27 randomized trials. J Clin Oncol 1998;**16**(12):3832-42.
841. Naber KG, Niggemann H, Stein G, et al. Review of the literature and individual patients' data meta-analysis on efficacy and tolerance of nitroxoline in the treatment of uncomplicated urinary tract infections. BMC Infect Dis 2014;**14**:628.
842. Naghibi M, Smith TR, Elia M. A systematic review with meta-analysis of survival, quality of life and cost-effectiveness of home parenteral nutrition in patients with inoperable malignant bowel obstruction. Clin Nutr 2014.
843. Nakamura K, Barzi F, Huxley R, et al. Does cigarette smoking exacerbate the effect of total cholesterol and high-density lipoprotein cholesterol on the risk of cardiovascular diseases? Heart 2009;**95**(11):909-16.
844. Nakamura K, Barzi F, Lam TH, et al. Cigarette smoking, systolic blood pressure, and cardiovascular diseases in the Asia-Pacific region. Stroke 2008;**39**(6):1694-702.
845. Nakamura K, Kuwata T, Shimoda T, et al. Determination of the optimal cutoff percentage of residual tumors to define the pathological response rate for gastric cancer treated with preoperative therapy (JCOG1004-A). Gastric Cancer 2014.
846. Nan C, Guo B, Warner C, et al. Heritability of body mass index in pre-adolescence, young adulthood and late adulthood. Eur J Epidemiol 2012;**27**(4):247-53.
847. Naumann DN, Bhangu A, Kelly M, et al. Stapled versus handsewn intestinal anastomosis in emergency laparotomy: a systemic review and meta-analysis. Surgery 2015;**157**(4):609-18.

848. Neal B, MacMahon S, Chapman N, et al. Effects of ACE inhibitors, calcium antagonists, and other blood-pressure-lowering drugs: results of prospectively designed overviews of randomised trials. Blood Pressure Lowering Treatment Trialists' Collaboration. Lancet 2000;**356**(9246):1955-64.
849. Negri E, Dal Maso L, Ron E, et al. A pooled analysis of case-control studies of thyroid cancer¶ II. Menstrual and reproductive factors. Cancer Causes Control 1999;**10**(2):143-55.
850. Nelson HS, Chapman KR, Pyke SD, et al. Enhanced synergy between fluticasone propionate and salmeterol inhaled from a single inhaler versus separate inhalers.[see comment]. J Allergy Clin Immunol 2003;**112**(1):29-36.
851. Nelson JC. Anxiety does not predict response to duloxetine in major depression: results of a pooled analysis of individual patient data from 11 placebo-controlled trials. Depress Anxiety 2010;**27**(1):12-8.
852. Nelson JC, Delucchi KL, Schneider LS. Moderators of outcome in late-life depression: a patient-level meta-analysis. Am J Psychiatry 2013;**170**(6):651-9.
853. Neoadjuvant Chemotherapy for Cervical Cancer Meta-Analysis Collaboration NC. Neoadjuvant chemotherapy for locally advanced cervix cancer. [Review] [47 refs]. Cochrane Database Syst Rev 2004(2):CD001774.
854. Neuman A, Hohmann C, Orsini N, et al. Maternal smoking in pregnancy and asthma in preschool children: a pooled analysis of eight birth cohorts. Am J Respir Crit Care Med 2012;**186**(10):1037-43.
855. Newman DB, Fidahussein SS, Kashiwagi DT, et al. Reversible cardiac dysfunction associated with hypocalcemia: A systematic review and meta-analysis of individual patient data. Heart Fail Rev 2014;**19**(2):199-205.
856. Ni Mhurchu C, Rodgers A, Pan WH, et al. Body mass index and cardiovascular disease in the Asia-Pacific Region: an overview of 33 cohorts involving 310 000 participants. Int J Epidemiol 2004;**33**(4):751-8.
857. Nicolucci A, Carinci F, Graepel JG, et al. The efficacy of tolrestat in the treatment of diabetic peripheral neuropathy. A meta-analysis of individual patient data. Diabetes Care 1996;**19**(10):1091-6.
858. Nieder C, Grosu AL, Andratschke NH, et al. Proposal of human spinal cord reirradiation dose based on collection of data from 40 patients. [Review] [36 refs]. International Journal of Radiation Oncology, Biology, Physics 2005;**61**(3):851-5.
859. Nitsch D, Grams M, Sang Y, et al. Associations of estimated glomerular filtration rate and albuminuria with mortality and renal failure by sex: a meta-analysis. BMJ 2013;**346**:f324.
860. Nixon R, Bergvall N, Tomic D, et al. No evidence of disease activity: indirect comparisons of oral therapies for the treatment of relapsing-remitting multiple sclerosis. Adv Ther 2014;**31**(11):1134-54.
861. Nolan SJ, Marson AG, Pulman J, et al. Phenytoin versus valproate monotherapy for partial onset seizures and generalised onset tonic-clonic seizures. Cochrane Database Syst Rev 2013;**8**:CD001769.
862. Nolan SJ, Muller M, Tudur Smith C, et al. Oxcarbazepine versus phenytoin monotherapy for epilepsy. Cochrane Database Syst Rev 2013;**5**:CD003615.
863. Nolan SJ, Tudur Smith C, Pulman J, et al. Phenobarbitone versus phenytoin monotherapy for partial onset seizures and generalised onset tonic-clonic seizures. Cochrane Database Syst Rev 2013;**1**:CD002217.
864. Non-small Cell Lung Cancer Collaborative Group. Chemotherapy for non-small cell lung cancer. . Cochrane Database Syst Rev 2000(2):CD002139.
865. Non-Small Cell Lung Cancer Collaborative Group. Chemotherapy and supportive care versus supportive care alone for advanced non-small cell lung cancer. Cochrane Database Syst Rev 2010(5):CD007309.
866. Noordzij JP, Lee SL, Bernet VJ, et al. Early Prediction of Hypocalcemia after Thyroidectomy using Parathyroid Hormone: An Analysis of Pooled Individual Patient Data from Nine Observational Studies. J Am Coll Surg 2007;**205**(6):748-54.
867. Norman G, Soares M, Peura P, et al. Capecitabine for the treatment of advanced gastric cancer. Health technology assessment (Winchester, England) 2010;**14**(Suppl. 2):11-7.
868. Novara G, Chapple CR, Montorsi F. A pooled analysis of individual patient data from registrational trials of silodosin in the treatment of non-neurogenic male lower urinary tract symptoms (LUTS) suggestive of benign prostatic hyperplasia (BPH). BJU Int 2014.
869. Novara G, Chapple CR, Montorsi F. Individual patient data from registrational trials of silodosin in the treatment of non-neurogenic male lower urinary tract symptoms (LUTS) associated with benign prostatic hyperplasia (BPH): subgroup analyses of efficacy and safety data. BJU Int 2015;**115**(5):802-14.
870. nsary-Moghaddam A, Huxley R, Barzi F, et al. The effect of modifiable risk factors on pancreatic cancer mortality in populations of the Asia-Pacific region. Cancer Epidemiology Biomarkers and Prevention 2006;**15**(12):2435-40.
871. NSCLC Meta-analyses Collaborative Group, Arriagada R, Auperin A, et al. Adjuvant chemotherapy, with or without postoperative radiotherapy, in operable non-small-cell lung cancer: two meta-analyses of individual patient data. Lancet 2010;**375**(9722):1267-77.

872. NSCLC Meta-analysis Collaborative Group. Preoperative chemotherapy for non-small-cell lung cancer: a systematic review and meta-analysis of individual participant data. Lancet 2014;**383**(9928):1561-71.
873. Nyberg ST, Fransson EI, Heikkila K, et al. Job strain and cardiovascular disease risk factors: meta-analysis of individual-participant data from 47,000 men and women. PLoS ONE [Electronic Resource] 2013;**8**(6):e67323.
874. Nyberg ST, Heikkilä K, Fransson EI, et al. Job strain in relation to body mass index: pooled analysis of 160 000 adults from 13 cohort studies. J Intern Med 2012;**272**(1):65-73.
875. Nyström L, Wall S, Rutqvist L, et al. Breast cancer screening with mammography: overview of Swedish randomised trials. Lancet 1993;**341**(8851):973-78.
876. Oba K, Kobayashi M, Matsui T, et al. Individual patient based meta-analysis of lentinan for unresectable/recurrent gastric cancer. Anticancer Res 2009;**29**(7):2739-45.
877. Oba MS, Teramukai S, Ohashi Y, et al. The efficacy of adjuvant immunochemotherapy with OK-432 after curative resection of gastric cancer: an individual patient data meta-analysis of randomized controlled trials. Gastric Cancer 2015.
878. O'Connor SA, Morice MC, Gilard M, et al. Revisiting Sex Equality with Transcatheter Aortic Valve Replacement Outcomes A Collaborative, Patient-Level Meta-Analysis of 11,310 Patients. J Am Coll Cardiol 2015;**66**(3):221-28.
879. Offringa M, Bossuyt PM, Lubsen J, et al. Risk factors for seizure recurrence in children with febrile seizures: a pooled analysis of individual patient data from five studies. J Pediatr 1994;**124**(4):574-84.
880. Ohashi Y, Uemura Y, Fujisaka Y, et al. Meta-analysis of epoetin beta and darbepoetin alfa treatment for chemotherapy-induced anemia and mortality: Individual patient data from Japanese randomized, placebo-controlled trials. Cancer Sci 2013;**104**(4):481-5.
881. Ohashi Y, Watanabe T, Sano M, et al. Efficacy of oral tegafur-uracil (UFT) as adjuvant therapy as compared with classical cyclophosphamide, methotrexate, and 5-fluorouracil (CMF) in early breast cancer: a pooled analysis of two randomized controlled trials (N.SAS-BC 01 trial and CUBC trial). Breast Cancer Res Treat 2010;**119**(3):633-41.
882. Olsen CM, Zens MS, Green AC, et al. Biologic markers of sun exposure and melanoma risk in women: pooled case-control analysis. Int J Cancer 2011;**129**(3):713-23.
883. O'Meara S, Cullum N, Nelson EA, et al. Compression for venous leg ulcers. Cochrane Database Syst Rev 2012;**11**:CD000265.
884. O'Neill ID. Efficacy of tumour necrosis factor-alpha antagonists in aphthous ulceration: review of published individual patient data. J Eur Acad Dermatol Venereol 2012;**26**(2):231-5.
885. Ong KT, Delerme S, Pannier B, et al. Aortic stiffness is reduced beyond blood pressure lowering by short-term and long-term antihypertensive treatment: a meta-analysis of individual data in 294 patients. J Hypertens 2011;**29**(6):1034-42.
886. Opdam NJM, van de Sande FH, Bronkhorst E, et al. Longevity of posterior composite restorations: a systematic review and meta-analysis. J Dent Res 2014;**93**(10):943-9.
887. Orditura M, Galizia G, Morgillo F, et al. Complete response to preoperative chemoradiation and survival in esophageal cancer: a pooled analysis of three single-institution phase II trials. Dis Esophagus 2012;**25**(2):130-6.
888. Ordóñez-Mena JM, Schöttker B, Fedirko V, et al. Pre-diagnostic vitamin D concentrations and cancer risks in older individuals: an analysis of cohorts participating in the CHANCES consortium. Eur J Epidemiol 2015.
889. Ossenkoppele R, Jansen WJ, Rabinovici GD, et al. Prevalence of amyloid PET positivity in dementia syndromes: a meta-analysis. JAMA 2015;**313**(19):1939-49.
890. Oudshoorn SC, Rinkel GJE, Molyneux AJ, et al. Aneurysm Treatment <24 Versus 24-72 h After Subarachnoid Hemorrhage. Neurocrit Care 2014.
891. Padwal R, McAlister FA, McMurray JJV, et al. The obesity paradox in heart failure patients with preserved versus reduced ejection fraction: a meta-analysis of individual patient data. Int J Obes 2014;**38**(8):1110-4.
892. Pagliaro L, Craxi A, Cammaa C, et al. Interferon-alpha for chronic hepatitis C: an analysis of pretreatment clinical predictors of response. Hepatology 1994;**19**(4):820-8.
893. Palma DA, Senan S, Oberije C, et al. Predicting esophagitis after chemoradiation therapy for non-small cell lung cancer: an individual patient data meta-analysis. International Journal of Radiation Oncology, Biology, Physics 2013;**87**(4):690-6.
894. Palma DA, Senan S, Tsujino K, et al. Predicting radiation pneumonitis after chemoradiation therapy for lung cancer: an international individual patient data meta-analysis. International Journal of Radiation Oncology, Biology, Physics 2013;**85**(2):444-50.

895. Palmerini T, Sangiorgi D, Valgimigli M, et al. Short- versus long-term dual antiplatelet therapy after drug-eluting stent implantation: an individual patient data pairwise and network meta-analysis. J Am Coll Cardiol 2015;**65**(11):1092-102.
896. Palumbo A, Bringhen S, Kumar SK, et al. Second primary malignancies with lenalidomide therapy for newly diagnosed myeloma: a meta-analysis of individual patient data. Lancet Oncol 2014;**15**(3):333-42.
897. Palumbo A, Waage A, Hulin C, et al. Safety of thalidomide in newly diagnosed elderly myeloma patients: a meta-analysis of data from individual patients in six randomized trials. Haematologica 2013;**98**(1):87-94.
898. Pandor A, Thokala P, Goodacre S, et al. Pre-hospital non-invasive ventilation for acute respiratory failure: A systematic review and cost-effectiveness evaluation. Health Technol Assess 2015;**19**(42):1-8.
899. Papakostas GI, Montgomery SA, Thase ME, et al. Comparing the rapidity of response during treatment of major depressive disorder with bupropion and the SSRIs: A pooled survival analysis of 7 double-blind, randomized clinical trials. J Clin Psychiatry 2007;**68**(12):1907-12.
900. Papakostas GI, Trivedi MH, Alpert JE, et al. Efficacy of bupropion and the selective serotonin reuptake inhibitors in the treatment of anxiety symptoms in major depressive disorder: A meta-analysis of individual patient data from 10 double-blind, randomized clinical trials. J Psychiatr Res 2008;**42**(2):134-40.
901. Park D-W, Kim Y-H, Song HG, et al. Long-term outcome of stents versus bypass surgery in diabetic and nondiabetic patients with multivessel or left main coronary artery disease: a pooled analysis of 5775 individual patient data. Circ Cardiovasc Interv 2012;**5**(4):467-75.
902. Park D-W, Kim Y-H, Yun S-C, et al. Frequency, causes, predictors, and clinical significance of peri-procedural myocardial infarction following percutaneous coronary intervention. Eur Heart J 2013;**34**(22):1662-9.
903. Pastula DM, Moore DH, Bedlack RS. Creatine for amyotrophic lateral sclerosis/motor neuron disease. Cochrane Database Syst Rev 2012;**12**:CD005225.
904. Patel A, Barzi F, Jamrozik K, et al. Serum triglycerides as a risk factor for cardiovascular diseases in the Asia-Pacific region. Circulation 2004;**110**(17):2678-86.
905. Patel KV, Semba RD, Ferrucci L, et al. Red cell distribution width and mortality in older adults: a meta-analysis. Journals of Gerontology Series A-Biological Sciences and Medical Sciences 2010;**65**(3):258-65.
906. Patra J, Jha P, Rehm J, et al. Tobacco smoking, alcohol drinking, diabetes, low body mass index and the risk of self-reported symptoms of active tuberculosis: Individual Participant Data (IPD) meta-analyses of 72,684 individuals in 14 high tuberculosis burden countries. PLoS ONE [Electronic Resource] 2014;**9**(5).
907. Patti G, Cannon CP, Murphy SA, et al. Clinical benefit of statin pretreatment in patients undergoing percutaneous coronary intervention: A collaborative patient-level meta-analysis of 13 randomized studies. Circulation 2011;**123**(15):1622-32.
908. Pavlov CS, Casazza G, Nikolova D, et al. Transient elastography for diagnosis of stages of hepatic fibrosis and cirrhosis in people with alcoholic liver disease. Cochrane Database Syst Rev 2015;**1**.
909. Pawinski A, Sylvester R, Kurth KH, et al. A combined analysis of European Organization for Research and Treatment of Cancer, and Medical Research Council randomized clinical trials for the prophylactic treatment of stage TaT1 bladder cancer. European Organization for Research and Treatment of Cancer Genitourinary Tract Cancer Cooperative Group and the Medical Research Council Working Party on Superficial Bladder Cancer. J Urol 1996;**156**(6):1934-40.
910. Pearce CL, Templeman C, Rossing MA, et al. Association between endometriosis and risk of histological subtypes of ovarian cancer: a pooled analysis of case-control studies. Lancet Oncol 2012;**13**(4):385-94.
911. Pearse RM, Belsey JD, Cole JN, et al. Effect of dopexamine infusion on mortality following major surgery: Individual patient data meta-regression analysis of published clinical trials. Crit Care Med 2008;**36**(4):1323-29.
912. Peeters M, Kafatos G, Taylor A, et al. Prevalence of RAS mutations and individual variation patterns among patients with metastatic colorectal cancer: A pooled analysis of randomised controlled trials. Eur J Cancer 2015.
913. Peinemann F, Smith LA, Bartel C. Autologous hematopoietic stem cell transplantation following high dose chemotherapy for non-rhabdomyosarcoma soft tissue sarcomas. Cochrane Database Syst Rev 2013;**8**:CD008216.
914. Pereira TV, Kimura L, Suwazono Y, et al. Multivariate meta-analysis of the association of G-protein beta 3 gene (GNB3) haplotypes with cardiovascular phenotypes. Mol Biol Rep 2014:1-13.
915. Peter I, Crosier MD, Yoshida M, et al. Associations of APOE gene polymorphisms with bone mineral density and fracture risk: a meta-analysis. Osteoporos Int 2011;**22**(4):1199-209.
916. Peters AL, Davidson MB, Schriger DL, et al. A clinical approach for the diagnosis of diabetes mellitus: an analysis using glycosylated hemoglobin levels. JAMA 1996;**276**(15):1246-52.

917. Peters SA, Woodward M, Lam TH, et al. Sex disparities in risk and risk factors for ischemic heart disease in the Asia-Pacific region. European Journal of Preventive Cardiology 2014;**21**(5):639-46.
918. Pettengell R, Schwenkglenks M, Bacon P, et al. Pegfilgrastim primary prophylaxis in patients with non-Hodgkin lymphoma: results from an integrated analysis. Hematol Oncol 2011;**29**(4):177-84.
919. Pfister R, Kochanek M, Leygeber T, et al. Procalcitonin for diagnosis of bacterial pneumonia in critically ill patients during 2009 H1N1 influenza pandemic: A prospective cohort study, systematic review and individual patient data meta-analysis. Critical Care 2014;**18**(2).
920. Phi X-A, Houssami N, Obdeijn I-M, et al. Magnetic resonance imaging improves breast screening sensitivity in BRCA mutation carriers age > 50 years: evidence from an individual patient data meta-analysis. J Clin Oncol 2015;**33**(4):349-56.
921. Piaggio G, Heng Z, von Hertzen H, et al. Combined estimates of effectiveness of mifepristone 10 mg in emergency contraception. Contraception 2003;**68**(6):439-46.
922. Piccart-Gebhart MJ, Burzykowski T, Buyse M, et al. Taxanes alone or in combination with anthracyclines as first-line therapy of patients with metastatic breast cancer. J Clin Oncol 2008;**26**(12):1980-86.
923. Piccolo R, Eitel I, Iversen AZ, et al. Intracoronary versus intravenous bolus abciximab administration in patients undergoing primary percutaneous coronary intervention with acute ST-elevation myocardial infarction: A pooled analysis of individual patient data from five randomised controlled trials. EuroIntervention 2014;**9**(9):1110-20.
924. Pickup JC, Freeman SC, Sutton AJ. Glycaemic control in type 1 diabetes during real time continuous glucose monitoring compared with self monitoring of blood glucose: meta-analysis of randomised controlled trials using individual patient data. BMJ 2011;**343**:d3805.
925. Piedbois P, Buyse M, Kemeny N, et al. Reappraisal of hepatic arterial infusion in the treatment of nonresectable liver metastases from colorectal cancer. J Natl Cancer Inst 1996;**88**(5):252-8.
926. Piedbois P, Rougier P, Buyse M, et al. Efficacy of intravenous continuous infusion of fluorouracil compared with bolus administration in advanced colorectal cancer. Journal of clinical oncology: official journal of the American Society of Clinical Oncology 1998;**16**(1):301-08.
927. Piepoli MF, Davos C, Francis DP, et al. Exercise training meta-analysis of trials in patients with chronic heart failure (ExTraMATCH).[see comment]. BMJ 2004;**328**(7433):189.
928. Pietschmann S, von Bueren AO, Henke G, et al. An individual patient data meta-analysis on characteristics, treatments and outcomes of the glioblastoma/gliosarcoma patients with central nervous system metastases reported in literature until 2013. J Neurooncol 2014.
929. Pietschmann S, Von Bueren AO, Kerber MJ, et al. An individual patient data meta-analysis on characteristics, treatments and outcomes of glioblastoma/ gliosarcoma patients with metastases outside of the central nervous system. PLoS One 2015;**10**(4).
930. Pignon J, Bourhis J, Domenge Co, et al. Chemotherapy added to locoregional treatment for head and neck squamous-cell carcinoma: three meta-analyses of updated individual data. Lancet 2000;**355**(9208):949-55.
931. Pignon J-P, Arriagada R, Ihde DC, et al. A meta-analysis of thoracic radiotherapy for small-cell lung cancer. N Engl J Med 1992;**327**(23):1618-24.
932. Pignon J-P, le Maitre A, Maillard E, et al. Meta-analysis of chemotherapy in head and neck cancer (MACH-NC): an update on 93 randomised trials and 17,346 patients. Radiother Oncol 2009;**92**(1):4-14.
933. Pignon J-P, Tribodet H, Scagliotti GV, et al. Lung adjuvant cisplatin evaluation: A pooled analysis by the LACE collaborative group. J Clin Oncol 2008;**26**(21):3552-59.
934. Pocock SJ, Lansky AJ, Mehran R, et al. Angiographic Surrogate End Points in Drug-Eluting Stent Trials. A Systematic Evaluation Based on Individual Patient Data From 11 Randomized, Controlled Trials. J Am Coll Cardiol 2008;**51**(1):23-32.
935. Pocock SJ, McCormack V, Gueyffier F, et al. A score for predicting risk of death from cardiovascular disease in adults with raised blood pressure, based on individual patient data from randomised controlled trials. BMJ 2001;**323**(7304):75-81.
936. Pollack MH, Endicott J, Liebowitz M, et al. Examining quality of life in patients with generalized anxiety disorder: Clinical relevance and response to duloxetine treatment. J Psychiatr Res 2008;**42**(12):1042-49.
937. Pond GR, Agarwal N, Bellmunt J, et al. A nomogram including baseline prognostic factors to estimate the activity of second-line therapy for advanced urothelial carcinoma. BJU Int 2014;**113**(5b):E137-43.
938. Porter CK, Riddle MS, Tribble DR, et al. A systematic review of experimental infections with enterotoxigenic Escherichia coli (ETEC). Vaccine 2011;**29**(35):5869-85.
939. Potgieter D, Simmers D, Ryan L, et al. N-terminal pro-B-type Natriuretic Peptides' Prognostic Utility Is Overestimated in Meta-analyses Using Study-specific Optimal Diagnostic Thresholds. Anesthesiology 2015;**123**(2):264-71.

940. Poynard T, Cales P, Pasta L, et al. Beta-adrenergic-antagonist drugs in the prevention of gastrointestinal bleeding in patients with cirrhosis and esophageal varices. An analysis of data and prognostic factors in 589 patients from four randomized clinical trials. Franco-Italian Multicenter Study Group. N Engl J Med 1991;**324**(22):1532-8.
941. Poynard T, Lassailly G, Diaz E, et al. Performance of biomarkers FibroTest, ActiTest, SteatoTest, and NashTest in patients with severe obesity: meta analysis of individual patient data. PLoS ONE [Electronic Resource] 2012;**7**(3):e30325.
942. Poynard T, McHutchison J, Davis GL, et al. Impact of interferon alfa-2b and ribavirin on progression of liver fibrosis in patients with chronic hepatitis C. Hepatology 2000;**32**(5):1131-37.
943. Poynard T, Munteanu M, Ngo Y, et al. ActiTest accuracy for the assessment of histological activity grades in patients with chronic hepatitis C, an overview using Obuchowski measure. Gastroenterol Clin Biol 2010;**34**(6-7):388-96.
944. Preference Collaborative Review Group. Patients' preferences within randomised trials: systematic review and patient level meta-analysis. BMJ 2008;**337**:a1864.
945. Presson A, Moore TB, Kempert P. Efficacy of high-dose chemotherapy and autologous stem cell transplant for recurrent Wilms' tumor: a meta-analysis. J Pediatr Hematol Oncol 2010;**32**(6):454-61.
946. Prodromou ML, Ziakas PD, Poulou LS, et al. FDG PET is a robust tool for the diagnosis of spondylodiscitis: A meta-analysis of diagnostic data. Clin Nucl Med 2014;**39**(4):330-35.
947. Prophylactic Cranial Irradiation Overview Collaborative Group. Cranial irradiation for preventing brain metastases of small cell lung cancer in patients in complete remission. Cochrane Database Syst Rev 2000;**4**.
948. Prostate Cancer Trialists' Collaborative Group. Maximum androgen blockade in advanced prostate cancer: an overview of 22 randomised trials with 3283 deaths in 5710 patients. . Lancet 1995;**346**(8970):265-9.
949. Proulx NL, Akbari A, Garg AX, et al. Measured creatinine clearance from timed urine collections substantially overestimates glomerular filtration rate in patients with liver cirrhosis: A systematic review and individual patient meta-analysis. Nephrology Dialysis Transplantation 2005;**20**(8):1617-22.
950. Prowle JR, Ishikawa K, May CN, et al. Renal plasma flow and glomerular filtration rate during acute kidney injury in man. Ren Fail 2010;**32**(3):349-55.
951. Pujol JL, Pirker R, Lynch TJ, et al. Meta-analysis of individual patient data from randomized trials of chemotherapy plus cetuximab as first-line treatment for advanced non-small cell lung cancer. Lung Cancer 2014;**83**(2):211-18.
952. Qizilbash N, Whitehead A, Higgins J, et al. Cholinesterase inhibition for Alzheimer disease: a meta-analysis of the tacrine trials. Dementia Trialists' Collaboration.[see comment]. JAMA 1998;**280**(20):1777-82.
953. Quan A, Kerlikowske K, Gueyffier F, et al. Efficacy of treating hypertension in women. J Gen Intern Med 1999;**14**(12):718-29.
954. Quarles van Ufford HME, van Tinteren H, Stroobants SG, et al. Added value of baseline 18F-FDG uptake in serial 18F-FDG PET for evaluation of response of solid extracerebral tumors to systemic cytotoxic neoadjuvant treatment: a meta-analysis. J Nucl Med 2010;**51**(10):1507-16.
955. Quinten C, Coens C, Mauer M, et al. Baseline quality of life as a prognostic indicator of survival: a meta-analysis of individual patient data from EORTC clinical trials. Lancet Oncol 2009;**10**(9):865-71.
956. Rabinowitz J, Werbeloff N, Caers I, et al. Determinants of antipsychotic response in schizophrenia: Implications for practice and future clinical trials. J Clin Psychiatry 2014;**75**(4):e308-e16.
957. Raboud J, Montaner J, Rae S, et al. Meta-analysis of five randomized controlled trials comparing continuation of zidovudine versus switching to didanosine in HIV-infected individuals. Antivir Ther 1997;**2**(4):237-47.
958. Raboud JM, Diong C, Carr A, et al. A meta-analysis of six placebo-controlled trials of thiazolidinedione therapy for HIV lipoatrophy. HIV Clin Trials 2010;**11**(1):39-50.
959. Raboud JM, Rae S, Montaner JS. Predicting HIV RNA virologic outcome at 52-weeks follow-up in antiretroviral clinical trials. The INCAS and AVANTI Study Groups. Journal of Acquired Immune Deficiency Syndromes: JAIDS 2000;**24**(5):433-9.
960. Raboud JM, Rae S, Vella S, et al. Meta-analysis of two randomized controlled trials comparing combined zidovudine and didanosine therapy with combined zidovudine, didanosine, and nevirapine therapy in patients with HIV. INCAS study team. Journal of Acquired Immune Deficiency Syndromes: JAIDS 1999;**22**(3):260-6.
961. Raftopoulos Y, Senagore AJ, Di GG, et al. Recurrence rates after abdominal surgery for complete rectal prolapse: A multicenter pooled analysis of 643 individual patient data. Dis Colon Rectum 2005;**48**(6):1200-06.

962. Rahman A, Rizwan S, Waycaster C, et al. Pooled analysis of two clinical trials comparing the clinical outcomes of topical ciprofloxacin/dexamethasone otic suspension and polymyxin B/neomycin/hydrocortisone otic suspension for the treatment of acute otitis externa in adults and children. Clin Ther 2007;**29**(9):1950-6.
963. Raja FA, Counsell N, Colombo N, et al. Platinum versus platinum-combination chemotherapy in platinum-sensitive recurrent ovarian cancer: A meta-analysis using individual patient data. Ann Oncol 2013;**24**(12):3028-34.
964. Ramey DR, Watson DJ, Yu C, et al. The incidence of upper gastrointestinal adverse events in clinical trials of etoricoxib vs non-selective NSAIDs: An updated combined analysis. Curr Med Res Opin 2005;**21**(5):715-22.
965. Rand WM, Pellett PL, Young VR. Meta-analysis of nitrogen balance studies for estimating protein requirements in healthy adults. Am J Clin Nutr 2003;**77**(1):109-27.
966. Ratner R, Han J, Nicewarner D, et al. Cardiovascular safety of exenatide BID: an integrated analysis from controlled clinical trials in participants with type 2 diabetes. Cardiovasc Diabetol 2011;**10**:22.
967. Ravaud A, Urva SR, Grosch K, et al. Relationship between everolimus exposure and safety and efficacy: Meta-analysis of clinical trials in oncology. Eur J Cancer 2014;**50**(3):486-95.
968. Ravn A-M, Gregersen NT, Christensen R, et al. Thermic effect of a meal and appetite in adults: an individual participant data meta-analysis of meal-test trials. Food and Nutrition Research 2013;**57**.
969. Read JS. Mortality among HIV-1-infected women according to children's feeding modality: An individual patient data meta-analysis. J Acquir Immune Defic Syndr 2005;**39**(4):430-38.
970. Regan MM, O'Donnell EK, Kelly WK, et al. Efficacy of carboplatin-taxane combinations in the management of castration-resistant prostate cancer: a pooled analysis of seven prospective clinical trials. Ann Oncol 2010;**21**(2):312-8.
971. Reigner B, Welker H. Factors influencing elimination and distribution of fleroxacin: metaanalysis of individual data from 10 pharmacokinetic studies. Antimicrob Agents Chemother 1996;**40**(3):575-80.
972. Rejnmark L, Avenell A, Masud T, et al. Vitamin D with calcium reduces mortality: patient level pooled analysis of 70,528 patients from eight major vitamin D trials. J Clin Endocrinol Metab 2012;**97**(8):2670-81.
973. Renfro LA, Grothey A, Kerr D, et al. Survival following early-stage colon cancer: An ACCENT-based comparison of patients versus a matched international general population. Ann Oncol 2015;**26**(5):950-58.
974. Rerkasem K, Rothwell PM. Carotid endarterectomy for symptomatic carotid stenosis. Cochrane Database Syst Rev 2011(4):CD001081.
975. Rhee S-Y, Blanco JL, Jordan MR, et al. Geographic and Temporal Trends in the Molecular Epidemiology and Genetic Mechanisms of Transmitted HIV-1 Drug Resistance: An Individual-Patient- and Sequence-Level Meta-Analysis. PLoS Med 2015;**12**(4):e1001810.
976. Richards S, Pui C-H, Gayon P, et al. Systematic review and meta-analysis of randomized trials of central nervous system directed therapy for childhood acute lymphoblastic leukemia. Pediatric Blood and Cancer 2013;**60**(2):185-95.
977. Risch N, Herrell R, Lehner T, et al. Interaction between the serotonin transporter gene (5-HTTLPR), stressful life events, and risk of depression: a meta-analysis. JAMA 2009;**301**(23):2462-71.
978. Rizk S, Robert A, Vandenhooft A, et al. Activity of chemotherapy in the palliative treatment of salivary gland tumors: review of the literature. Eur Arch Otorhinolaryngol 2007;**264**(6):587-94.
979. Roche H, Conte P, Perez EA, et al. Ixabepilone plus capecitabine in metastatic breast cancer patients with reduced performance status previously treated with anthracyclines and taxanes: a pooled analysis by performance status of efficacy and safety data from 2 phase III studies. Breast Cancer Res Treat 2011;**125**(3):755-65.
980. Roddam AW, Allen NE, Appleby P, et al. Endogenous sex hormones and prostate cancer: a collaborative analysis of 18 prospective studies. J Natl Cancer Inst 2008;**100**(3):170-83.
981. Roddam AW, Allen NE, Appleby P, et al. Insulin-like growth factors, their binding proteins, and prostate cancer risk: Analysis of individual patient data from 12 prospective studies. Ann Intern Med 2008;**149**(7):461-71.
982. Rodondi N, Maisonneuve P, Razvi S, et al. Subclinical Hypothyroidism and the Risk of Coronary Heart Disease and Mortality: An Individual Participant Data Analysis from Nine Prospective Cohort Studies. J Gen Intern Med 2010;**25**:394-95.
983. Rodrigo GJ, Neffen H. Assessment of acute asthma severity in the ED: Are heart and respiratory rates relevant? Am J Emerg Med 2015.
984. Rodseth RN, Biccard BM, Chu R, et al. Postoperative B-type natriuretic peptide for prediction of major cardiac events in patients undergoing noncardiac surgery: systematic review and individual patient meta-analysis. Anesthesiology 2013;**119**(2):270-83.

985. Rodseth RN, Biccard BM, Le Manach Y, et al. The prognostic value of pre-operative and post-operative B-type natriuretic peptides in patients undergoing noncardiac surgery: B-type natriuretic peptide and N-terminal fragment of pro-B-type natriuretic peptide: a systematic review and individual patient data meta-analysis. J Am Coll Cardiol 2014;**63**(2):170-80.
986. Rodseth RN, Lurati Buse GA, Bolliger D, et al. The predictive ability of pre-operative B-type natriuretic peptide in vascular patients for major adverse cardiac events: an individual patient data meta-analysis. J Am Coll Cardiol 2011;**58**(5):522-9.
987. Rogers S, McIntosh RL, Cheung N, et al. The prevalence of retinal vein occlusion: pooled data from population studies from the United States, Europe, Asia, and Australia. Ophthalmology 2010;**117**(2):313-9.e1.
988. Rohatiner AZ, Gregory WM, Peterson B, et al. Meta-analysis to evaluate the role of interferon in follicular lymphoma. J Clin Oncol 2005;**23**(10):2215-23.
989. Rojas MP, Telaro E, Russo A, et al. Follow-up strategies for women treated for early breast cancer.[update of Cochrane Database Syst Rev. 2000;(4):CD001768; PMID: 11034727]. [Review] [39 refs]. Cochrane Database Syst Rev 2005(1):CD001768.
990. Roman E, Fear NT, Ansell P, et al. Vitamin K and childhood cancer: analysis of individual patient data from six case-control studies. Br J Cancer 2002;**86**(1):63-9.
991. Romero R, Nicolaides K, Conde-Agudelo A, et al. Vaginal progesterone in women with an asymptomatic sonographic short cervix in the midtrimester decreases preterm delivery and neonatal morbidity: a systematic review and metaanalysis of individual patient data. Am J Obstet Gynecol 2012;**206**(2):124.e1-19.
992. Ronellenfitsch U, Schwarzbach M, Hofheinz R, et al. Perioperative chemo(radio)therapy versus primary surgery for resectable adenocarcinoma of the stomach, gastroesophageal junction, and lower esophagus. Cochrane Database Syst Rev 2013;**5**:CD008107.
993. Roseann AE, Koru-Sengul T, Linkins L, et al. Differences in clinical presentation of deep vein thrombosis in men and women. J Thromb Haemost 2008;**6**(10):1713-19.
994. Rosenfeld E, Beyerlein A, Hadders-Algra M, et al. IPD meta-analysis shows no effect of LC-PUFA supplementation on infant growth at 18 months. Acta Paediatr 2009;**98**(1):91-97.
995. Rosner S, Hackl-Herrwerth A, Leucht S, et al. Acamprosate for alcohol dependence. Cochrane Database Syst Rev 2010(9):CD004332.
996. Rossi A, Chiodini P, Sun J-M, et al. Six versus fewer planned cycles of first-line platinum-based chemotherapy for non-small-cell lung cancer: a systematic review and meta-analysis of individual patient data. Lancet Oncol 2014;**15**(11):1254-62.
997. Rossi A, Di Maio M, Chiodini P, et al. Carboplatin- or cisplatin-based chemotherapy in first-line treatment of small-cell lung cancer: the COCIS meta-analysis of individual patient data. J Clin Oncol 2012;**30**(14):1692-8.
998. Rothwell PM, Fowkes FGR, Belch JFF, et al. Effect of daily aspirin on long-term risk of death due to cancer: analysis of individual patient data from randomised trials. Lancet 2011;**377**(9759):31-41.
999. Rothwell PM, Price JF, Fowkes FGR, et al. Short-term effects of daily aspirin on cancer incidence, mortality, and non-vascular death: analysis of the time course of risks and benefits in 51 randomised controlled trials. Lancet 2012;**379**(9826):1602-12.
1000. Rothwell PM, Wilson M, Elwin C-E, et al. Long-term effect of aspirin on colorectal cancer incidence and mortality: 20-year follow-up of five randomised trials. Lancet 2010;**376**(9754):1741-50.
1001. Rovers MM, Black N, Browning GG, et al. Grommets in otitis media with effusion: an individual patient data meta-analysis. [Review] [32 refs]. Arch Dis Child 2005;**90**(5):480-5.
1002. Rovers MM, Glasziou P, Appelman CL, et al. Antibiotics for acute otitis media: a meta-analysis with individual patient data. Lancet 2006;**368**(9545):1429-35.
1003. Rovers MM, Glasziou P, Appelman CL, et al. Predictors of pain and/or fever at 3 to 7 days for children with acute otitis media not treated initially with antibiotics: A meta-analysis of individual patient data. Pediatrics 2007;**119**(3):579-85.
1004. Ruijs ACJ, Jaquet J-B, Kalmijn S, et al. Median and ulnar nerve injuries: A meta-analysis of predictors of motor and sensory recovery after modern microsurgical nerve repair. Plast Reconstr Surg 2005;**116**(2):484-94.
1005. Russ TC, Stamatakis E, Hamer M, et al. Association between psychological distress and mortality: individual participant pooled analysis of 10 prospective cohort studies. BMJ 2012;**345**:e4933.
1006. Russ TC, Stamatakis E, Hamer M, et al. Socioeconomic status as a risk factor for dementia death: individual participant meta-analysis of 86 508 men and women from the UK. Br J Psychiatry 2013;**203**(1):10-7.

1007. Russell JM, Weisberg R, Fava M, et al. Efficacy of duloxetine in the treatment of generalized anxiety disorder in patients with clinically significant pain symptoms. Depress Anxiety 2008;**25**(7):E1-11.
1008. Sabaté M, Räber L, Heg D, et al. Comparison of newer-generation drug-eluting with bare-metal stents in patients with acute ST-segment elevation myocardial infarction: A pooled analysis of the EXAMINATION (clinical evaluation of the xience-V stent in acute myocardial INfArcTION) and COMFORTABLE-AMI. JACC Cardiovasc Interv 2014;**7**(1):55-63.
1009. Saber W, Moua T, Williams EC, et al. Risk factors for catheter-related thrombosis (CRT) in cancer patients: a patient-level data (IPD) meta-analysis of clinical trials and prospective studies. J Thromb Haemost 2011;**9**(2):312-9.
1010. Saccone G, Rust O, Althuisius S, et al. Cerclage for short cervix in twin pregnancies: systematic review and meta-analysis of randomized trials using individual patient-level data. Acta Obstet Gynecol Scand 2015;**94**(4):352-8.
1011. Sadigh G, Carlos RC, Neal CH, et al. Impact of breast mass size on accuracy of ultrasound elastography vs. conventional B-mode ultrasound: a meta-analysis of individual participants. Eur Radiol 2013;**23**(4):1006-14.
1012. Sahgal A, Aoyama H, Kocher M, et al. Phase 3 trials of stereotactic radiosurgery with or without whole-brain radiation therapy for 1 to 4 brain metastases: individual patient data meta-analysis. International Journal of Radiation Oncology, Biology, Physics 2015;**91**(4):710-7.
1013. Sakamoto J, Hamada C, Kodaira S, et al. Adjuvant therapy with oral fluoropyrimidines as main chemotherapeutic agents after curative resection for colorectal cancer: individual patient data meta-analysis of randomized trials. Jpn J Clin Oncol 1999;**29**(2):78-86.
1014. Sakamoto J, Hamada C, Rahman M, et al. An individual patient data meta-analysis of adjuvant therapy with carmofur in patients with curatively resected colon cancer. Jpn J Clin Oncol 2005;**35**(9):536-44.
1015. Sakamoto J, Hamada C, Yoshida S, et al. An individual patient data meta-analysis of adjuvant therapy with uracil-tegafur (UFT) in patients with curatively resected rectal cancer. Br J Cancer 2007;**96**(8):1170-77.
1016. Sakamoto J, Kodaira S, Hamada C, et al. An individual patient data meta-analysis of long supported adjuvant chemotherapy with oral carmofur in patients with curatively resected colorectal cancer. Oncol Rep 2001;**8**(3):697-703.
1017. Sakamoto J, Ohashi Y, Hamada C, et al. Efficacy of oral adjuvant therapy after resection of colorectal cancer: 5-year results from three randomized trials. J Clin Oncol 2004;**22**(3):484-92.
1018. Sakamoto J, Teramukai S, Koike A, et al. Prognostic value of preoperative immunosuppressive acidic protein in patients with gastric carcinoma. Findings from three independent clinical trials. Tumor Marker Committee for the Study Group of Immunochemotherapy with PSK for Gastric Cancer. Cancer 1996;**77**(11):2206-12.
1019. Salah K, Kok WE, Eurlings LW, et al. A novel discharge risk model for patients hospitalised for acute decompensated heart failure incorporating N-terminal pro-B-type natriuretic peptide levels: a European coLlaboration on Acute decompeNsated Heart Failure: ELAN-HF Score. Heart 2014;**100**(2):115-25.
1020. Salah K, Pinto YM, Eurlings LW, et al. Serum potassium decline during hospitalization for acute decompensated heart failure is a predictor of 6-month mortality, independent of N-terminal pro-B-type natriuretic peptide levels: An individual patient data analysis. Am Heart J 2015.
1021. Salah S, Watanabe K, Welter S, et al. Colorectal cancer pulmonary oligometastases: pooled analysis and construction of a clinical lung metastasectomy prognostic model. Ann Oncol 2012;**23**(10):2649-55.
1022. Salameh H, Raff E, Erwin A, et al. PNPLA3 gene polymorphism is associated with predisposition to and severity of alcoholic liver disease. Am J Gastroenterol 2015;**110**(6):846-56.
1023. Salerno F, Camma C, Enea M, et al. Transjugular Intrahepatic Portosystemic Shunt for Refractory Ascites: A Meta-analysis of Individual Patient Data. Gastroenterology 2007;**133**:825-34.
1024. Samara MT, Leucht C, Leeflang MM, et al. Early Improvement As a Predictor of Later Response to Antipsychotics in Schizophrenia: A Diagnostic Test Review. Am J Psychiatry 2015;**172**(7):617-29.
1025. Sandret L, Maison P, Chanson P. Place of cabergoline in acromegaly: a meta-analysis. J Clin Endocrinol Metab 2011;**96**(5):1327-35.
1026. Sarcoma Meta-analysis Collaboration S. Adjuvant chemotherapy for localised resectable soft tissue sarcoma in adults.[update of Cochrane Database Syst Rev. 2000;(2):CD001419; PMID: 10796873]. [Review] [45 refs]. Cochrane Database Syst Rev 2000(4):CD001419.
1027. Sargent D, Shi Q, Yothers G, et al. Two or three year disease-free survival (DFS) as a primary end-point in stage III adjuvant colon cancer trials with fluoropyrimidines with or without oxaliplatin or irinotecan: data from 12,676 patients from MOSAIC, X-ACT, PETACC-3, C-06, C-07 and C89803. Eur J Cancer 2011;**47**(7):990-6.

1028. Sargent DJ, Goldberg RM, Jacobson SD, et al. A pooled analysis of adjuvant chemotherapy for resected colon cancer in elderly patients.[see comment]. N Engl J Med 2001;**345**(15):1091-7.
1029. Sargent DJ, Kohne CH, Sanoff HK, et al. Pooled safety and efficacy analysis examining the effect of performance status on outcomes in nine first-line treatment trials using individual data from patients with metastatic colorectal cancer. J Clin Oncol 2009;**27**(12):1948-55.
1030. Sargent DJ, Patiyil S, Yothers G, et al. End points for colon cancer adjuvant trials: Observations and recommendations based on individual patient data from 20,898 patients enrolled onto 18 randomized trials from the ACCENT group. J Clin Oncol 2007;**25**(29):4569-74.
1031. Sargent DJ, Wieand HS, Haller DG, et al. Disease-free survival versus overall survival as a primary end point for adjuvant colon cancer studies: Individual patient data from 20,898 patients on 18 randomized trials. J Clin Oncol 2005;**23**(34):8664-70.
1032. Sarwar N, Gao P, Seshasai SR, et al. Diabetes mellitus, fasting blood glucose concentration, and risk of vascular disease: a collaborative meta-analysis of 102 prospective studies. Lancet 2010;**375**(9733):2215-22.
1033. Satoh M, Ohkubo T, Asayama K, et al. Combined effect of blood pressure and total cholesterol levels on long-term risks of subtypes of cardiovascular death: Evidence for Cardiovascular Prevention from Observational Cohorts in Japan. Hypertension 2015;**65**(3):517-24.
1034. Schacht A, Gorwood P, Boyce P, et al. Depression symptom clusters and their predictive value for treatment outcomes: Results from an individual patient data meta-analysis of duloxetine trials. J Psychiatr Res 2014;**53**(1):54-61.
1035. Schaich M, Schlenk RF, Al-Ali HK, et al. Prognosis of acute myeloid leukemia patients up to 60 years of age exhibiting trisomy 8 within a non-complex karyotype: Individual patient data-based meta-analysis of the German Acute Myeloid Leukemia Intergroup. Haematologica 2007;**92**(6):763-70.
1036. Schalm SW, Hansen BE, Chemello L, et al. Ribavirin enhances the efficacy but not the adverse effects of interferon in chronic hepatitis C. Meta-analysis of individual patient data from European centers. J Hepatol 1997;**26**(5):961-6.
1037. Schalm SW, Weiland O, Hansen BE, et al. Interferon-ribavirin for chronic hepatitis C with and without cirrhosis: analysis of individual patient data of six controlled trials. Eurohep Study Group for Viral Hepatitis. Gastroenterology 1999;**117**(2):408-13.
1038. Schernthaner G, Barnett AH, Patel S, et al. Safety and efficacy of the dipeptidyl peptidase-4 inhibitor linagliptin in elderly patients with type 2 diabetes: a comprehensive analysis of data from 1331 individuals aged > 65 years. Diabetes, Obesity and Metabolism 2014;**16**(11):1078-86.
1039. Schinkel J, Spoon WJ, Kroes AC. Meta-analysis of mutations in the NS5A gene and hepatitis C virus resistance to interferon therapy: uniting discordant conclusions. Antivir Ther 2004;**9**(2):275-86.
1040. Schlamann A, Von Bueren AO, Hagel C, et al. An individual patient data meta-analysis on characteristics and outcome of patients with papillary glioneuronal tumor, rosette glioneuronal tumor with neuropil-like islands and rosette forming glioneuronal tumor of the fourth ventricle. PLoS One 2014;**9**(7).
1041. Schlenk RF, Benner A, Krauter J, et al. Individual patient data-based meta-analysis of patients aged 16 to 60 years with core binding factor acute myeloid leukemia: a survey of the German Acute Myeloid Leukemia Intergroup. J Clin Oncol 2004;**22**(18):3741-50.
1042. Schmoll H-J, Twelves C, Sun W, et al. Effect of adjuvant capecitabine or fluorouracil, with or without oxaliplatin, on survival outcomes in stage III colon cancer and the effect of oxaliplatin on post-relapse survival: a pooled analysis of individual patient data from four randomised controlled trials. Lancet Oncol 2014;**15**(13):1481-92.
1043. Schottker B, Jorde R, Peasey A, et al. Vitamin D and mortality: meta-analysis of individual participant data from a large consortium of cohort studies from Europe and the United States. BMJ 2014;**348**:g3656.
1044. Schuetz P, Muller B, Christ-Crain M, et al. Procalcitonin to initiate or discontinue antibiotics in acute respiratory tract infections. Cochrane Database Syst Rev 2012;**9**:CD007498.
1045. Schuit E, Amer-Wahlin I, Ojala K, et al. Effectiveness of electronic fetal monitoring with additional ST analysis in vertex singleton pregnancies at >36 weeks of gestation: an individual participant data metaanalysis. Am J Obstet Gynecol 2013;**208**(3):187.e1-87.e13.
1046. Schuit E, Stock S, Rode L, et al. Effectiveness of progestogens to improve perinatal outcome in twin pregnancies: an individual participant data meta-analysis. BJOG 2015;**122**(1):27-37.
1047. Scott JR. Immunotherapy for recurrent miscarriage.[update of Cochrane Database Syst Rev. 2000;(2):CD000112; PMID: 10796135]. [Review] [54 refs]. Cochrane Database Syst Rev 2003(1):CD000112.
1048. Scott NW, McCormack K, Graham P, et al. Open mesh versus non-mesh for repair of femoral and inguinal hernia. Cochrane Database Syst Rev 2002(4):CD002197.

1049. Sebba AI, Emkey RD, Kohles JD, et al. Ibandronate dose response is associated with increases in bone mineral density and reductions in clinical fractures: Results of a meta-analysis. Bone 2009;**44**(3):423-27.
1050. Serpa Neto A, Hemmes SNT, Barbas CSV, et al. Protective versus Conventional Ventilation for Surgery: A Systematic Review and Individual Patient Data Meta-analysis. Anesthesiology 2015;**123**(1):66-78.
1051. Serpa Neto A, Hemmes SNT, Barbas CSV, et al. Incidence of mortality and morbidity related to postoperative lung injury in patients who have undergone abdominal or thoracic surgery: a systematic review and meta-analysis. Lancet Respir Med 2014;**2**(12):1007-15.
1052. Serpa Neto A, Simonis FD, Barbas CSV, et al. Association between tidal volume size, duration of ventilation, and sedation needs in patients without acute respiratory distress syndrome: an individual patient data meta-analysis. Intensive Care Med 2014;**40**(7):950-7.
1053. Serretti A, Cusin C, Rausch JL, et al. Pooling pharmacogenetic studies on the serotonin transporter: A mega-analysis. Psychiatry Res 2006;**145**(1):61-65.
1054. Seshasai SRK, Bennett RL, Petrie JR, et al. Cardiovascular safety of the glucagon-like peptide-1 receptor agonist taspoglutide in people with type 2 diabetes: An individual participant data meta-analysis of randomized controlled trials. Diabetes, Obesity and Metabolism 2015;**17**(5):505-10.
1055. Seth A, Serruys PW, Lansky A, et al. A pooled gender based analysis comparing the XIENCE V(R) everolimus-eluting stent and the TAXUS paclitaxel-eluting stent in male and female patients with coronary artery disease, results of the SPIRIT II and SPIRIT III studies: two-year analysis. EuroIntervention 2010;**5**(7):788-94.
1056. Shaikh N, Craig JC, Rovers MM, et al. Identification of children and adolescents at risk for renal scarring after a first urinary tract infection: a meta-analysis with individual patient data. "Jama, Pediatr" 2014;**168**(10):893-900.
1057. Sharma SK, McIntire DD, Wiley J, et al. Labor analgesia and cesarean delivery: an individual patient meta-analysis of nulliparous women. Anesthesiology 2004;**100**(1):142-8; discussion 6A.
1058. Shaw GL, Wilson P, Cuzick J, et al. International study into the use of intermittent hormone therapy in the treatment of carcinoma of the prostate: a meta-analysis of 1446 patients. BJU Int 2007;**99**(5):1056-65.
1059. Shekelle PG, Rich MW, Morton SC, et al. Efficacy of angiotensin-converting enzyme inhibitors and beta-blockers in the management of left ventricular systolic dysfunction according to race, gender, and diabetic status: a meta-analysis of major clinical trials. J Am Coll Cardiol 2003;**41**(9):1529-38.
1060. Shepperd S, Doll H, Angus Robert M, et al. Admission avoidance hospital at home. Cochrane Database Syst Rev 2008(4).
1061. Shepperd S, Doll H, Broad J, et al. Early discharge hospital at home. Cochrane Database Syst Rev 2009(1).
1062. Sherif A, Holmberg L, Rintala E, et al. Neoadjuvant cisplatinum based combination chemotherapy in patients with invasive bladder cancer: a combined analysis of two Nordic studies. Eur Urol 2004;**45**(3):297-303.
1063. Sherwood M, Thornton AE, Honer WG. A quantitative review of the profile and time course of symptom change in schizophrenia treated with clozapine. J Psychopharmacol 2012;**26**(9):1175-84.
1064. Shi Q, de Gramont A, Grothey A, et al. Individual patient data analysis of progression-free survival versus overall survival as a first-line end point for metastatic colorectal cancer in modern randomized trials: findings from the analysis and research in cancers of the digestive system database. J Clin Oncol 2015;**33**(1):22-8.
1065. Sieber J, Koberlein J, Mosges R. Sublingual immunotherapy in daily medical practice: effectiveness of different treatment schedules - IPD meta-analysis. Curr Med Res Opin 2010;**26**(4):925-32.
1066. Sieber J, Shah-Hosseini K, Mosges R. Specific immunotherapy for allergic rhinitis to grass and tree pollens in daily medical practice-symptom load with sublingual immunotherapy compared to subcutaneous immunotherapy. Ann Med 2011;**43**(6):418-24.
1067. Siegrist J, Dragano N, Nyberg ST, et al. Validating abbreviated measures of effort-reward imbalance at work in European cohort studies: The IPD-Work consortium. Int Arch Occup Environ Health 2014;**87**(3):249-56.
1068. Signorovitch J, Ramakrishnan K, Ben-Hamadi R, et al. Remission of major depressive disorder without adverse events: a comparison of escitalopram versus serotonin norepinephrine reuptake inhibitors.[Erratum appears in Curr Med Res Opin. 2011 Sep;27(9):1708]. Curr Med Res Opin 2011;**27**(6):1089-96.
1069. Silva IS, De SB, McCormack V, et al. Birth size and breast cancer risk: re-analysis of individual participant data from 32 studies.[see comment]. PLoS Med 2008;**5**(9):e193-.
1070. Simes J, Becattini C, Agnelli G, et al. Aspirin for the prevention of recurrent venous thromboembolism: the INSPIRE collaboration. Circulation 2014;**130**(13):1062-71.
1071. Simmonds MC, Brown JVE, Heirs MK, et al. Safety and effectiveness of recombinant human bone morphogenetic protein-2 for spinal fusion: a meta-analysis of individual-participant data. Ann Intern Med 2013;**158**(12):877-89.

1072. Simmonds PC. Palliative chemotherapy for advanced colorectal cancer: systematic review and meta-analysis. Colorectal Cancer Collaborative Group. BMJ 2000;**321**(7260):531-5.
1073. Simon GR, Schell MJ, Begum M, et al. Preliminary indication of survival benefit from ERCC1 and RRM1-tailored chemotherapy in patients with advanced nonsmall cell lung cancer: evidence from an individual patient analysis. Cancer 2012;**118**(9):2525-31.
1074. Sin DD, Tashkin D, Zhang X, et al. Budesonide and the risk of pneumonia: a meta-analysis of individual patient data. Lancet 2009;**374**(9691):712-9.
1075. Sin DD, Wu L, Anderson JA, et al. Inhaled corticosteroids and mortality in chronic obstructive pulmonary disease. Thorax 2005;**60**(12):992-97.
1076. Singh JA, Sloan JA, Atherton PJ, et al. Preferred roles in treatment decision making among patients with cancer: a pooled analysis of studies using the Control Preferences Scale. Am J Manag Care 2010;**16**(9):688-96.
1077. Singh S, Venkatesh SK, Wang Z, et al. Diagnostic performance of magnetic resonance elastography in staging liver fibrosis: A systematic review and meta-analysis of individual participant data. Clin Gastroenterol Hepatol 2015;**13**(3):440-51.
1078. Sipahi I, Tuzcu EM, Wolski KE, et al. beta-blockers and progression of coronary atherosclerosis: Pooled analysis of 4 intravascular ultrasonography trials. Ann Intern Med 2007;**147**(1):10-18.
1079. Skoetz N, Trelle S, Rancea M, et al. Effect of initial treatment strategy on survival of patients with advanced-stage Hodgkin's lymphoma: a systematic review and network meta-analysis. Lancet Oncol 2013;**14**(10):943-52.
1080. Smart NA, Meyer T, Butterfield JA, et al. Individual patient meta-analysis of exercise training effects on systemic brain natriuretic peptide expression in heart failure. European Journal of Preventive Cardiology 2012;**19**(3):428-35.
1081. Smith CT, Marson AG, Chadwick DW, et al. Multiple treatment comparisons in epilepsy monotherapy trials. Trials 2007;**8**(34):-.
1082. Smith-Warner SA, Spiegelman D, Yaun S-S, et al. Alcohol and breast cancer in women: a pooled analysis of cohort studies. JAMA 1998;**279**(7):535-40.
1083. Sofi AA, Silverman AL, Khuder S, et al. Relationship of symptom duration and fecal bacteriotherapy in Clostridium difficile infection-pooled data analysis and a systematic review. Scand J Gastroenterol 2013;**48**(3):266-73.
1084. Solomon SD, Pfeffer MA, McMurray JJV, et al. Effect of celecoxib on cardiovascular events and blood pressure in two trials for the prevention of colorectal adenomas. Circulation 2006;**114**(10):1028-35.
1085. Solomon SD, Wittes J, Finn PV, et al. Cardiovascular risk of celecoxib in 6 randomized placebo-controlled trials: the cross trial safety analysis. Circulation 2008;**117**(16):2104-13.
1086. Song GY, Hong L, Zhang H, et al. Iatrogenic medial patellar instability following lateral retinacular release of the knee joint. Knee Surgery, Sports Traumatology, Arthroscopy 2015.
1087. Sonnenschein-Van Der Voort AMM, Arends LR, De Jongste JC, et al. Preterm birth, infant weight gain, and childhood asthma risk: A meta-analysis of 147,000 European children. J Allergy Clin Immunol 2014;**133**(5):1317-29.
1088. Soriano JB, Lamprecht B, Ramírez AS, et al. Mortality prediction in chronic obstructive pulmonary disease comparing the GOLD 2007 and 2011 staging systems: A pooled analysis of individual patient data. Lancet Respir Med 2015;**3**(6):443-50.
1089. Soriano JB, Sin DD, Zhang X, et al. A pooled analysis of FEV1 decline in COPD patients randomized to inhaled corticosteroids or placebo. Chest 2007;**131**(3):682-9.
1090. Sormani MP, Stubinski B, Cornelisse P, et al. Magnetic resonance active lesions as individual-level surrogate for relapses in multiple sclerosis. Mult Scler 2011;**17**(5):541-9.
1091. Souverein OW, de Vries JHM, Freese R, et al. Prediction of fruit and vegetable intake from biomarkers using individual participant data of diet-controlled intervention studies. Br J Nutr 2015;**113**(9):1396-409.
1092. Spaulding C, Daemen J, Boersma E, et al. A pooled analysis of data comparing sirolimus-eluting stents with bare-metal stents. N Engl J Med 2007;**356**(10):989-97.
1093. Specht L, Gray R, Clarke M, et al. Influence of more extensive radiotherapy and adjuvant chemotherapy on long-term outcome of early-stage Hodgkin's disease: a meta-analysis of 23 randomized trials involving 3,888 patients. International Hodgkin's Disease Collaborative Group. J Clin Oncol 1998;**16**(3):830-43.
1094. Stahl SM, Entsuah R, Rudolph RL. Comparative efficacy between venlafaxine and SSRIs: a pooled analysis of patients with depression. Biol Psychiatry 2002;**52**(12):1166-74.
1095. Staples MP, Kallmes DF, Comstock BA, et al. Effectiveness of vertebroplasty using individual patient data from two randomised placebo controlled trials: meta-analysis. BMJ 2011;**343**:d3952.

1096. Staszewski S, DeMasi R, Hill AM, et al. HIV-1 RNA, CD4 cell count and the risk of progression to AIDS and death during treatment with HIV-1 reverse transcriptase inhibitors. AIDS 1998;**12**(15):1991-97.
1097. Steele KM, Damiano DL, Eek MN, et al. Characteristics associated with improved knee extension after strength training for individuals with cerebral palsy and crouch gait. J Pediatr Rehabil Med 2012;**5**(2):99-106.
1098. Stefanini GG, Kalesan B, Pilgrim T, et al. Impact of sex on clinical and angiographic outcomes among patients undergoing revascularization with drug-eluting stents. JACC Cardiovasc Interv 2012;**5**(3):301-10.
1099. Steiner TJ, Voelker M. Gastrointestinal tolerability of aspirin and the choice of over-the-counter analgesia for short-lasting acute pain. J Clin Pharm Ther 2009;**34**(2):177-86.
1100. Stempel DA, Stanford RH, Thwaites RM, et al. Cost-efficacy comparison of inhaled fluticasone propionate and budesonide in the treatment of asthma. Clin Ther 2000;**22**(12):1562-74.
1101. Stengel D, Bauwens K, Rademacher G, et al. Emergency ultrasound-based algorithms for diagnosing blunt abdominal trauma. Cochrane Database Syst Rev 2013;**7**:CD004446.
1102. Stewart LA. Chemotherapy in adult high-grade glioma: a systematic review and meta-analysis of individual patient data from 12 randomised trials. [Review] [36 refs]. Lancet 2002;**359**(9311):1011-8.
1103. Steyerberg EW, Mushkudiani N, Perel P, et al. Predicting outcome after traumatic brain injury: Development and international validation of prognostic scores based on admission characteristics. PLoS Med 2008;**5**(8):1251-61.
1104. Stocken DD, Buchler MW, Dervenis C, et al. Meta-analysis of randomised adjuvant therapy trials for pancreatic cancer. Br J Cancer 2005;**92**(8):1372-81.
1105. Stojkovic M, Zwahlen M, Teggi A, et al. Treatment response of cystic echinococcosis to benzimidazoles: a systematic review. PLoS Negl Trop Dis 2009;**3**(9):e524.
1106. Stone GW, Ellis SG, Colombo A, et al. Offsetting impact of thrombosis and restenosis on the occurrence of death and myocardial infarction after paclitaxel-eluting and bare metal stent implantation. Circulation 2007;**115**(22):2842-7.
1107. Storosum JG, Elferink AJ, van Zwieten BJ, et al. Short-term efficacy of tricyclic antidepressants revisited: a meta-analytic study. Eur Neuropsychopharmacol 2001;**11**(2):173-80.
1108. Strand V, Conaghan PG, Lohmander LS, et al. An integrated analysis of five double-blind, randomized controlled trials evaluating the safety and efficacy of a hyaluronan product for intra-articular injection in osteoarthritis of the knee. Osteoarthritis Cartilage 2006;**14**(9):859-66.
1109. Straube S, Moore RA, Paine J, et al. Interference with work in fibromyalgia: effect of treatment with pregabalin and relation to pain response. BMC Musculoskelet Disord 2011;**12**:125.
1110. Struik FM, Lacasse Y, Goldstein RS, et al. Nocturnal noninvasive positive pressure ventilation in stable COPD: A systematic review and individual patient data meta-analysis. Respir Med 2014;**108**(2):329-37.
1111. Sun JC, Xu T, Chen KF, et al. Assessment of cauda equina syndrome progression pattern to improve diagnosis. Spine 2014;**39**(7):596-602.
1112. Sun X, Xu D, Luo F, et al. A cross-cultural perspective on the preference for potential effect: An individual participant data (IPD) meta-analysis approach. PLoS One 2015;**10**(3).
1113. Sundstrom J, Arima H, Jackson R, et al. Effects of blood pressure reduction in mild hypertension: a systematic review and meta-analysis. Ann Intern Med 2015;**162**(3):184-91.
1114. Sweeting MJ, Thompson SG, Brown LC, et al. Meta-analysis of individual patient data to examine factors affecting growth and rupture of small abdominal aortic aneurysms. Br J Surg 2012;**99**(5):655-65.
1115. Sweeting MJ, Ulug P, Powell JT, et al. Ruptured Aneurysm Trials: The Importance of Longer-term Outcomes and Meta-analysis for 1-year Mortality. Eur J Vasc Endovasc Surg 2015.
1116. Sylaja PN, Dong W, Grotta JC, et al. Safety outcomes of Alteplase among acute ischemic stroke patients with special characteristics. Neurocrit Care 2007;**6**(3):181-85.
1117. Sylvester RJ, Oosterlinck W, Holmang S, et al. Systematic Review and Individual Patient Data Meta-analysis of Randomized Trials Comparing a Single Immediate Instillation of Chemotherapy After Transurethral Resection with Transurethral Resection Alone in Patients with Stage pTa-pT1 Urothelial Carcinoma of the Bladder: Which Patients Benefit from the Instillation? Eur Urol 2015.
1118. Sylvester RJ, Van Der Meijden APM, Oosterlinck W, et al. Predicting recurrence and progression in individual patients with stage Ta T1 bladder cancer using EORTC risk tables: A combined analysis of 2596 patients from seven EORTC trials. Eur Urol 2006;**49**(3):466-75.
1119. Szczech LA, Berlin JA, Feldman HI. The effect of antilymphocyte induction therapy on renal allograft survival. A meta-analysis of individual patient-level data. Anti-Lymphocyte Antibody Induction Therapy Study Group.[see comment]. Ann Intern Med 1998;**128**(10):817-26.
1120. Takenaka Y, Cho H, Nakahara S, et al. Chemoradiation therapy for squamous cell carcinoma of the external auditory canal: A meta-analysis. Head Neck 2015;**37**(7):1073-80.

1121. Tandon P, Abraldes JG, Berzigotti A, et al. Renin-angiotensin-aldosterone inhibitors in the reduction of portal pressure: a systematic review and meta-analysis. J Hepatol 2010;**53**(2):273-82.
1122. Tanvetyanon T, Finley DJ, Fabian T, et al. Prognostic factors for survival after complete resections of synchronous lung cancers in multiple lobes: pooled analysis based on individual patient data. Ann Oncol 2013;**24**(4):889-94.
1123. Taylor S, Tudur Smith C, Williamson PR, et al. Phenobarbitone versus phenytoin monotherapy for partial onset seizures and generalized onset tonic-clonic seizures. [Review] [16 refs]. Cochrane Database Syst Rev 2001(4):CD002217.
1124. Terasawa T, Nihashi T, Hotta T, et al. F-FDG PET for posttherapy assessment of Hodgkin's disease and aggressive non-Hodgkin's lymphoma: A systematic review. J Nucl Med 2008;**48**(1):13-21.
1125. Terwee CB, Nieveen Van Dijkum EJ, Gouma DJ, et al. Pooling of prognostic studies in cancer of the pancreatic head and periampullary region: the Triple-P study. Triple-P study group. Eur J Surg 2000;**166**(9):706-12.
1126. Teulings H-E, Limpens J, Jansen SN, et al. Vitiligo-like depigmentation in patients with stage III-IV melanoma receiving immunotherapy and its association with survival: a systematic review and meta-analysis. J Clin Oncol 2015;**33**(7):773-81.
1127. Teunis T, Lubberts B, Reilly BT, et al. A systematic review and pooled analysis of the prevalence of rotator cuff disease with increasing age. J Shoulder Elbow Surg 2014;**23**(12):1913-21.
1128. Thakkinstian A, Dmitrienko S, Gerbase-DeLima M, et al. Association between cytokine gene polymorphisms and outcomes in renal transplantation: A meta-analysis of individual patient data. Nephrology Dialysis Transplantation 2008;**29**(9):3017-23.
1129. Thakkinstian A, Nickel JC. Efficacy of intravesical chondroitin sulphate in treatment of interstitial cystitis/bladder pain syndrome (IC/BPS): Individual patient data (IPD) meta-analytical approach. Journal of the Canadian Urological Association 2013;**7**(5-6 JUN):195-200.
1130. Thankappan K. Basaloid squamous cell carcinoma of the larynx--A systematic review. Auris, Nasus, Larynx 2012;**39**(4):397-401.
1131. Thase ME. Effects of venlafaxine on blood pressure: a meta-analysis of original data from 3744 depressed patients. J Clin Psychiatry 1998;**59**(10):502-08.
1132. Thase ME, Demyttenaere K, Earley WR, et al. Extended release quetiapine fumarate in major depressive disorder: analysis in patients with anxious depression. Depress Anxiety 2012;**29**(7):574-86.
1133. Thase ME, Haight BR, Richard N, et al. Remission rates following antidepressant therapy with bupropion or selective serotonin reuptake inhibitors: A meta-analysis of original data from 7 randomized controlled trials. J Clin Psychiatry 2005;**66**(8):974-81.
1134. Thase ME, Kornstein SG, Germain J-M, et al. An integrated analysis of the efficacy of desvenlafaxine compared with placebo in patients with major depressive disorder. Cns Spectrums 2009;**14**(3):144-54.
1135. Thase ME, Nierenberg AA, Vrijland P, et al. Remission with mirtazapine and selective serotonin reuptake inhibitors: a meta-analysis of individual patient data from 15 controlled trials of acute phase treatment of major depression. Int Clin Psychopharmacol 2010;**25**(4):189-98.
1136. Thase ME, Pritchett YL, Ossanna MJ, et al. Efficacy of duloxetine and selective serotonin reuptake inhibitors: Comparisons as assessed by remission rates in patients with major depressive disorder. J Clin Psychopharmacol 2007;**27**(6):672-76.
1137. The International Study Group on Improved ORS. Impact of glycine-containing ORS solutions on stool output and duration of diarrhoea: a meta-analysis of seven clinical trials. . Bull World Health Organ 1991;**69**(5):541-8.
1138. The SYROCOT study group. Effectiveness of prenatal treatment for congenital toxoplasmosis: a meta-analysis of individual patients' data. Lancet 2007;**369**(9556):115-22.
1139. Thirion P, Piedbois P, Buyse M, et al. Alpha-interferon does not increase the efficacy of 5-fluorouracil in advanced colorectal cancer. Br J Cancer 2001;**84**(5):611-20.
1140. Thirion P, Wolmark N, Haddad E, et al. Survival impact of chemotherapy in patients with colorectal metastases confined to the liver: a re-analysis of 1458 non-operable patients randomised in 22 trials and 4 meta-analyses. Meta-Analysis Group in Cancer. Ann Oncol 1999;**10**(11):1317-20.
1141. Thompson A, Gao P, Orfei L, et al. Lipoprotein-associated phospholipase A(2) and risk of coronary disease, stroke, and mortality: collaborative analysis of 32 prospective studies. Lancet 2010;**375**(9725):1536-44.
1142. Thompson DD, Murray GD, Candelise L, et al. Targeting aspirin in acute disabling ischemic stroke: An individual patient data meta-analysis of three large randomized trials. Int J Stroke 2015.
1143. Thorgrimsen L, Spector A, Wiles A, et al. Aroma therapy for dementia. [Review] [46 refs]. Cochrane Database Syst Rev 2003(3):CD003150.

1144. Thrift AP, Cook MB, Vaughan TL, et al. Alcohol and the risk of Barrett's esophagus: a pooled analysis from the International BEACON Consortium. Am J Gastroenterol 2014;**109**(10):1586-94.
1145. Tierney J, Benedetti-Panici P, Bermudez A, et al. Neoadjuvant chemotherapy for locally advanced cervix cancer. Cochrane Database Syst Rev 2009(4).
1146. Timmer JR, Ottervanger JP, De BM-J, et al. Primary percutaneous coronary intervention compared with fibrinolysis for myocardial infarction in diabetes mellitus: Results from the primary coronary angioplasty vs thrombolysis-2 trial. Arch Intern Med 2007;**167**(13):1353-59.
1147. Timmermans A, Opmeer BC, Khan KS, et al. Endometrial thickness measurement for detecting endometrial cancer in women with postmenopausal bleeding: a systematic review and meta-analysis. Obstet Gynecol 2010;**116**(1):160-7.
1148. Tonkin AM. Clinical relevance of statins: their role in secondary prevention. [Review] [10 refs]. Atherosclerosis Supplements 2001;**2**(1):21-5.
1149. Tornvall P, Gerbaud E, Behaghel A, et al. Myocarditis or "true" infarction by cardiac magnetic resonance in patients with a clinical diagnosis of myocardial infarction without obstructive coronary disease: A meta-analysis of individual patient data. Atherosclerosis 2015;**241**(1):87-91.
1150. Touze E, Varenne O, Chatellier G, et al. Risk of myocardial infarction and vascular death after transient ischemic attack and ischemic stroke: A systematic review and meta-analysis. Stroke 2005;**36**(12):2748-55.
1151. Townsend A, Price T, Karapetis C. Selective internal radiation therapy for liver metastases from colorectal cancer. Cochrane Database Syst Rev 2009(4):CD007045.
1152. Trépo E, Nahon P, Bontempi G, et al. Association between the PNPLA3 (rs738409 C>G) variant and hepatocellular carcinoma: Evidence from a meta-analysis of individual participant data. Hepatology 2014.
1153. Trialists' CollaborativeGroup C. Chemotherapeutic options in chronic lymphocytic leukemia: a meta-analysis of the randomized trials. J Natl Cancer Inst 1999;**91**(10):861-68.
1154. Trialists'Collaboration SU. How do stroke units improve patient outcomes? A collaborative systematic review of the randomized trials. Stroke 1997;**28**(11):2139-44.
1155. Trivella M, Pezzella F, Pastorino U, et al. Microvessel density as a prognostic factor in non-small-cell lung carcinoma: a meta-analysis of individual patient data. Lancet Oncol 2007;**8**(6):488-99.
1156. Troughton RW, Frampton CM, Brunner-La Rocca H-P, et al. Effect of B-type natriuretic peptide-guided treatment of chronic heart failure on total mortality and hospitalization: an individual patient meta-analysis. Eur Heart J 2014;**35**(23):1559-67.
1157. Trumper M, Ross PJ, Cunningham D, et al. Efficacy and tolerability of chemotherapy in elderly patients with advanced oesophago-gastric cancer: A pooled analysis of three clinical trials. Eur J Cancer 2006;**42**(7):827-34.
1158. Tsang AC, Ahmadi Pirshahid S, Virgili G, et al. Hydroxychloroquine and chloroquine retinopathy: a systematic review evaluating the multifocal electroretinogram as a screening test. Ophthalmology 2015;**122**(6):1239-51.e4.
1159. Tudur Smith C, Marson AG, Clough HE, et al. Carbamazepine versus phenytoin monotherapy for epilepsy. [Review] [47 refs]. Cochrane Database Syst Rev 2002(2):CD001911.
1160. Tudur Smith C, Marson AG, Williamson PR. Phenytoin versus valproate monotherapy for partial onset seizures and generalized onset tonic-clonic seizures. [Review] [40 refs]. Cochrane Database Syst Rev 2001(4):CD001769.
1161. Tudur Smith C, Marson AG, Williamson PR. Carbamazepine versus phenobarbitone monotherapy for epilepsy. [Review] [21 refs]. Cochrane Database Syst Rev 2003(1):CD001904.
1162. Tyson SF, Crow JL, Connell L, et al. Sensory impairments of the lower limb after stroke: a pooled analysis of individual patient data. Top Stroke Rehabil 2013;**20**(5):441-9.
1163. Ueberall MA, Mueller-Schwefe GHH, Terhaag B. Efficacy and tolerability of flupirtine in subacute/ chronic musculoskeletal pain - results of a patient level, pooled re-analysis of randomized, double-blind, controlled trials. Int J Clin Pharmacol Ther 2011;**49**(11):637-47.
1164. Unverzagt S, Machemer M, Solms A, et al. Intra-aortic balloon pump counterpulsation (IABP) for myocardial infarction complicated by cardiogenic shock. Cochrane Database Syst Rev 2015;**3**.
1165. Unverzagt S, Machemer M-T, Solms A, et al. Intra-aortic balloon pump counterpulsation (IABP) for myocardial infarction complicated by cardiogenic shock. Cochrane Database Syst Rev 2011(7):CD007398.
1166. Uronis HE, Currow DC, McCrory DC, et al. Oxygen for relief of dyspnoea in mildly- or non-hypoxaemic patients with cancer: A systematic review and meta-analysis. Br J Cancer 2008;**98**(2):294-99.
1167. Vale C, Tierney JF, Stewart LA, et al. Reducing uncertainties about the effects of chemoradiotherapy for cervical cancer: A systematic review and meta-analysis of individual patient data from 18 randomized trials. J Clin Oncol 2008;**26**(35):5802-12.

1168. Vale CL. Neoadjuvant chemotherapy in invasive bladder cancer: Update of a systematic review and meta-analysis of individual patient data. Eur Urol 2005;**48**(2):202-05.
1169. Valery PC, Williams G, Sleigh AC, et al. Parental occupation and Ewing's sarcoma: pooled and meta-analysis. Int J Cancer 2005;**115**(5):799-806.
1170. Valgimigli M, Sabate M, Kaiser C, et al. Effects of cobalt-chromium everolimus eluting stents or bare metal stent on fatal and non-fatal cardiovascular events: patient level meta-analysis. BMJ 2014;**349**:g6427.
1171. Valle JW, Furuse J, Jitlal M, et al. Cisplatin and gemcitabine for advanced biliary tract cancer: A meta-analysis of two randomised trials. Ann Oncol 2014;**25**(2):391-98.
1172. Van Beek JGM, Mushkudiani NA, Steyerberg EW, et al. Prognostic value of admission laboratory parameters in traumatic brain injury: Results from the IMPACT study. J Neurotrauma 2007;**24**(2):315-28.
1173. van de Beek D, Farrar JJ, de Gans J, et al. Adjunctive dexamethasone in bacterial meningitis: a meta-analysis of individual patient data. Lancet Neurol 2010;**9**(3):254-63.
1174. van der A DL, Rovers MM, Grobbee DE, et al. Mutations in the HFE gene and cardiovascular disease risk: an individual patient data meta-analysis of 53 880 subjects. Circulation Cardiovascular Genetics 2008;**1**(1):43-50.
1175. van der Pas MH, Meijer S, Hoekstra OS, et al. Sentinel-lymph-node procedure in colon and rectal cancer: a systematic review and meta-analysis. Lancet Oncol 2011;**12**(6):540-50.
1176. van der Velde M, Matsushita K, Coresh J, et al. Lower estimated glomerular filtration rate and higher albuminuria are associated with all-cause and cardiovascular mortality. A collaborative meta-analysis of high-risk population cohorts. Kidney Int 2011;**79**(12):1341-52.
1177. Van Grunsven P, Van Schayck C, Derenne J, et al. Long term effects of inhaled corticosteroids in chronic obstructive pulmonary disease: a meta-analysis. Thorax 1999;**54**(1):7-14.
1178. van Leeuwen N, Lingsma HF, Perel P, et al. Prognostic value of major extracranial injury in traumatic brain injury: an individual patient data meta-analysis in 39,274 patients. Neurosurgery 2012;**70**(4):811-8; discussion 18.
1179. Van Loo HM, Van Den Heuvel ER, Schoevers RA, et al. Sex dependent risk factors for mortality after myocardial infarction: Individual patient data meta-analysis. BMC Med 2014;**12**(1).
1180. van Oostwaard MF, Langenveld J, Schuit E, et al. Recurrence of hypertensive disorders of pregnancy: an individual patient data metaanalysis. Am J Obstet Gynecol 2015;**212**(5):624.e1-17.
1181. van Stel HF, Busschbach JJV, Hunink MGM, et al. Impact of secondary cardiovascular events on health status. Value Health 2012;**15**(1):175-82.
1182. van Walraven C, Hart RG, Singer DE, et al. Oral anticoagulants vs aspirin in nonvalvular atrial fibrillation: an individual patient meta-analysis. JAMA 2002;**288**(19):2441-8.
1183. Vansteenkiste J, Glaspy J, Henry D, et al. Benefits and risks of using erythropoiesis-stimulating agents (ESAs) in lung cancer patients: study-level and patient-level meta-analyses. Lung Cancer 2012;**76**(3):478-85.
1184. Vegter S, Tolley K, Wilson Waterworth T, et al. Meta-analysis using individual patient data: efficacy and durability of topical alicaforsen for the treatment of active ulcerative colitis. Aliment Pharmacol Ther 2013;**38**(3):284-93.
1185. Veldt BJ, Hansen BE, Eijkemans MJC, et al. Dynamic decision analysis to determine optimal treatment duration in chronic hepatitis C. Aliment Pharmacol Ther 2005;**21**(5):539-47.
1186. Veldt BJ, Hansen BE, Ikeda K, et al. Long-term clinical outcome and effect of glycyrrhizin in 1093 chronic hepatitis C patients with non-response or relapse to interferon. Scand J Gastroenterol 2006;**41**(9):1087-94.
1187. Veldt BJ, Saracco G, Boyer N, et al. Long term clinical outcome of chronic hepatitis C patients with sustained virological response to interferon monotherapy. [Review] [35 refs]. Gut 2004;**53**(10):1504-8.
1188. Velez JCQ, Nietert PJ. Therapeutic response to vasoconstrictors in hepatorenal syndrome parallels increase in mean arterial pressure: a pooled analysis of clinical trials. Am J Kidney Dis 2011;**58**(6):928-38.
1189. Venderbosch S, Nagtegaal ID, Maughan TS, et al. Mismatch repair status and BRAF mutation status in metastatic colorectal cancer patients: a pooled analysis of the CAIRO, CAIRO2, COIN, and FOCUS studies. Clin Cancer Res 2014;**20**(20):5322-30.
1190. Venkatesan M, Gadalla NB, Stepniewska K, et al. Polymorphisms in Plasmodium falciparum chloroquine resistance transporter and multidrug resistance 1 genes: parasite risk factors that affect treatment outcomes for P. falciparum malaria after artemether-lumefantrine and artesunate-amodiaquine.[Erratum appears in Am J Trop Med Hyg. 2015 May;92(5):1084; PMID: 25948561]. Am J Trop Med Hyg 2014;**91**(4):833-43.
1191. Verberg MFG, Eijkemans MJC, Macklon NS, et al. The clinical significance of the retrieval of a low number of oocytes following mild ovarian stimulation for IVF: a meta-analysis. Hum Reprod Update 2009;**15**(1):5-12.

1192. Verdecchia P, Reboldi GP, Angeli F, et al. Short- and long-term incidence of stroke in white-coat hypertension. Hypertension 2005;**45**(2):203-8.
1193. Verdich C, Flint A, Gutzwiller JP, et al. A meta-analysis of the effect of glucagon-like peptide-1 (7-36) amide on ad libitum energy intake in humans. J Clin Endocrinol Metab 2001;**86**(9):4382-9.
1194. Verdoux H, Geddes JR, Takei N, et al. Obstetric complications and age at onset in schizophrenia: an international collaborative meta-analysis of individual patient data.[see comment]. Am J Psychiatry 1997;**154**(9):1220-7.
1195. Vergouw CG, Heymans MW, Hardarson T, et al. No evidence that embryo selection by near-infrared spectroscopy in addition to morphology is able to improve live birth rates: results from an individual patient data meta-analysis. Hum Reprod 2014;**29**(3):455-61.
1196. Vergouw CG, Heymans MW, Hardarson T, et al. No evidence that embryo selection by near-infrared spectroscopy in addition to morphology is able to improve live birth rates: Results from an individual patient data meta-analysis. Hum Reprod 2014;**29**(3):455-61.
1197. Verheul R, Lehert P, Geerlings PJ, et al. Predictors of acamprosate efficacy: results from a pooled analysis of seven European trials including 1485 alcohol-dependent patients. Psychopharmacology (Berl) 2005;**178**(2-3):167-73.
1198. Verschure DO, Veltman CE, Manrique A, et al. For what endpoint does myocardial 123I-MIBG scintigraphy have the greatest prognostic value in patients with chronic heart failure? Results of a pooled individual patient data meta-analysis. Eur Heart J Cardiovasc Imaging 2014;**15**(9):996-1003.
1199. Vickers AJ, Cronin AM, Maschino AC, et al. Acupuncture for chronic pain: individual patient data meta-analysis. Arch Intern Med 2012;**172**(19):1444-53.
1200. Virtanen M, Jokela M, Nyberg ST, et al. Long working hours and alcohol use: systematic review and meta-analysis of published studies and unpublished individual participant data. BMJ 2015;**350**:g7772.
1201. Virtanen M, Nyberg ST, Batty GD, et al. Perceived job insecurity as a risk factor for incident coronary heart disease: systematic review and meta-analysis. BMJ 2013;**347**:f4746.
1202. von Bueren AO, Gerss J, Hagel C, et al. DNA copy number alterations in central primitive neuroectodermal tumors and tumors of the pineal region: an international individual patient data meta-analysis. J Neurooncol 2012;**109**(2):415-23.
1203. von Minckwitz G, Schwenkglenks M, Skacel T, et al. Febrile neutropenia and related complications in breast cancer patients receiving pegfilgrastim primary prophylaxis versus current practice neutropaenia management: results from an integrated analysis. Eur J Cancer 2009;**45**(4):608-17.
1204. von Minckwitz G, Untch M, Nuesch E, et al. Impact of treatment characteristics on response of different breast cancer phenotypes: pooled analysis of the German neo-adjuvant chemotherapy trials. Breast Cancer Res Treat 2011;**125**(1):145-56.
1205. Von Oppell UO, Dunne TT, De Groot MK, et al. Traumatic aortic rupture: twenty-year metaanalysis of mortality and risk of paraplegia. The Annals of Thoracic Surgery 1994;**58**(2):585-93.
1206. Vranckx P, Boersma E, Garg S, et al. Cardiovascular risk profile of patients included in stent trials; a pooled analysis of individual patient data from randomised clinical trials: insights from 33 prospective stent trials in Europe. EuroIntervention 2011;**7**(7):859-71.
1207. Wade C, Grady J, Kramer G. Efficacy of saline dextran (HSD) in patients with traumatic hypotension: meta-analysis of individual patient dataf hypertonic. Acta Anaesthesiol Scand Suppl 1997;**110**:77-9.
1208. Wade CE, Grady JJ, Kramer GC, et al. Individual patient cohort analysis of the efficacy of hypertonic saline/dextran in patients with traumatic brain injury and hypotension. [Review] [22 refs]. Journal of Trauma-Injury Infection and Critical Care 1997;**42**(5 Suppl):S61-5.
1209. Waldman AT, Stull LB, Galetta SL, et al. Pediatric optic neuritis and risk of multiple sclerosis: meta-analysis of observational studies. Journal of Aapos: American Association for Pediatric Ophthalmology and Strabismus 2011;**15**(5):441-6.
1210. Wali RK, Iyengar M, Beck GJ, et al. Efficacy and safety of carvedilol in treatment of heart failure with chronic kidney disease: a meta-analysis of randomized trials. Circ Heart Fail 2011;**4**(1):18-26.
1211. Walker MF, Leonardi-Bee J, Bath P, et al. Individual patient data meta-analysis of randomized controlled trials of community occupational therapy for stroke patients. Stroke 2004;**35**(9):2226-32.
1212. Walsh M, Mukhtyar C, Mahr A, et al. Health-related quality of life in patients with newly diagnosed antineutrophil cytoplasmic antibody-associated vasculitis. Arthritis Care Res 2011;**63**(7):1055-61.
1213. Walsh SR, Boyle JR, Lynch AG, et al. Suprarenal endograft fixation and medium-term renal function: Systematic review and meta-analysis. J Vasc Surg 2008;**47**(6):1364-70.
1214. Walti H, Paris-Llado J, Egberts J, et al. Prophylactic administration of porcine-derived lung surfactant is a significant factor in reducing the odds for peri-intraventricular haemorrhage in premature infants. Biol Neonate 2002;**81**(3):182-7.

1215. Wang D, Connock M, Barton P, et al. 'Cut down to quit' with nicotine replacement therapies in smoking cessation: A systematic review of effectiveness and economic analysis. Health Technol Assess 2008;**12**(2):i-156.
1216. Wang JG, Yan P, Jeffers BW. Effects of amlodipine and other classes of antihypertensive drugs on long-term blood pressure variability: Evidence from randomized controlled trials. J Am Soc Hypertens 2014;**8**(5):340-49.
1217. Wang J-G, Staessen JA, Franklin SS, et al. Systolic and diastolic blood pressure lowering as determinants of cardiovascular outcome. Hypertension 2005;**45**(5):907-13.
1218. Warde P, Specht L, Horwich A, et al. Prognostic factors for relapse in stage I seminoma managed by surveillance: a pooled analysis.[see comment]. J Clin Oncol 2002;**20**(22):4448-52.
1219. Wardlaw JM, Chappell FM, Stevenson M, et al. Accurate, practical and cost-effective assessment of carotid stenosis in the UK. Health Technol Assess 2006;**10**(30):iii-128.
1220. Warkentin TE, Sheppard J-A, Sigouin CS, et al. Gender imbalance and risk factor interactions in heparin-induced thrombocytopenia. Blood 2006;**108**(9):2937-41.
1221. Watts NB, Geusens P, Barton IP, et al. Relationship between changes in BMD and nonvertebral fracture incidence associated with risedronate: Reduction in risk of nonvertebral fracture is not related to change in BMD. J Bone Miner Res 2005;**20**(12):2097-104.
1222. Wedzicha JA, Dahl R, Buhl R, et al. Pooled safety analysis of the fixed-dose combination of indacaterol and glycopyrronium (QVA149), its monocomponents, and tiotropium versus placebo in COPD patients. Respir Med 2014;**108**(10):1498-507.
1223. Weinberg DV, Shapiro H, Ehrlich JS. Ranibizumab treatment outcomes in phakic versus pseudophakic eyes: an individual patient data analysis of 2 phase 3 trials. Ophthalmology 2013;**120**(6):1278-82.
1224. Weiner DE, Tighiouart H, Amin MG, et al. Chronic kidney disease as a risk factor for cardiovascular disease and all-cause mortality: a pooled analysis of community-based studies. J Am Soc Nephrol 2004;**15**(5):1307-15.
1225. Weiner DE, Tighiouart H, Stark PC, et al. Kidney disease as a risk factor for recurrent cardiovascular disease and mortality. Am J Kidney Dis 2004;**44**(2):198-206.
1226. Weissman AM, Levy BT, Hartz AJ, et al. Pooled analysis of antidepressant levels in lactating mothers, breast milk, and nursing infants. Am J Psychiatry 2004;**161**(6):1066-78.
1227. Welten CC, Koeter M, Wohlfarth TD, et al. Efficacy of drug treatment for acute mania differs across geographic regions: An individual patient data meta-analysis of placebo-controlled studies. J Psychopharmacol 2015;**29**(8):923-32.
1228. Welten CCM, Koeter MWJ, Wohlfarth T, et al. Placebo response in antipsychotic trials of patients with acute mania. Results of an individual patient data meta-analysis. Eur Neuropsychopharmacol 2015;**25**(7):1018-26.
1229. Westerhout CM, Bonnefoy E, Welsh RC, et al. The influence of time from symptom onset and reperfusion strategy on 1-year survival in ST-elevation myocardial infarction: a pooled analysis of an early fibrinolytic strategy versus primary percutaneous coronary intervention from CAPTIM and WEST. Am Heart J 2011;**161**(2):283-90.
1230. Wheatley-Price P, Blackhall F, Lee SM, et al. The influence of sex and histology on outcomes in non-small-cell lung cancer: a pooled analysis of five randomized trials. Ann Oncol 2010;**21**(10):2023-8.
1231. Wheatley-Price P, Ma C, Ashcroft LF, et al. The strength of female sex as a prognostic factor in small-cell lung cancer: a pooled analysis of chemotherapy trials from the Manchester Lung Group and Medical Research Council Clinical Trials Unit. Ann Oncol 2010;**21**(2):232-7.
1232. Whegang Youdom S, Samson A, Basco LK, et al. Multiple treatment comparisons in a series of anti-malarial trials with an ordinal primary outcome and repeated treatment evaluations. Malar J 2012;**11**:147.
1233. White WB, West CR, Borer JS, et al. Risk of cardiovascular events in patients receiving celecoxib: a meta-analysis of randomized clinical trials. Am J Cardiol 2007;**99**(1):91-8.
1234. Whitehead A, Perdomo C, Pratt RD, et al. Donepezil for the symptomatic treatment of patients with mild to moderate Alzheimer's disease: a meta-analysis of individual patient data from randomised controlled trials.[see comment]. [Review] [40 refs]. Int J Geriatr Psychiatry 2004;**19**(7):624-33.
1235. Whiteley WN, Adams HP, Jr., Bath PMW, et al. Targeted use of heparin, heparinoids, or low-molecular-weight heparin to improve outcome after acute ischaemic stroke: an individual patient data meta-analysis of randomised controlled trials. Lancet Neurol 2013;**12**(6):539-45.
1236. Whittmore AS, Harris R, Itnyre J. Characteristics relating to ovarian cancer risk: collaborative analysis of 12 US case-control studies II. Invasive epithelial ovarian cancers in white women. Am J Epidemiol 1992;**136**(10):1184-203.

1237. Wilcock GK, Birks J, Whitehead A, et al. The effect of selegiline in the treatment of people with Alzheimer's disease: a meta-analysis of published trials. Int J Geriatr Psychiatry 2002;**17**(2):175-83.
1238. Williams CJ, Stewart L, Parmar M, et al. Meta-analysis of the role of platinum compounds in advanced ovarian carcinoma. The Advanced Ovarian Cancer Trialists Group. Semin Oncol 1992;**19**(1 Suppl 2):120-8.
1239. Williamson PR, Marson AG, Tudur C, et al. Individual patient data meta-analysis of randomized anti-epileptic drug monotherapy trials. J Eval Clin Pract 2000;**6**(2):205-14.
1240. Willmann O, Wennberg R, May T, et al. The role of magnetic resonance spectroscopy in pre-operative evaluation for epilepsy surgery. A meta-analysis. Epilepsy Res 2006;**71**(2-3):149-58.
1241. Woodward M, Barzi F, Feigin V, et al. Associations between high-density lipoprotein cholesterol and both stroke and coronary heart disease in the Asia Pacific region. Eur Heart J 2007;**28**(21):2653-60.
1242. Woodward M, Huxley R, Lam TH, et al. A comparison of the associations between risk factors and cardiovascular disease in Asia and Australasia. Eur J Cardiovasc Prev Rehabil 2005;**12**(5):484-91.
1243. Woodward M, Martiniuk A, Ying Lee CM, et al. Elevated total cholesterol: its prevalence and population attributable fraction for mortality from coronary heart disease and ischaemic stroke in the Asia-Pacific region. Eur J Cardiovasc Prev Rehabil 2008;**15**(4):397-401.
1244. Woodward M, Zhang X, Barzi F, et al. The effects of diabetes on the risks of major cardiovascular diseases and death in the Asia-Pacific region. Diabetes Care 2003;**26**(2):360-6.
1245. Woopen H, Richter R, Chekerov R, et al. The influence of comorbidity and comedication on grade III/IV toxicity and prior discontinuation of chemotherapy in recurrent ovarian cancer patients: An individual participant data meta-analysis of the North-Eastern German Society of Gynecological Oncology (NOGGO). Gynecol Oncol 2015.
1246. Worldwide Antimalarial Resistance Network (WWARN) AL Dose Impact Study Group. The effect of dose on the antimalarial efficacy of artemether-lumefantrine: a systematic review and pooled analysis of individual patient data. Lancet Infect Dis 2015;**15**(6):692-702.
1247. WorldWide Antimalarial Resistance Network (WWARN) AS-AQ Study Group. The effect of dosing strategies on the therapeutic efficacy of artesunate-amodiaquine for uncomplicated malaria: a meta-analysis of individual patient data. BMC Med 2015;**13**:66.
1248. Wormser D, Kaptoge S, Di Angelantonio E, et al. Separate and combined associations of body-mass index and abdominal adiposity with cardiovascular disease: collaborative analysis of 58 prospective studies. Lancet 2011;**377**(9771):1085-95.
1249. Wu IC, Lin CC, Hsiung CA, et al. Epidemiology of sarcopenia among community-dwelling older adults in Taiwan: A pooled analysis for a broader adoption of sarcopenia assessments. Geriatr Gerontol Int 2014;**14**(SUPPL.1):52-60.
1250. Wu Y-L, Zhong W-Z, Li L-Y, et al. Epidermal growth factor receptor mutations and their correlation with gefitinib therapy in patients with non-small cell lung cancer: A meta-analysis based on updated individual patient data from six medical centers in mainland China. J Thorac Oncol 2007;**2**(5):430-39.
1251. Xing Y, Bronstein Y, Ross MI, et al. Contemporary diagnostic imaging modalities for the staging and surveillance of melanoma patients: a meta-analysis. J Natl Cancer Inst 2011;**103**(2):129-42.
1252. Xu GJ, Li ZJ, Ma JX, et al. Anterior versus posterior approach for treatment of thoracolumbar burst fractures: A meta-analysis. Eur Spine J 2013;**22**(10):2176-83.
1253. Yablon SA, Brin MF, VanDenburgh AM, et al. Dose response with onabotulinumtoxinA for post-stroke spasticity: a pooled data analysis. Mov Disord 2011;**26**(2):209-15.
1254. Yang Y, Wang X, Mao Q, et al. Survival analysis for valproic acid use in adult glioblastoma multiforme: A meta-analysis of individual patient data and a systematic review. Seizure 2014;**23**(10):830-35.
1255. Yap YG, Duong T, Bland M, et al. Potential demographic and baselines variables for risk stratification of high-risk post-myocardial infarction patients in the era of implantable cardioverter-defibrillator - A prognostic indicator. Int J Cardiol 2008;**126**(1):101-07.
1256. Yap YG, Duong T, Bland M, et al. Temporal trends on the risk of arrhythmic vs. non-arrhythmic deaths in high-risk patients after myocardial infarction: A combined analysis from multicentre trials. Eur Heart J 2005;**26**(14):1385-93.
1257. Yatsuya H, Toyoshima H, Yamagishi K, et al. Body mass index and risk of stroke and myocardial infarction in a relatively lean population: meta-analysis of 16 Japanese cohorts using individual data. Circ Cardiovasc Qual Outcomes 2010;**3**(5):498-505.
1258. Yau JWY, Rogers SL, Kawasaki R, et al. Global prevalence and major risk factors of diabetic retinopathy. Diabetes Care 2012;**35**(3):556-64.
1259. Yazici C, Mutlu E, Bonkovsky HL, et al. Risk factors for severe or fatal drug-induced liver injury from amoxicillin-clavulanic acid. Hepatol Res 2015;**45**(6):676-82.

1260. Yetgin S, Olcay L, Dibar E, et al. Systematic review of the addition of vincristine plus steroid pulses in maintenance treatment for childhood acute lymphoblastic leukaemia - an individual patient data meta-analysis involving 5659 children. Br J Haematol 2010;**149**(5):722-33.
1261. Yothers G, Sargent DJ, Wolmark N, et al. Outcomes among black patients with stage II and III colon cancer receiving chemotherapy: an analysis of ACCENT adjuvant trials. J Natl Cancer Inst 2011;**103**(20):1498-506.
1262. Young J, De SA, Merenstein D, et al. Antibiotics for adults with clinically diagnosed acute rhinosinusitis: a meta-analysis of individual patient data. Lancet 2008;**371**(9616):908-14.
1263. Yusuf S, Zucker D, Peduzzi P, et al. Effect of coronary artery bypass graft surgery on survival: overview of 10-year results from randomised trials by the Coronary Artery Bypass Graft Surgery Trialists Collaboration.[see comment][erratum appears in Lancet 1994 Nov 19;344(8934):1446]. Lancet 1994;**344**(8922):563-70.
1264. Zhang H, Li P, Ju H, et al. Diagnostic and prognostic value of microRNA-21 in colorectal cancer: an original study and individual participant data meta-analysis. Cancer Epidemiology, Biomarkers and Prevention 2014;**23**(12):2783-92.
1265. Zhang X, Patel A, Horibe H, et al. Cholesterol, coronary heart disease, and stroke in the Asia Pacific region. Int J Epidemiol 2003;**32**(4):563-72.
1266. Zhao F-H, Lin MJ, Chen F, et al. Performance of high-risk human papillomavirus DNA testing as a primary screen for cervical cancer: a pooled analysis of individual patient data from 17 population-based studies from China.[Erratum appears in Lancet Oncol. 2011 Jan;12(1):11]. Lancet Oncol 2010;**11**(12):1160-71.
1267. Zhao X-Q, Krasuski RA, Baer J, et al. Effects of combination lipid therapy on coronary stenosis progression and clinical cardiovascular events in coronary disease patients with metabolic syndrome: a combined analysis of the Familial Atherosclerosis Treatment Study (FATS), the HDL-Atherosclerosis Treatment Study (HATS), and the Armed Forces Regression Study (AFREGS). Am J Cardiol 2009;**104**(11):1457-64.
1268. Zhou BF. Effect of body mass index on all-cause mortality and incidence of cardiovascular diseases--report for meta-analysis of prospective studies open optimal cut-off points of body mass index in Chinese adults. Biomed Environ Sci 2002;**15**(3):245-52.
1269. Ziegler R, Grossarth-Maticek R. Individual patient data meta-analysis of survival and psychosomatic self-regulation from published prospective controlled cohort studies for long-term therapy of breast cancer patients with a mistletoe preparation (iscador). Evid Based Complement Alternat Med 2010;**7**(2):157-66.
1270. Zinkstok SM, Vergouwen MDI, Engelter ST, et al. Safety and functional outcome of thrombolysis in dissection-related ischemic stroke: a meta-analysis of individual patient data. Stroke 2011;**42**(9):2515-20.
1271. Zinman B, Schmidt WE, Moses A, et al. Achieving a clinically relevant composite outcome of an HbA1c of <7% without weight gain or hypoglycaemia in type 2 diabetes: a meta-analysis of the liraglutide clinical trial programme. Diabetes Obes Metab 2012;**14**(1):77-82.
1272. Zong L, Chen P. Prognostic value of KIT/PDGFRA mutations in gastrointestinal stromal tumors: a meta-analysis. World J Surg Oncol 2014;**12**:71.
1273. Zong L, Seto Y. CpG island methylator phenotype, Helicobacter pylori, Epstein-Barr virus, and microsatellite instability and prognosis in gastric cancer: A systematic review and meta-analysis. PLoS ONE [Electronic Resource] 2014;**9**(1).
1274. Zugna D, Galassi C, Annesi-Maesano I, et al. Maternal complications in pregnancy and wheezing in early childhood: A pooled analysis of 14 birth cohorts. Int J Epidemiol 2015;**44**(1):199-208.
1275. Zusterzeel R, Selzman KA, Sanders WE, et al. Cardiac resynchronization therapy in women: US Food and Drug Administration meta-analysis of patient-level data. JAMA Intern Med 2014;**174**(8):1340-8.
1276. Zwang J, Ashley EA, Karema C, et al. Safety and efficacy of dihydroartemisinin-piperaquine in falciparum malaria: a prospective multi-centre individual patient data analysis. PLoS One 2009;**4**(7):e6358.
1277. Zwang J, Dorsey G, Djimde A, et al. Clinical tolerability of artesunate-amodiaquine versus comparator treatments for uncomplicated falciparum malaria: an individual-patient analysis of eight randomized controlled trials in sub-Saharan Africa. Malaria Journal 2012;**11**:260.
1278. Zwang J, Dorsey G, Mårtensson A, et al. Plasmodium falciparum clearance in clinical studies of artesunate-amodiaquine and comparator treatments in sub-Saharan Africa, 1999-2009. Malaria Journal 2014;**13**(1).
