# Supplementary material for: Exploring changes over time and characteristics associated with data retrieval across individual participant data meta-analyses: systematic review
Source: BMJ. 2017 Apr 5;357:j1390. doi: 10.1136/bmj.j1390 (PMC5733815; doi:10.1136/bmj.j1390)
Supplement: Supplementary file 5 — Appendix 5 : Outcome of IPD requests [file nevs036543.ww5.pdf]

| Number of studies and participants requested[1] | Original requests (approx. 1995 – 2005) by type of study | | | | New requests (2012-2015) by type of study | | | | All requests (approx. 1995-2015) by type of study | | | |
|---|---|---|---|---|---|---|---|---|---|---|---|---|
| | **Ac** | **Go** | **Ph** | **Total** | **Ac** | **Go** | **Ph** | **Total** | **Ac** | **Go** | **Ph** | **Total** |
| Eligible studies | 15 | 4 | 10 | **29** | 24 | 1 | 14 | **39** | 39 | 5 | 24 | **68** |
| Studies providing IPD (n (%)) | 6 (40%) | 3 (75%) | 9 (90%) | **18 (62%)** | 7 (29%) | 0 (0%) | 8 (57%) | **15 (38%)** | 13 (33%) | 3 (60%) | 17 (71%) | **33 (49%)** |
| Eligible participants | 1803 | 1178 | 2906 | **5887** | 1897 | 593 | 5771 | **8261** | 3700 | 1771 | 8677 | **14148** |
| Participants IPD is provided for (n (%)) | 1008 (56%) | 1091 (93%) | 2604 (90%) | **4703 (80%)** | 801 (42%) | 0 (0%) | 4534 (79%) | **5335 (65%)** | 1809 (49%) | 1091 (62%) | 7138 (82%) | **10038 (71%)** |
| **Reason data was not available: Number of studies (n (%))** | | | | | | | | | | | | |
| Data lost | 5 (33%) | 1 (25%) | 0 (0%) | **6 (21%)** | 3 (13%) | 0 (0%) | 0 (0%) | **3 (8%)** | 8 (21%) | 1 (20%) | 0 (0%) | **9 (13%)** |
| Relevant data not recorded | 2 (13%) | 0 (0%) | 0 (0%) | **2 (7%)** | 1 (4%) | 0 (0%) | 0 (0%) | **1 (3%)** | 3 (8%) | 0 (0%) | 0 (0%) | **3 (4%)** |
| Unable to make contact with an author / sponsor | 1 (7%) | 0 (0%) | 0 (0%) | **1 (3%)** | 11 (46%) | 0 (0%) | 0 (0%) | **11 (28%)** | 12 (31%) | 0 (0%) | 0 (0%) | **12 (18%)** |
| Positive response but no data received | 1 (7%) | 0 (0%) | 0 (0%) | **1 (3%)** | 1 (4%) | 1 (100%) | 0 (0%) | **2 (5%)** | 2 (5%) | 1 (20%) | 0 (0%) | **3 (4%)** |
| Incomplete dataset provided which could not be used | 0 (0%) | 0 (0%) | 1 (10%) | **1 (3%)** | 0 (0%) | 0 (0%) | 0 (0%) | **0 (0%)** | 0 (0%) | 0 (0%) | 1 (4%) | **1 (1%)** |
| Local authority / ethical restrictions | 0 (0%) | 0 (0%) | 0 (0%) | **0 (0%)** | 1 (4%) | 0 (0%) | 0 (0%) | **1 (3%)** | 1 (3%) | 0 (0%) | 0 (0%) | **1 (1%)** |
| "Data not available"[2] | 0 (0%) | 0 (0%) | 0 (0%) | **0 (0%)** | 0 (0%) | 0 (0%) | 3 (21%) | **3 (8%)** | 0 (0%) | 0 (0%) | 3 (13%) | **3 (4%)** |
| Costs of providing data are prohibitive | 0 (0%) | 0 (0%) | 0 (0%) | **0 (0%)** | 0 (0%) | 0 (0%) | 2 (14%) | **2 (5%)** | 0 (0%) | 0 (0%) | 2 (8%) | **2 (3%)** |
| Country specific restrictions | 0 (0%) | 0 (0%) | 0 (0%) | **0 (0%)** | 0 (0%) | 0 (0%) | 1 (7%) | **1 (3%)** | 0 (0%) | 0 (0%) | 1 (4%) | **1 (1%)** |
| **Total** | **9 (60%)** | **1 (25%)** | **1 (10%)** | **11 (38%)** | **17 (61%)** | **1 (100%)** | **6 (43%)** | **24 (62%)** | **26 (67%)** | **2 (40%)** | **7 (29%)** | **35 (51%)** |

**Supplementary Table 1: Outcome of individual participant data requests conducted between approx. 1995 to 2005 and 2012 to 2015.**

Abbreviations: Ac: Academic studies, Go: Government Studies, Ph: Pharmaceutical Studies

Footnotes

1. In addition, we had IPD available from our own 'SANAD' trial, [50 51] the largest ever in epilepsy at the time, which randomised 2437 participants
2. Refers to a non-specific reason (data not available for secondary analysis with no further reason provided).
