# Supplementary material for: Exploring changes over time and characteristics associated with data retrieval across individual participant data meta-analyses: systematic review
Source: BMJ. 2017 Apr 5;357:j1390. doi: 10.1136/bmj.j1390 (PMC5733815; doi:10.1136/bmj.j1390)
Supplement: Supplementary file 6 — Appendix figure: Characteristics of systematic IPD meta-analyses and proportion of IPD provided [file nevs036543.wf.pdf]

Number of systematic IPDMA and proportion of IPD provided by type of included study

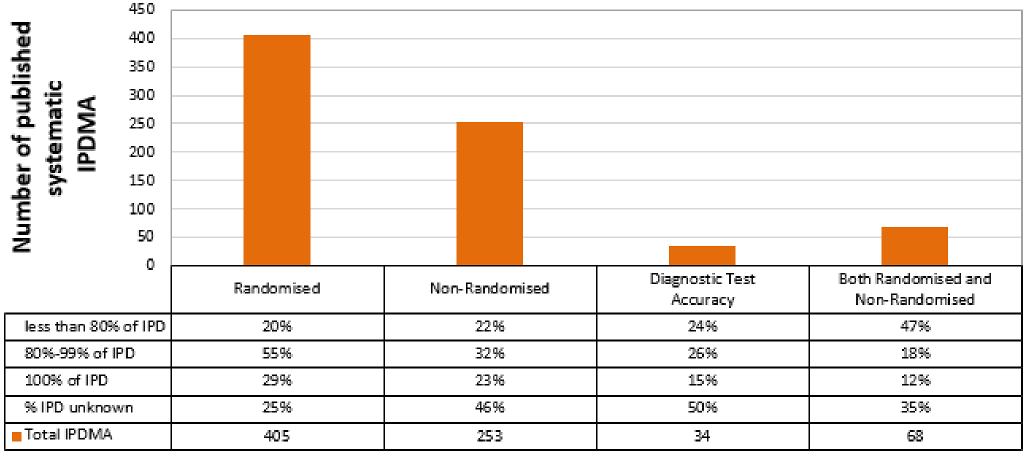

	Randomised	Non-Randomised	Diagnostic Test Accuracy	Both Randomised and Non-Randomised
less than 80% of IPD	20%	22%	24%	47%
80%-99% of IPD	55%	32%	26%	18%
100% of IPD	29%	23%	15%	12%
% IPD unknown	25%	46%	50%	35%
Total IPDMA	405	253	34	68

Number of systematic IPDMA and proportion of IPD in Cochrane and non Cochrane reviews

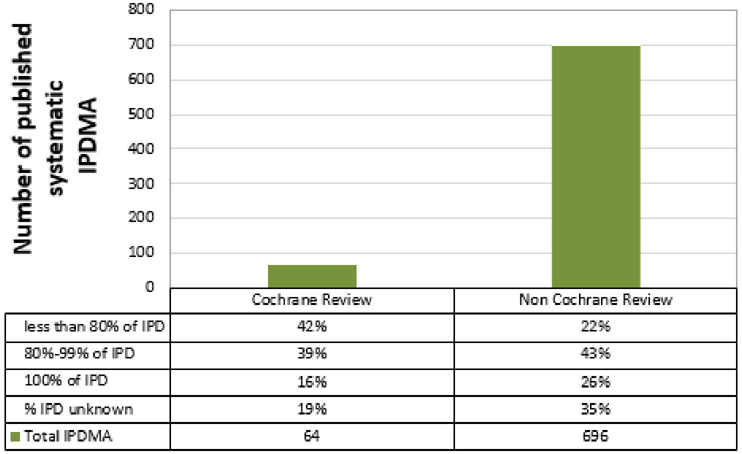

	Cochrane Review	Non Cochrane Review
less than 80% of IPD	42%	22%
80%-99% of IPD	39%	43%
100% of IPD	16%	26%
% IPD unknown	19%	35%
Total IPDMA	64	696

Number of systematic IPDMA and proportion of IPD provided by source of funding

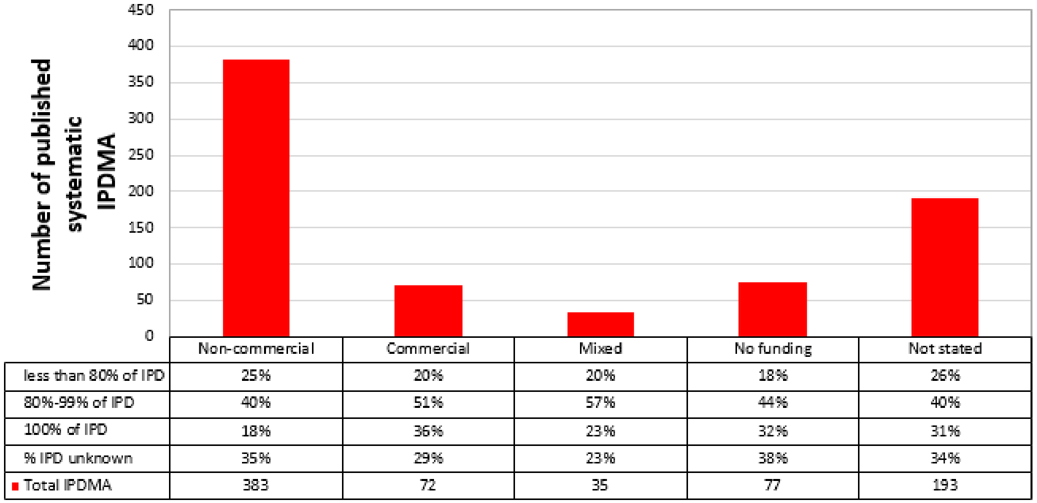

	Non-commercial	Commercial	Mixed	No funding	Not stated
less than 80% of IPD	25%	20%	20%	18%	26%
80%-99% of IPD	40%	51%	57%	44%	40%
100% of IPD	18%	36%	23%	32%	31%
% IPD unknown	35%	29%	23%	38%	34%
Total IPDMA	383	72	35	77	193

Number of systematic IPDMA and proportion of IPD provided by authorship policy

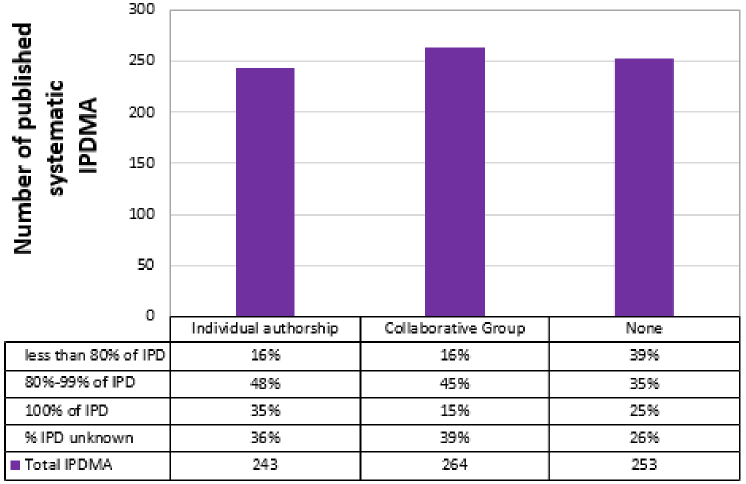

	Individual authorship	Collaborative Group	None
less than 80% of IPD	16%	16%	39%
80%-99% of IPD	48%	45%	35%
100% of IPD	35%	15%	25%
% IPD unknown	36%	39%	26%
Total IPDMA	243	264	253

Supplementary Figure 1: Characteristics of systematic IPDMA and proportion of IPD provided.

1. See Table 1 for proportion of systematic IPD meta-analyses providing 100%, 80-99%, less than 80% of IPD and the proportion of IPD not reported.
